# *SS1* (*NAL1*)- and *SS2-*Mediated Genetic Networks Underlying Source-Sink and Yield Traits in Rice (*Oryza sativa* L.)

**DOI:** 10.1371/journal.pone.0132060

**Published:** 2015-07-10

**Authors:** Jian-Long Xu, Yun Wang, Fan Zhang, Yuan Wu, Tian-Qing Zheng, Yong-Hong Wang, Xiu-Qin Zhao, Yan-Ru Cui, Kai Chen, Qiang Zhang, Hong-Xuan Lin, Jia-Yang Li, Zhi-Kang Li

**Affiliations:** 1 Institute of Crop Sciences/National Key Facility for Crop Gene Resources and Genetic Improvement, Chinese Academy of Agricultural Sciences, 12 South Zhong-Guan-Cun Street, Beijing 100081, China; 2 Shenzhen Institute for Innovative Breeding, Chinese Academy of Agricultural Sciences, Shenzhen 518120, China; 3 Rice Institute of Shenyang Agricultural University/Key Laboratory of Crop Physiology, Ecology, Genetics and Breeding, Ministry of Agriculture, Shenyang 110866, China; 4 Institute of Plant Physiology and Ecology, Shanghai Institutes for Biological Sciences, Chinese Academy of Sciences, Shanghai 200032, China; 5 Institute of Genetics and Developmental Biology, Chinese Academy of Sciences, Beijing 100101, China; International Rice Research Institute, PHILIPPINES

## Abstract

Source leaf/sink capacity (SS) traits are important determinants of grain yield (GY) of rice. To understand the genetic basis of the SS relationship in rice, five SS and GY traits of rice were genetically dissected using two reciprocal introgression populations. Seventy-three QTL affecting the SS and GY traits were identified, most of which were detected in one of the parental genetic backgrounds (GBs). Two major QTL at bins 4.7 (*SS1*) and 3.12 (*SS2*) were associated consistently with all measured SS and yield traits in both GBs across two contrasting environments. Strong interactions between *SS1*/*SS2* and the detected QTL led us to the discovery of genetic networks affecting the SS and GY traits. The *SS1* acted as a regulator controlling two groups of downstream QTL affecting the source leaf width and grain number per panicle (GNP). *SS2* functioned as a regulator positively regulating different groups of downstream QTL affecting the source leaf length, GNP, grain weight, and GY. Map-based cloning of *SS1* indicates that *SS1* is *NAL1* involved in polar auxin/IAA transport. Different alleles at *NAL1* were apparently able to qualitatively and/or quantitatively control the IAA transport from the apical meristem to different plant tissues and thus regulate those downstream loci/pathways controlling different SS traits of rice. There was a functional allele and a non-functional mutation in the parents at each of the QTL downstream of *SS1* or *SS2*, which were detectable only in the presence of the functional allele of *SS1* or *SS2*. Our results provided direct evidence that SS and yield traits in rice are controlled by complex signaling pathways and suggest further improvement of rice yield potential with enhanced and balanced SS relationships can be achieved by accurately manipulating allelic combinations at loci in the *SS1* and *SS2* mediated pathways.

## Introduction

Rice (*Oryza sativa* L) is the staple food of most Asian people. Rice productivity has been more than tripled in China, resulting primarily from the Green Revolution since late 1950s and the hybrid rice technology in late 1970s. However, progress has been slow for more than 2 decades to further improve yield potentials of modern rice cultivars despite tremendous breeding efforts. Plant physiologists believe that high yield potentials of cereal crops are largely determined by enhanced and balanced relationships between the source, sink and flow of assimilates [1, 2]. In rice, grain number per panicle (GNP) and grain size are the primary components of the sink capacity for photosynthetic product accumulation. The upper three leaves, especially the flag leaves, are the primary source of assimilate-supply for grain yield [3–5]. Besides, efficient transport of assimilates from leaves and stems to developing grains is also important for better grain filling and high yield [6, 7]. Therefore, characterizing genes/QTL underlying the sink-source (SS) relationship will greatly enhance our understanding of the genetic basis of yield potential in rice.

Past decades have witnessed tremendous efforts in mapping QTL affecting SS and yield traits in rice. Results from these studies indicate that rice yield and its components are controlled by large numbers of QTL across the rice genome, and influenced by complex epistasis and QTL × environment interactions[8–20]. An important discovery is that QTL affecting yield traits differ greatly in their phenotypic effects and QTL of large effect tend to be associated with multiple related traits, forming QTL clusters [21, 22]. To date, many large-effect QTL affecting yield traits in rice have been cloned [23–29]. In most cases, the large phenotypic effect(s) at each of the cloned QTL result from the allelic difference between a functional allele and a loss or partial loss of function mutant, though the cloned yield QTL genes have diverse molecular functions [22–29]. Meanwhile, several cases of marker-assisted transfer of large-effect QTL for improving complex traits have been reported with mixed results. Very often, the phenotypic effects of these large-effect QTL tend to vary considerably across genetic backgrounds (GBs) and environments [30–36]. To date, no successful story for developing new high yielding rice varieties has been reported by marker-assisted selection (MAS) of large-effect QTL. While these large-effect QTL represent only a very small portion of the genes that contribute to genetic variation of complex traits, most reported QTL in rice have moderate effects that vary considerably across different environments and GBs and tend to be involved in complex epistasis [15, 19–21, 37]. Meanwhile, few efforts have been taken to confirm and characterize ‘small-effect QTL’ by cloning or to use them as target QTL for trait improvement in MAS experiments. Further, we found that most QTL affecting grain shape and size traits of rice were detectable only in one of the parental GBs [38]. This raises a serious question on the classic quantitative genetics theory which predicts that all main-effect QTL should be detectable in both parental GBs and QTL main effects and epistatic effects in the model are independent from one another [37, 39]. Theoretically, linking phenotypic variation of complex traits to its underlying genes and gene networks have been the greatest challenge because the classical quantitative/population genetics models and relevant statistical methodology have a limited power to detect and characterize high-order epistasis, which is predicted to exist based on the current knowledge of molecular genetics. Recently, we developed the theoretical framework of molecular-quantitative (function-mutation) genetics models that allow detection and characterization of genetic networks underlying complex traits [40]. In other words, genes involved in the same signaling pathways segregating in a mapping population can be detected as “genetic networks” based on the epistatic and predicted regulatory—regulated relationships between QTLs affecting a complex trait either using either the quantitative genetics approach or population genetics approach [20, 41, 42].

Here, we validated the theory by detecting and characterizing the genetic networks underlying SS and grain yield traits of rice using two reciprocal introgression line (IL) populations, and by map-based cloning one large-effect QTL that acted as a regulator in the upstream of the auxin/IAA mediated signaling pathways controlling SS and yield traits in rice. Our results provide direct evidence for the presence of complex genetic networks controlling complex traits in rice and novel breeding strategies to improve rice yield potential through accurate manipulation of key regulatory loci and their downstream ones in rice.

## Materials and Methods

### 2.1. Detecting the genetic networks underlying SS and yield traits

#### Materials

Lemont (LT), a commercial semidwarf *japonica* rice variety from Southern US was used as the female parent to cross with Teqing (TQ), a high-yielding semidwarf *indica* rice variety from China. The F_1_ plants were backcrossed to TQ to develop a BC_1_F_1_ population with 100 plants. The BC_1_F_1_ plants were used as the male parent to backcross with TQ to produce the BC_2_F_1_ population. Consecutive backcrossing was carried out in the same way until BC_3_F_1_ and BC_4_F_1_ populations followed by several generations of selfing, resulting in a set of 254 TQ-ILs, consisting of 133 BC_2_F_8_, 96 BC_3_F_7_ and 25 BC_4_F_6_ lines [43]. The procedure for developing 201 LT introgression lines was similar using LT as the recurrent parent. The LT-ILs consisted of 32 BC_2_F_8_, 123 BC_3_F_7_ and 46 BC_4_F_6_ lines [15].

#### Phenotyping experiments

The two sets of 455 reciprocal ILs and parents were evaluated for SS and GY traits in Sanya (18.3° N, 109.3° E) in the 2007 winter season and Beijing (40.2° N, 116.2° E) in the 2008 summer season. In both Beijing and Sanya, seeds of each lines were sown on the seedling nursery and 30-day-old seedlings of each line were transplanted into a 2-row plot with 12 plants per row at a spacing of 20 × 17 cm in the field of the experimental farms, Institute of Crop Sciences, Chinese Academy of Agricultural Sciences. A randomized block design was used in each experiment with 3 replications for each line. Two applications of insecticides were applied to control brown planthoppers in the SY experiment. Weeds were controlled by a combination of chemical and manual methods. Measurements for the source leaf traits, flag leaf width (FLW, in mm) and flag leaf length (FLL, in cm), were taken on 3 main stems of each plant in the middle 10 plants of each plot. At the maturity, ten representative plants in each plot were sampled and dried under 72°C for 3 days in an oven. Then, the sampled plants were measured for grain number per panicle (GNP), 1000-grain weight (GW, in g) and grain yield per plant (GY, in g).

#### Genotyping and linkage map construction

The two sets of ILs were genotyped with the same set of 478 well distributed DNA markers, including 151 simple sequence repeat (SSR) markers, 3 morphological markers and 324 well distributed anchor single nucleotide polymorphic markers (SNPs) (S1 Table) [44]. Two linkage maps were constructed separately from the LT-ILs and TQ-ILs using the MAPMAKER version 3.0. The two resulting linkage maps constructed from the reciprocal IL sets were very similar with 1734.4 cM for the TQ-ILs and 1661.0 cM for the LT-ILs, respectively (S1 Fig). The LT-ILs had an average 83.8±15.5% the LT genome. The TQ-ILs had an average 88.9±8.1% of the TQ genome.

#### Detecting QTL and QTL networks affecting the SS and GY traits

QTL affecting the SS and GY traits in each of the IL populations were identified by detecting the trait-marker associations using the phenotypic data obtained from each environment and single marker analyses using the SAS PROC GLM [45], in which *y*_*k*_ = *μ* + *a*_*i*_ *x*_*ik*_ + *e*, where *y*_*k*_ is the phenotypic value of a trait measured on the *k*th individual; *μ* is the population mean; *a*_*i*_ is the main effect of the one putative QTL; *x*_*ik*_ is coefficient of QTL effect derived according to the observed genotypes of the markers; *e* ~*N*(0,σ_*e*_^2^) is the random residual effect. A significance level of *P* < 0.005 was used as the threshold to claim a main-effect QTL in at least one of the population/environment combinations. The putative genetic networks consisting of interacting QTL affecting the SS and GY traits were constructed based on the new function-mutation model of the quantitative genetics theory [20, 40]. In this model, two new concepts, functional genetic units (FGUs) and the principle of hierarchy are defined based on two commonest types of functional relationships between genes acting in a signaling pathway affecting complex traits. An FGU represents the mutual functional dependency (FD) among a group of genes functioning at each level of a signaling pathway which affect phenotype(s) in a manner of “house of cards” or complete complementarity. Hierarchy reflects the one-way FD of genes in downstream pathways on their upstream regulator(s). Epistasis is predicted to result from these two types of FD between or among unlinked loci within a signaling pathway. Based on this model, genetic networks underlying the SS and yield traits were detected in 3 steps. First, two-way ANOVA was conducted to detect all significant pairwise epistasis between QTL identified in each of the population/environment combinations with a threshold of *P* ≤ 0.005 in at least one of the population/environment combinations, in which *y*_*k*_ = *μ* + *a*_*i*_ *x*_*ik*_ + *a*_*j*_ *x*_*jk*_ + *aa*_*ij*_ *x*_*ijk*_ + *e*, where *y*_*k*_ is the phenotypic value of a trait measured on the *k*th individual; *μ* is the population mean; *a*_*i*_ and *a*_*j*_ are the main effects of the two putative QTL (*Q*_*i*_ and *Q*_*j*_), respectively; *aa*_*ij*_ is the epistatic effect between *Q*_*i*_ and *Q*_*j*_; *x*_*ik*_, *x*_*jk*_, and *x*_*ijk*_ are coefficients of QTL effects derived according to the observed genotypes of the markers; *e* ~*N*(0,σ_*e*_^2^) is the random residual effect. Second, once significant epistasis was detected between two QTL, their relationship and the nature of the parental alleles (a functional one vs a non-functional mutant) were then determined either as one-way FD (one QTL is in the upstream and the other in the downstream) or as the mutual FD (complementarity) by examining if the observed mean trait values of the digenic genotypes fit the expected patterns of the corresponding theoretical models. Third, the pathway effect of each interacting QTL pair was estimated based on the genetic expectations of the multi-locus genotypes and the obtained QTL main and epistatic effects [20, 40].

### 2.2. Map-based cloning of *SS1*

The following experiments were conducted to clone a major QTL, *SS1*, on rice chromosome 4 that had large and consistent effects on SS traits (FLW and GNP):

#### Development of near isogenic lines (NILs) and fine mapping of *SS1*

S2 Fig shows the detailed process in fine mapping *SS1*. Briefly, a BC_2_F_5_ TQ-IL (GG253) carrying the homozygous LT introgression in the *SS1* region flanked by RM317–RM348 on chromosome 4 and 90.5% of the TQ GB was selected to cross with TQ. The resulting 25 BC_3_F_1_ plants were backcrossed to TQ to produce 385 BC_4_F_1_ plants. The 4 BC_4_F_1_ plants were selfed to generate a 650 BC_4_F_2_ population. Six plants with heterozygous genotype at the target region on chromosome 4 and homozygous Lemont genotypes at all other non-target regions were selected in the BC_4_F_2_ based on foreground and background selections with 550 SSR markers across the 12 rice chromosomes. In the 2009 winter season, 6,000 BC_4_F_3_ Plants were planted in the CAAS experimental farm in Sanya. We developed some insertions and deletions (InDel) and cleaved amplified polymorphic sequences (CAPS) markers in the target region designed from the reference Nipponbare and 93–11 genomic sequences (S2 Table) and determined genotypes of the recombinants with these markers. Fifteenplants heterozygous at the target *SS1* region and homozygous TQ genotypes at all non-target regions were selected. The 12,000 BC_4_F_4_ segregating individuals were genotyped to identify recombinants in the *SS1* region for further fine mapping *SS1*. The same process was repeated and phenotypic comparisons in FLW and GNP were made between the two parental homozygous recombinants identified inside the target region in the progeny to gradually narrow down the target *SS1* region. Finally, a NIL*-SS1* containing the LT alleles at *SS1* in a small region of 50.3 kb flanked by markers RM3534 and FL98 on chromosome 4 was identified in the TQ BC_4_F_5_ population.

#### Sequence analysis

The parents (TQ and LT) and the NIL-*SS1*were sequenced for 3 positional candidate genes in the 50.3 kb target region and their promoter regions upstream the transcription starting site. DNA fragments from the materials were amplified with high fidelity using a LA-Taq kit (TakaRa, Dalian, China). The PCR products were cloned into a pGEM-T vector (Promega, USA) according to the manufacturer’s specification. The T7 and SP6 universal primers and BigDye Terminator Cycle Sequencing v3.1 (Applied Biosystems, CA, USA) were used for sequencing. Sequence contigs were assembled using the computer program SEQUENCHER 4.1.2. Sequence alignment was conducted using computer program Clustalx 1.83.

#### Gene expression analysis

Total RNA from young panicles, flag leaves, leaf sheaths, nodes, internode, roots, root and stem junctions of the parents and NIL-*SS1* was extracted at the panicle initiation stage using the TRIzol Reagent Kit (Invitrogen, Carlsbad, USA) and treated with DNase I. cDNA was synthesized from 2 μg RNA using SuperScript III Reverse Transcriptase. Quantitative analyses of expression of the candidate genes in different tissues were performed using SYBR Premix Ex TaqTM on an Applied Biosystems 7500 Real-Time PCR System. The relative expression of each transcript was obtained by comparison with the expression of the rice gene ubiquitin. The primers used for the quantitative real-time PCR are listed in S3 Table.

#### Phenotypic characterization

The NIL-*SS1* and parents (LT and TQ) were evaluated for their agronomic performances under the normal irrigated field conditions two locations, Hangzhou (30.3° N, 120.2 ° E), Beijing and Sanya in 3 seasons from 2010–2012. The plants were grown in plots of 13.2 m^2^ at a space of 16.5 cm between plants within a row and 25 cm between rows in a randomized complete block design with three replications for each of the NIL-*SS1* and parents. The field management followed the standard rice cultivation practices. Eight plants in each plot were sampled and measured for yield and its component traits, including plant height (PH, in cm), productive panicles per plant (PP), FLW, FLL, panicle length (PL, in cm), GNP, filled grains per panicle (FGP), GW, grain length (GL, in mm), grain width (GWH, in mm), GY. Grain yield per plot in kg was measured by harvesting all plants in each plot. Also, the NIL-*SS1* and parents were assayed for physiological traits related to yield, including photosynthesis rate (*P*_*n*_), stomata conductance (*g*_*s*_), intercellular CO_2_ concentration (*C*_*i*_), transpiration (*T*_*r*_) and specific leaf weight, at the booting, flowering, grain filling at 7, 14 and 21 days after flowering (DAF) during 9:00–11:00 am on clear days using a portable gas analysis system LI-6400. Accumulation of dry matter in panicles and rate of dry matter translocation in stems and sheaths of the NIL-*SS1* and TQ were assayed between at the booting, flowering, grain filling at 7, 14 and 21 DAF.

## Results

### 3.1. Identification of the genetic networks underlying the SS and yield traits

#### Phenotypic variation of the reciprocal ILs

The parents differed significantly for all traits except for GW in both Sanya and Beijing (S4 Table). TQ had longer FLL, more GNP and slender FLW than LT. It had 178.5% and 187.1% higher yield than LT in Sanya and Beijing. Both the LT-ILs and TQ-ILs showed continuous variations with transgressive segregations toward both directions for all traits except for GY. ANOVA indicated that the differences among different genotypes (G) within each set of ILs were highly significant for all measured SS traits and explained an average of 48.7±15.8% of the total phenotypic variation in the IL populations, ranging from 21.0% for GY to 68.0% for GW (S5 Table). The difference between the two locations (E) was also highly significant for all traits except for GW in the LT-ILs and explained an average 16.5±14.3% of the total phenotypic variation in the IL populations, ranging from 0.1% for GW to 37.6% for GY. G x E interaction was also significant for all measured traits but explained an average 15.3±3.0% of the total phenotypic variation in the IL populations.

#### Main-effect QTL (M-QTL) for SS and yield traits

Forty-one M-QTL affecting SS and GY traits in 22 regions of the rice genome were identified in the TQ-ILs (S6 Table and S1 Fig). These included 14 FLW M-QTL, 9 FLL M-QTL, 8 GNP M-QTL, 8 GW M-QTL and 2 GY M-QTL. For the 14 FLW M-QTL, the LT alleles increased FLW at all M-QTL except for *qFlw3*.*5*, *qFlw6*.*5*, *qFlw9*.*1* and *qFlw10*.*6*. For the FLL M-QTL, the LT alleles increased FLL at *qFll1*.*8*, *qFll2*.*6*, *qFll3*.*5*, *qFll4*.*1*, *qFll8*.*4* and *qFll11*.*3*, but reduced FLL at *qFll3*.*12*, *qFll9*.*7*, and *qFll12*.*4*. The LT alleles increased GNP at *qGnp4*.*7*, *qGnp5*.*5*, *qGnp6*.*7a*, but reduced GNP at *qGnp1*.*8*, *qGnp3*.*12*, *qGnp4*.*1*, *qGnp8*.*4a* and *qGnp10*.*6*. At the GW QTL, the LT alleles increased GW at *qGw2*.*4*, *qGw3*.*12a*, *qGw7*.*5*, and *qGw8*.*4*, but reduced GW at *qGw1*.*8*, *qGw4*.*1*, *qGw5*.*5* and *qGw9*.*7*. The TQ alleles increased GY at both GY QTL (*qGy4*.*1* and *qGy9*.*7*). Two large QTL clusters at bins 3.12 and 4.7 were detected with large LOD scores in both environments. The former had large and consistent effects on FLL, GNP and GW. The latter had large effects on FLW and GNP.

Twenty-eight M-QTL in 17 genomic regions affecting the SS and GY traits were identified in the LT-ILs (S6 Table and S1 Fig). These included 8 FLW M-QTL with the LT alleles increased FLW at *qFlw2*.*2*, *qFlw4*.*7* and *qFlw12*.*4*, and reduced FLW at *qFlw1*.*8*, *qFlw5*.*5*, *qFlw6*.*5*, *qFlw7*.*1* and *qFlw12*.*2b*. For the five identified FLL M-QTL, the LT alleles increased FLL at *qFll6*.*7* and *qFll9*.*3*, but reduced FLL at *qFll2*.*9*, *qFll3*.*12* and *qFll12*.*4*. Eight GNP M-QTL were detected. The LT alleles increased GNP at *qGnp4*.*7*, *qGnp8*.*4b* and *qGnp9*.*7*, but reduced GNP at *qGnp2*.*9*, *qGnp3*.*5*, *qGnp3*.*12*, *qGnp6*.*3*, and *qGnp6*.*7b*. Three GW M-QTL were identified. The LT alleles increased GW at *qGw3*.*12a* and *qGw7*.*5*, but reduced GW at *qGw4*.*7*. Four GY M-QTL were identified. The TQ alleles at *qGy3*.*12*, *qGy8*.*4* and *qGy9*.*3* increased GY, but reduced GY at *qGy12*.*2*. Again, two large QTL clusters affecting the SS and GY traits were detected at bins 4.7 and 3.12 in the TQ-ILs.

#### GB effects of the detected M-QTL and their interactions with the environments

The identified M-QTL showed strong GB effects (S6 and S7 Tables). Of the total 69 detected M-QTL, only 5 M-QTL of large effect at bins 3.12 (*qFll3*.*12*, *qGnp3*.*12* and *qGw3*.*12a*) and 4.7 (*qFlw4*.*7* and *qGnp4*.*7*) were consistently detected in both GBs and environments, though their effects varied considerably in different GBs. The two QTL clusters at bins 4.7 and 3.12 are designated as *SS1* and *SS2*. Four other M-QTL (*qFlw6*.*5*, *qGw7*.*5*, *qFll12*.*4* and *qFlw12*.*4*) had consistent effects in both GBs, but *qFlw6*.*5* was detectable only in Sanya whereas *qGw7*.*5* only in Beijing. Bin 12.2 was associated with FLW in both IL populations, but the LT allele at this region increased FLW in Sanya by 0.8 mm in the TQ-ILs and reduced FLW by 0.7 mm in the LT-ILs in Sanya, suggesting the presence of two different FLW M-QTL (*qFlw12*.*2a* and *qFlw12*.*2b*) in this region. Similarly, bins 6.7 and 8.4 were associated with GNP in both IL populations with the LT allele at bin 8.4 increased GNP in the LT-ILs but reduced GNP in the TQ-ILs, and the reverse was true for bin 6.7, again suggesting the presence of two different GNP M-QTL in each of the regions.

Of the 41 M-QTL identified in the TQ-ILs, 23 (*qFll1*.*8*, *qGnp1*.*8*, *qFlw2*.*4*, *qFll2*.*6*, *qFll3*.*5*, *qFll4*.*1*, *qGw4*.*1*, *qGy4*.*1*, *qGnp5*.*5*, *qFlw6*.*3*, *qFlw6*.*5*, *qGnp6*.*7a*, *qGw7*.*5*, *qFlw8*.*4*, *qFlw9*.*1*, *qFll9*.*7*, *qFlw10*.*6*, *qGnp10*.*6*, *qFll11*.*3*, *qFlw11*.*7*, *qFlw12*.*2a*, *qFll12*.*4*, and *qFlw12*.*4*) were detected only in one of the environments. Of the 28 M-QTL identified in the LT-ILs, 15 (*qFlw1*.*8*, *qFll2*.*9*, *qGnp2*.*9*, *qGnp3*.*5*, *qGnp6*.*3*, *qFlw6*.*5*, *qGnp6*.*7b*, *qFll6*.*7*, *qFlw7*.*1*, *qGw7*.*5*, *qFll9*.*3*, *qGy9*.*3*, *qGnp9*.*7*, *qFlw12*.*2b* and *qGy12*.*2*) were detected only in one of the environments (S6 Table). Interestingly, 11 of those M-QTL (the underlined ones) became detectable in both environments when involved in epistasis (S7 Table).

#### The genetic networks underlying the SS and yield traits

S7 Table shows 87 highly significant interactions between 46 QTL, 34 of which were the identified M-QTL plus 12 additional epistatic QTL. All, except three (*qFll3*.*12* vs *qFll11*.*3*, *qFll3*.*12* vs *qFll8*.*4* and *qGnp4*.*7* vs *qGnp1*.*8*), of these interactions were detected only in one of the parental GBs, but in both environments.

**Fig 1:**
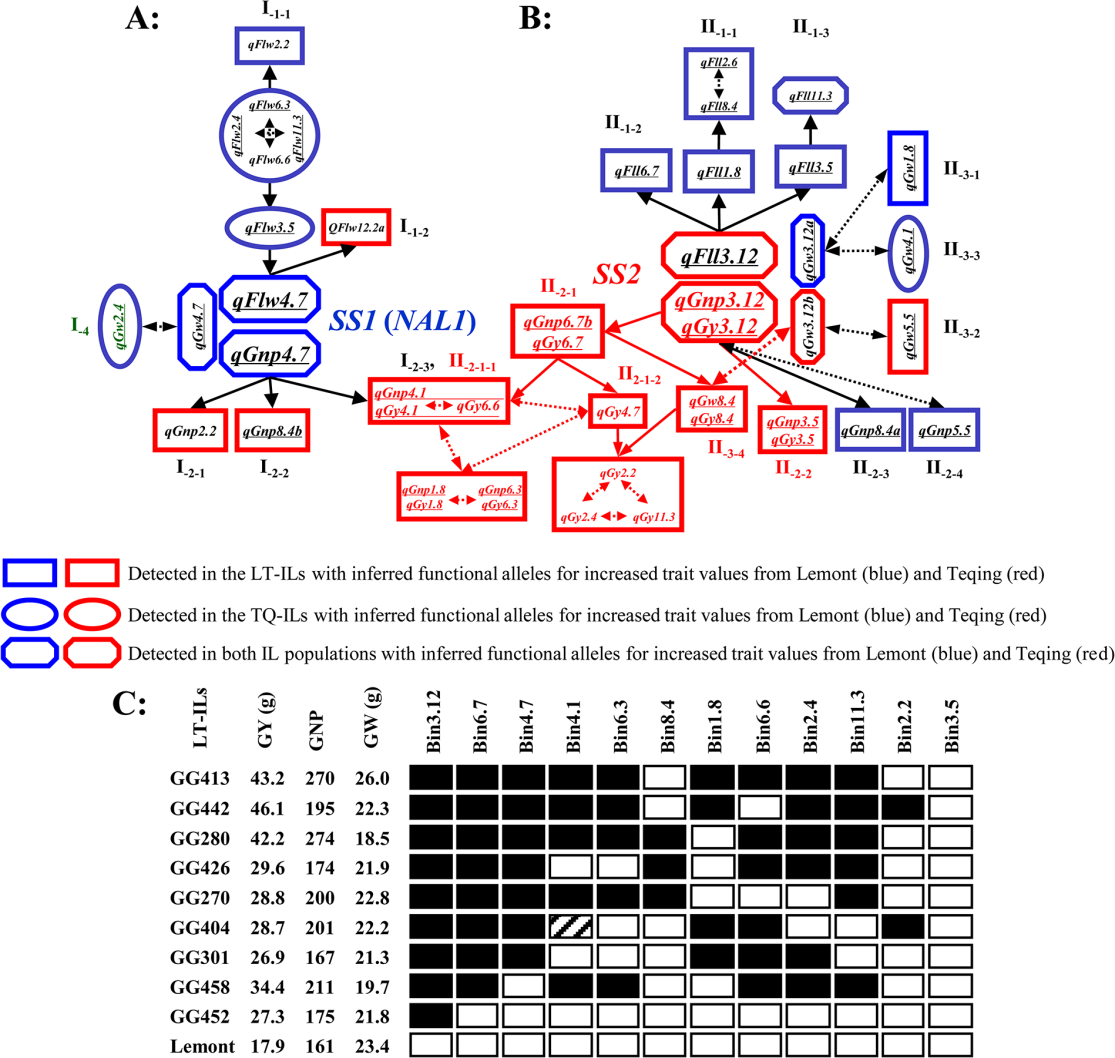
The putative genetic networks underlying source leaf traits (flag leaf width and length: FLW and FLL), sink size traits (grain number per panicle and 1000-grain weight: GNP and GW), and grain yield (GY) detected in the two reciprocal introgression populations of the LT/TQ cross (LT-ILs and TQ-ILs). **a:** The *SS1* (***qFlw4*.*7***, ***qGnp4*.*7*** and ***qGw4*.*7***) mediated pathways affecting FLL, GNP and GW; **b:** The *SS2* (***qFll3*.*12***, ***qGnp3*.*12*** and ***qGw3*.*12***) mediated pathways affecting FLL, GNP, GW and GY. The rectangular and oval shaped boxes represent the interacting QTL detected in the LT-ILs and TQ-ILs, respectively, while the hexagon shaped boxes represent the interacting QTL detected in both IL populations. Single arrowed solid lines each connecting two interacting QTL indicate an inferred one-way functional dependency (FD) between a upstream QTL and a downstream one of the interacting QTL pair, while double-arrowed dot lines each linking two interacting QTL indicate the mutual FD between the interacting QTL in the same pathway, predicted based on the pattern of the mean trait values of the 4 digenic genotypes [40]. The estimated pathway effects on the traits of each downstream QTL are shown in S7 Table. Underlined QTL were also detected as main-effect QTL (S6 Table). **c:** The graphical genotypes of 9 high yielding LT-ILs at bin 3.12 (*SS2*) and its 11 downstream loci and their mean phenotypic values for GNP, GY and GW, in which the black boxes are the homozygotes of the introgressed donor (TQ) alleles and the patched one was heterozygous.

When the patterns of the observed mean phenotypes of the digenic genotypes at each of the interacting QTL pairs were fitted to the expectations of the function-mutant model [40], two types of FD could be inferred between the interacting QTL, i.e. the one-way FD of a downstream one on its upstream one and the mutual FD between two loci in the same pathway. Furthermore, it could be inferred that the parental alleles at each locus of the interacting QTL pairs consisted of one function allele and a non-functional mutant allele. This information allowed us to construct putative genetic networks underlying the measured SS and yield traits (Fig 1).

#### The *SS1* mediated pathways

Fig 1A shows the *SS1* (bin 4.7) mediated genetic network consisting of 11 QTL affecting FLW and GNP with the LT alleles at *SS1* predicted to be the regulator(s) controlling 10 QTL of two major groups in the downstream, supported by 21 significant pairwise interactions (S7 Table). Group I-1 included *qFlw4*.*7* as the regulator and 7 FLW QTL in two downstream branches. Branch I_-1-1_ comprised 6 interacting QTL with *qFlw3*.*5* in the upstream, 4 interacting QTL (*qFlw6*.*3*, *qFlw6*.*6*, *qFlw2*.*4* and *qFlw11*.*3*) in the middle, and *qFlw2*.*2* in the downstream (interactions 1–16 in S7 Table). The LT alleles at all 6 loci of branch I_-1-1_ were inferred to be functional while the TQ alleles were non-functional mutations at these loci. Branch I_-1-2_ contained a single QTL *qFlw12*.*2a* in the downstream of ***qFlw4*.*7*** with the functional allele from TQ and estimated a pathway effect of 1.3 (1.8) mm for increased FLW in Beijing (Sanya). Group I-2 had 3 loci in the downstream of ***qGnp4*.*7*** affecting GNP only. Branch I_-2-1_ (*qGnp2*.*2*) had a pathway effect of 29 (17) for increased GNP in Beijing (Sanya) (interaction 18 in S7 Table). Branch I_-2-2_ (*qGnp8*.*4b*) had a pathway effect of 39 (34) for increased GNP in Beijing (Sanya) (interaction 20 in S7 Table). Branch I_-2-3_ had *qGnp4*.*1* in the downstream of *qGnp4*.*7* with an estimated pathway effect of 29 (57) for increased GNP in Beijing (Sanya) (interaction 27 in S7 Table). The functional alleles for all GNP QTL downstream of *SS1* were from TQ. In addition, *qGw4*.*7* at *SS1* interacted with *qGw2*.*4* in a complementary manner with the LT/LT genotype having 2.4 (2.5) g of heavier GW than the remaining 3 digenic genotypes in Beijing (Sanya) (interaction 34 in S7 Table), suggesting the LT alleles at both loci were functional.

#### The *SS2* mediated pathways

Fig 1B shows the *SS2* mediated genetic network with *qFll3*.*12*, *qGnp3*.*12* (*qGy3*.*12*) and *qGw3*.*12* as inferred regulator(s) and 20 QTL in three major downstream groups, supported by 60 highly significant pairwise interactions (S7 Table). Group II-1 consisted of *qFll3*.*12* as the regulator controlling 6 FLL QTL in 3 downstream braches (interactions 35–46 in S7 Table). Branch II_-1-1_ contained 3 interacting loci with *qFll1*.*8* in the upstream and 2 interacting loci (*qFll2*.*6* and *qFll8*.*4*) in the downstream with a pathway effect of 3.3 (2.2) cm for increased FLL in Beijing (Sanya). Branch II_-1-2_ contained *qFll6*.*7* in the downstream of *qFll3*.*12* with a pathway effect of 2.5 (2.4) cm for increased FLL in Beijing (Sanya). Branch II_-1-3_ consisted of 2 interacting QTL with *qFll3*.*5* in the upstream and *qFll11*.*3* in the downstream and a pathway effect of 3.0 (2.9) cm for increased FLL in Beijing (Sanya). The LT alleles at all 6 downstream loci were inferred to be functional, while the TQ alleles at these loci were non-functional mutants.

Group II-2 was the most important one with *qGnp3*.*12* (*qGy3*.*12*) as the regulator controlling 10 downstream loci affecting GNP and GY (Fig 1B), supported by 45 highly significant interactions (S8 Table). Branch II_-2-1_ had *qGnp6*.*7b* (*qGy6*.*7*) in the upstream and 8 downstream loci affecting GNP and/or GY in 2 sub-branches. Sub-branch II_-2-1-1_ involved 4 interacting loci, *qGnp4*.*1* (*qGy4*.*1*), *qGy6*.*6*, *qGnp1*.*8* (*qGy1*.*8*) and *qGnp6*.*3* (*qGy6*.*3*) with pathway effects of 32 (44) for increased GNP, and 8.5 g (13.4 g) for increased GY in Beijing (Sanya). Sub-branch II_-2-1-2_ involved 4 interacting loci affecting GY with *qGy4*.*7* in the upstream and 3 loci (*qGy2*.*2*, *qGy2*.*4* and *qGy11*.*3*) in the downstream (Fig 1B and S8 Table). II_-2-2_ (*qGnp3*.*5* and *qGy3*.*5*) was a single QTL downstream of *SS2* with estimated pathway effects of 40 (61) for increased GNP, and 14.7 g (6.9 g) for increased GY in Beijing (Sanya). The TQ alleles at all above loci were predicted to be functional. In particular, Fig 1C suggests that co-introgression of the TQ alleles at *SS2* (*qGnp3*.*12/qGy3*.*12/qGw3*.*12b*) and varied combinations of its 11 downstream loci affecting GNP, GY and GW QTL into 8 LT ILs resulted in an average increased GY by 95.5% (Fig 1C). Branches II_-2-3_ (*qGnp8*.*4a*) and II_-2-4_ (*qGnp5*.*5*) affected GNP only with the estimated pathway effects of 31 (25) and 31 (40) for increased GNP in Beijing (Sanya) with functional alleles at *qGnp8*.*4a* and *qGnp5*.*5* from LT.

Group II-3 consisted of 4 pairs of interacting loci affecting GW. Of these, *qGw3*.*12a* was interacting with *qGw1*.*8* and *qGw4*.*1* in a complementary manner. The functional alleles at the three loci were all from LT for increased GW. The remaining two interactions occurred between *qGw3*.*12b* and *qGw5*.*5* or *qGw8*.*4*. The TQ alleles at all three loci were predicted to be functional for increased GW. The functional genotype of the *qGw3*.*12b* and *qGw8*.*4* pair was associated with significantly increased GY (S7 Table and Fig 1B).

### 3.2. Molecular cloning of *SS1*

To verify the genetic networks and validity of the theoretical model, the following experiments were conducted to clone *SS1*:

#### Fine mapping of *SS1*

**Fig 2:**
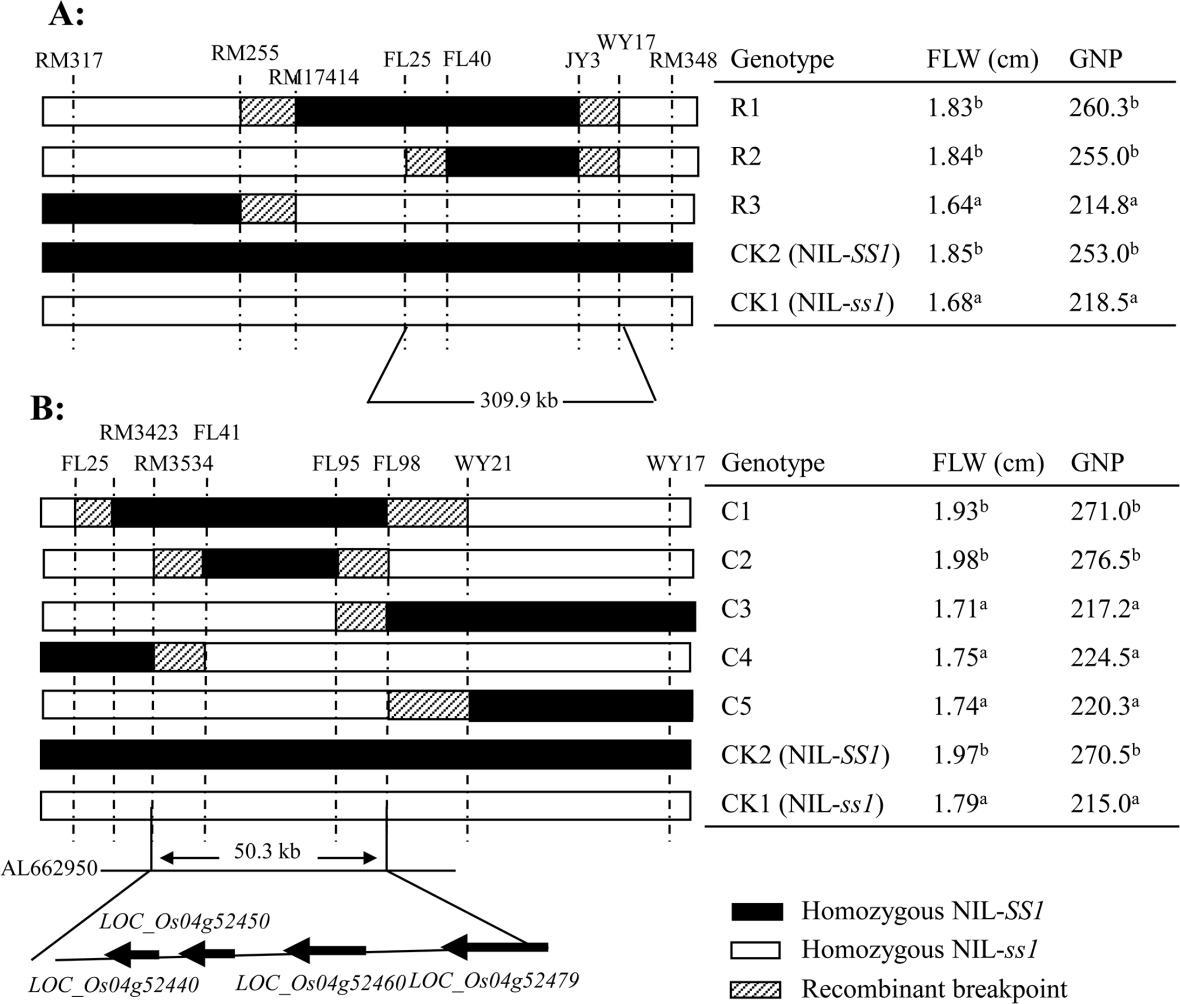
High-resolution mapping of the *SS1* locus. Darkly shaded, open and lightly shaded rectangles stand for the homozygous LT genotype, homozygous TQ genotype, and the marker intervals containing recombination breakpoint. **a:** The *SS1* location in the 309.9 kb region flanked by FL25 and WY17 based on the marker genotypes of 6,000 BC_4_F_3_ individuals derived from the cross between TQ and NIL-*SS1*. **b:** The *SS1 locus* in a 50.3 kb region flanked by RM3534 and FL98 narrowed down by genotypes of 12,000 BC_4_F_4_ individuals, where four candidate genes were predicted based on the annotated rice genome database (http://rice.plantbiology.msu.edu/cgi-bin/gbrowse, accessed on 2 Dec, 2014). The superscript letters (a and b) on the left panel indicate significant differences in the traits of the recombinants as compared with those of the parental controls at a level of *P* ≤ 0.01.

Genotyping of 6,000 BC_4_F_3_ individuals derived from six BC_4_F_2_ plants heterozygous only at *SS1* with 6 markers identified 25 informative recombinants of three genotypes within this region (Fig 2A). Multiple comparisons of the homozygous recombinant BC_4_F_4_ lines for FLW and GNP with the non-recombinant controls placed *SS1* in a 309.9 kb region between FL25 and WY17 (Fig 2A). Further fine mapping using 12,000 BC_4_F_4_ plants using six new markers between FL25 and WY17 identified 28 informative recombinants and five genotypic classes in the target region, narrowing *SS1* down to a 50.3-kb region between RM3534 and FL98 in a single bacterial artificial chromosome clone, AL662950 (http://www.gramene.org/Oryza_sativa/, accessed on 2 Dec, 2014) (Fig 2B). This region contains four predicted genes (*LOC_Os04g52440*, *LOC_Os04g52450*, *LOC_Os04g52460* and *LOC_Os04g52479*) (http://rice.plantbiology.msu.edu/cgi-bin/gbrowse, accessed on 2 Dec, 2014). *LOC_Os04g52440* and *LOC_Os04g52450* each encodes a 4-aminobutanoate-pyruvate aminotransferase protein with 70% homology. *LOC_Os04g52460* is a retrotransposon. *LOC_Os04g52479* is previously reported as *NARROW LEAF1* (*NAL1*), encoding a plant-specific protein involved in the auxin polar transport and acted in the upstream of the auxin/IAA signaling pathways [46, 47]. Three naturally occurred alleles at *NAL1* were also cloned as major QTL, *SPIKE* [48], *GPS* [49] and *LSCHL4* [50] each with pleiotropic effects on FLW, SNP, roots, photosynthesis rate, yield, etc.

#### DNA sequence and protein structure variants of *SS1*

**Fig 3:**
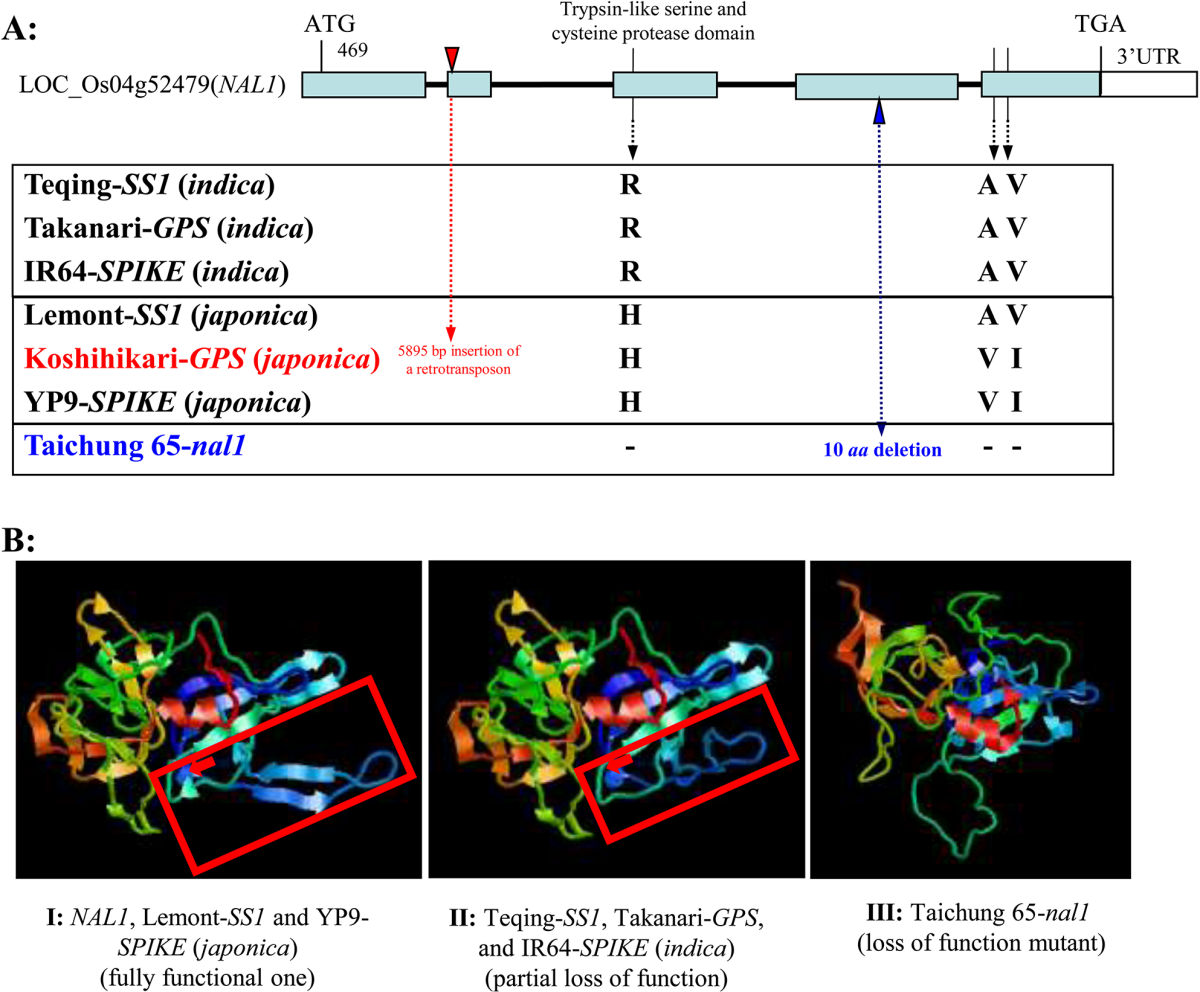
Comparison of protein mutation sites of different alleles at *SS1* (*NAL1*). **a:** Analyses of the predicted amino acid sequences of *SS1*, *GPS* [49], *SPIKE* [48] and *LSCHL4* [50] revealed three haplotypes of functional alleles at *NAL1*: the *indica* allele, R (arginine)–A (alanine)–V (valine); the *japonica* allele, H (histidine)–V–I (isoleucine); and a hybrid allele, H–A–V; plus two loss of function mutations: the Koshihikari one with a 5895 bp insertion of a retrotransposon in the second exon and the Taichung 65 mutation with a 10 aa deletion in the fourth exon. The key amino acid substitutions between TQ and its NIL-*SS1* is the R-to-H substitution locates in the trypsin-like serine and cysteine protease domain of the *NAL1* protein. **b:** Comparison of the predicted 3-D trypsin-like serine and cysteine protease domain structures of different alleles at *SS1* (*NAL1*), using the SWISS-MODEL (a fully automated protein structure homology-modeling server, http://swissmodel.expasy.org/, accessed on 2 Dec, 2014). **I:** *NAL1*, *SS1*-Lemont and *SPIKE*-YP9 (*japonica*), based on template [2ouaB] (1.85 Å), e-value: 1.1E6; **II:** *SS1*-Teqing, *GPS*-Takanari and *SPIKE*-IR64 (*indica*), based on template [2ouaB] (1.85 Å), 9.6E7; **III:** the Taichung65 *nal1*-mutant allele, based on template [2qa9E] (1.18 Å), evalue: 3.5E7. The red arrows indicate the R-to-H substitution within the trypsin-like serine and cysteine protease domain, and the predicted 3-D domain structure changes are shown by the red boxes. The predicted 3-D protein structure change in the *nal1* mutant resulting from the 10aa deletion in the fourth exon is enormous, which apparently disrupts most (if not all) functions of the protein.

S3 Fig shows the complete DNA sequences containing the coding regions and 1440 bp upstream the transcription starting sites of the three *SS1* candidate genes in the NIL*-SS1*, LT and TQ. For *LOC_Os04g52440*, the NIL*-SS1*, LT and TQ have the completely identical sequence in the coding region, but the NIL*-SS1* (LT) allele has a 17-bp deletion, 2 nucleotide insertions and 14 SNPs upstream the transcription start site when compared with the TQ allele. In *LOC_Os04g52450*, many sequence variants were detected between the NIL*-SS1* (LT) and TQ alleles, including three nucleotide substitutions in the coding region that resulted in amino acid variations, plus a 34-bp deletion, 38 SNPs and 9 small Indels/Deletions in the promoter region of the gene (S4 Fig). For *LOC_Os04g52479* (*NAL1*), only a single SNP at site 160 upstream the start codon and one nucleotide change which results in the substitution of a histidine (CAT) in the trypsin-like serine and cysteine protease domain of the wide leaf group (NIL*-SS1* and LT) by an arginine (CGT) in the narrow leaf group (TQ) (Fig 3A and S5 Fig). This single amino acid substitution results in a clear change in the predicted 3-D structure in the trypsin-like serine and cysteine protease domain of the *NAL1* protein between the TQ-*SS1* and LT-*SS1* alleles and a predicted partial loss of function of the *NAL1* protein in TQ (Fig 3B). When the allelic diversity at this site in 144 rice accessions from 16 countries was examined (S9 Table), the overall frequencies of histidine and arginine at this site are 0.424 and 0.576, respectively. However, the former is predominant in the 71 *japonica* accessions with a frequency of 0.662 (0.843 in the 51 modern *japonica* varieties), while the latter was predominant in the 73 *indica* accessions with a frequency of 0.808 (100% in 19 *indica* landraces). The difference in FLW between the CAT (histidine) and CGT (arginine) alleles was insignificant, though they differed significantly for FLW in *indica* (*P* = 0.022) and *japanica* varieties (*P* = 0.026), respectively. The CAT allele (FLW = 1.77cm) had wider FLW than the CGT allele (FLW = 1.51cm) in *indica* varieties. However, the CAT allele (FLW = 1.37cm) had smaller FLW than the CGT allele (FLW = 1.62cm) in *japonaica* varieties.

#### Gene expression

At the panicle initiation stage, *LOC_Os04g52440* expressed at a high level in leaf sheaths and panicles of both the NIL*-SS1* and TQ, while NIL-*SS1* showed a significantly higher expression rate than TQ in flag leaves, leaf sheaths and internodes (S6A Fig). *LOC_Os04g52450* expressed at a high level in all tissues with significantly higher expression rates in flag leaves, leaf sheaths and internodes in the NIL-*SS1* than in TQ (S6B Fig). *LOC_Os4g52479* (*NAL1*) expressed in all tissues but more strongly in nodes and root/stem conjunctions (S6C Fig). However, differences between the NIL*-SS1* and TQ in the expression level of *NAL1* were insignificant in all tissues, indicating that the SNP in the promoter region between the NIL-*SS1* and TQ did not affect the expression of *NAL1*.

#### Phenotypic differences between the NIL-*SS1* and TQ

The NIL-*SS1* and TQ were similar in plant type and grain size (S7A Fig), but the former had significantly wider FLW and more GNP and filled grains per panicle than TQ in Hangzhou, Beijing and Sanya (S10 Table and S8A Fig). The difference in grain yield per plot between the NIL-*SS1* and TQ was insignificant, though the *SS1*-NIL had consistently higher yield than TQ by 0.4 (3.6%) and 0.5 (3.8%) and 0.3 (3.2%) kg per plot in Hangzhou, Beijing and Sanya, respectively. The NIL*-SS1* plants were more vigorous than TQ (S7B Fig). Also, the NIL-*SS1* showed consistently reduced photosynthesis rates from the booting stage to late grain filling stage (S11 Table and S8B Fig) in Beijing for 2010 and 2011 when compared with TQ, except that it had a significantly higher photosynthesis rate than TQ at grain filling stage in 2010. No differences in photosynthesis related traits were observed between the NIL-*SS1* and TQ in 2012 (S11 Table). Compared to TQ, the NIL-*SS1* showed consistently reduced rates of accumulation and translocation of dry matter (assimilates) in stems and sheaths (S8D Fig), though no significant difference was observed between TQ and the NIL-*SS1* for the rate of dry matter accumulation in panicles (S8C Fig).

## Discussion

Increasing yield has been the most important target in rice breeding worldwide. Both TQ and LT were commercial cultivars, but TQ has much higher yields and wider adaptability than LT. Tremendous efforts have been taken to dissect the genetic basis of yield using different populations derived from the LT/TQ cross, resulting in identification of large numbers of QTL and complex epistasis for the huge yield difference between the parents [9–12, 14, 15, 38]. Our experimental design of using the reciprocal IL populations plus phenotyping in the long-day summer crop season in Beijing and short-day winter nursery in Sanya provided a unique opportunity to verify all identified QTL in both parental GBs. Thus, the discovered genetic networks underlying SS and yield traits and the cloning of *SS1* provided direct evidence and new insights into the genetic and molecular basis of SS and yield traits in rice.

### The genetic basis of the SS and yield traits in rice

Our results provided overwhelming evidence for the importance of epistasis in determining SS and yield traits in rice. In this study, 85% of the identified M-QTL and 97% of the epistatic interactions were detectable only in one of the parental GBs. Historically, GB effects of QTL have been reported in rice [15, 37, 51–54], tomato [55, 56], drosophila [57] and humans [58], and interpreted as evidence for the presence of epistasis. However, it remains unclear how epistasis causes the GB effects of M-QTL as the classical quantitative genetics theory [37, 39] predicts that M-QTL should be detectable in both parental GBs. According to the new function-mutation model [40], a gene acting in signaling pathways affecting a complex trait can be detected as M-QTL in both reciprocal ILs only under two scenarios: (1) it is a regulatory gene controlling multiple downstream pathways affecting the same trait and (2) it is the only locus in one of the downstream pathways segregating in the parents. *SS1* (*qFlw4*.*7*/*qGnp4*.*7*) and *SS2* (*qFll3*.*12/qGnp3*.*12/qGw3*.*12b*) presented the former case, while the other four GB “independent” loci (*qFlw6*.*5*, *qFlw12*.*4*, *qFll12*.*4* and *qGw7*.*5*) fell into the latter. The theory also predicts that when *n* loci (*n* ≥ 2) acting in a downstream pathway affecting a complex trait are segregating between the parents, P_1_ and P_2_, with the functional alleles at *k* (0 *≤ k ≤ n*) loci in P_1_ and those at *n—k* loci in P_2_, then the *k* loci would be detectable only in the P_2_ (mutant) GB, as a result of genetic complementarity. Thus, one would expect that the *k* loci would be detectable (having main and/or epistatic effect) in the P_2_ GB only if they are co-introgressed into P_2_ with the functional allele(s) at their regulator(s), and so for the *n—k* loci in the P_1_ GB. Consistent with the theoretical prediction, the five FLW QTL downstream of *SS1* were detectable only in the TQ-ILs when they were co-introgressed into the TQ GB with the functional (LT) allele at *SS1* (Fig 1A and S8 Table). The same was true for most GNP (GY) QTL downstream of *SS2* (*qGy3*.*12* or *qGnp3*.*12*) (Fig 1B). However, most FLL QTL and 2 GNP QTL (*qGnp5*.*5* and *qGnp8*.*4a*) downstream of *SS2* were detected in the LT-ILs by genetic knock-out (introgression of the mutant TQ alleles into the LT GB). This result suggested that those FLW loci downstream of *SS1* and those GNP (GY) loci downstream of *SS2* were somewhat “redundant” in their functions such that introgression of the mutant alleles at single downstream loci did not necessarily have phenotypic effects. Otherwise, those GY QTL would have been detected as M-QTL in the TQ-ILs. Curiously, one is wondering why TQ has the functional alleles at so many “redundant” loci affecting GY and fitness (GNP) traits and if this contributes to its high yield potential and yield stability, which remains an important question to be addressed in future.

### The nature of loci and allelic diversity underlying SS and yield traits in rice

Consistent with the theoretical prediction [40], most loci affecting rice SS and yield traits identified in this study were functioning in the downstream pathways and the presence of a functional allele and a non-functional mutant was predicted as the nature of allelic diversity at 46 loci involved in epistasis (S7 Table). Further, the mutant alleles at 87% of the loci affecting fitness (GNP) and productivity (GY) were from LT, indicating that the LT (*japonica*) genome has a much higher genetic load than the TQ (*indica*) genome. This was consistent with our previous mapping results [59]. Interestingly, we noted that the predicted functional alleles at all epistatic loci were associated with increased values of SS and yield traits. This implies that increased SS capacity and productivity in rice are primarily controlled by positively regulated pathways. Then, one would predict that it is much easier to break a functional multi-locus high yielding genotype than to restore it in randomly segregating breeding populations, which is consistent with empirical observations in plant breeding.

### The molecular basis and functionality of the *SS1*-mediated pathways

Our QTL cloning results plus combined evidence from three recent reports indicate that *SS1* (*qFlw4*.*7*/*qGnp4*.*7*) are the same QTL as *SPIKE* [48], *GPS* [49] or *LSCHL4* [50], representing allelic differences at *NAL1*, a gene encoding a trypsin-like serine/cysteine protease involved in the auxin transport by regulating many downstream genes in the auxin/IAA signaling pathways [46, 47]. However, the phenotypic effects of different alleles at *NAL1* in the three QTL cloning studies appear to be ‘inconsistent’. At the sequence level, the alleles at *NAL1* in the parents of the 4 independent studies can produce five predicted *NAL1* protein variants (Fig 3A). These include two functional *japonica* (LT-*SS1* and YP9-*SPIKE* or Nipponbare-*LSCHL4*) alleles, one naturally occurred *indica* variant (TQ-*SS1*, IR64-*SPIKE*, Takanari-*GPS* and 9311-*LSCHL4*) from the substitution of a histidine in the *japonicas* by an arginine in the *indicas*, a loss of function *japonica* (Koshihikari-*GPS*) mutation with the insertion of a retrotransposon in the second exon, and an induced loss of function mutation (*nal1*) from a 10-*aa* deletion in the fourth exon of *NAL1* in Taichung 65. Interestingly, while the phenotypic effects of the functional Takanari-*GPS* (*indica*) allele causing narrower leaves and increased photosynthesis rate were detectable in both the parental GBs, its effect for increased yield was detectable only in the Takanari (*indica*) GB by ‘genetic knockout’, but not in the Koshihikari (*japonica*) GB by ‘gain of function’ [49]. In contrast, the YP9-*SPIKE* or Nipponbare-*LSCHL4* (*japonica*) allele was associated with wider leaves and increased yield across several *indica* GBs, when compared with the *SPIKE*-IR64 or *LSCHL4*-9311 (*indica*) allele [48, 50]. In this study, the LT-*SS1* (*japonica*) allele was predicted to be functional and associated with wider leaves and more GNP, which were consistent with the other two reports. However, its effects on increased yield and reduced photosynthesis rate were inconsistent in the NIL-*SS1*. Taking all the data together, we came up with a clearer picture of the *SS1*-mediated pathways discovered in this study. First, the *SS1*-mediated pathways affecting SS and yield traits are inferred to be part of the auxin mediated pathways in which different alleles at *NAL1* play important regulatory roles by qualitative (the *nal1* mutant) and/or quantitative control of the efficiency and tissue specificity of IAA transport from the apical meristem to other plant tissues (leaves, stems, roots and panicles, etc). Second, the substitution of a histidine in the *japonicas* by an arginine in the *indicas* in the trypsin-like serine and cysteine protease domain of the *NAL1* protein resulted in the predicted structural change and partial loss of function of this protein *in the indicas* (Fig 3B). This protein structural change apparently causes its inability to interact or to be recognized by other proteins [46] in the IAA signal transduction and failure to regulate downstream loci which positively control GNP, leaf width and area, and roots of rice. Third, the *japonica* haplotype (H-V-I) and *indica* (R-A-V) one at *NAL1* involve 3 amino acid substitutions (Fig 3A) and are inferred to be ancient since the *indica*-*japonica* differentiation. The LT-*SS1* haplotype was a hybrid apparently resulting from its *indica*-*japonica* origin. Further, we note that wider leaves, large panicles (more secondary branches), fewer panicles, and strong/deep roots are typical characteristics of most *japonica* landraces, and the opposite is true for most *indica* landraces. This suggests that this allelic differentiation at *NAL1* may have contributed to the well-known *indica*-*japonica* subspecific differentiation and their respective adaptations to the warmer, more humid and cloudy environments in the tropics by *indicas* and the colder and drier areas of high latitude or elevation by *japonicas*. Forth, the phenotypic effects of different *NAL1* alleles on rice productivity and other traits are predicted to depend on the presence and number of the functional alleles at its downstream loci, which would vary considerably in different rice GBs, as clearly demonstrated in this study (S6 and S7 Tables). This was not surprising because its main effects actually represented the cumulated phenotypic effects of many different functioning downstream pathways in the GB. Thus, like the well-known story of the Green Revolution gene, *sd1* [20], our results provides another piece of direct evidence validating the function-mutation model of the molecular quantitative genetics theory [40] and its power in dissecting complex traits controlled by signaling pathways.

### The *SS2*-mediated pathways

The *SS2*-mediated pathways resembled very much the *SS1*-mediated pathways. Like *SS1*, *SS2* was detected as a major M-QTL cluster (*qFll3*.*12*/*qGnp3*.*12/qGy3*.*12/qGw3*.*12a*) and a key regulator with pleiotropic effects on FLL, GNP, GY and GW by regulating multiple downstream loci (Fig 1A and 1B). However, the phenotypic effects of the *SS2*-mediated pathways on rice productivity were much stronger, primarily through the functional TQ (*indica*) alleles at many downstream pathways regulated by the functional TQ (*indica*) alleles at *SS2*. This was consistent with our expectation that alleles for increased productivity at most loci were from TQ. Genetically, the *SS1*- and *SS2*-mediated pathways appeared to be largely independent from each other. The only overlap was the interacting QTL pair, *qGnp4*.*1*/*qGy4*.*1*−*qGy6*.*6*, and their downstream ones, *qGnp1*.*8*/*qGy1*.*8−qGnp6*.*3*/*qGy6*.*3* detected in the downstream of both *SS1* and *SS2*. Interestingly, an epistatic GY QTL, *qGy4*.*7* at *SS1* was interacting with several loci in the downstream of *SS2*, at which the TQ *SS1* (*qGy4*.*7*) allele was associated for increased GY (Fig 1A and 1B). While this appeared to be consistent with the positive effect of the *GPS*-Takanari allele for increased yield [49], the possibility that *qGy4*.*7* was a different gene tightly linked to *SS1* could not be ruled out because sequence diversities at many loci, including *LOC_Os04g52440* and *LOC_Os04g52450*, are known to exist in the *SS1* region. Now, we are in the progress to clone *SS2*, which is expected to provide important evidence on the nature of the *SS2* mediated pathways.

Here, we realize that there is a huge knowledge gap to link the allelic diversity at *SS1 or SS2* to their phenotypic effects and many important questions remain to be elucidated in future studies. For example, what are those downstream genes and pathways mediated by *SS1* and *SS2*, and how are they regulated? What are those loci and pathways downstream of *SS1* and *SS2*, and how do they determine rice SS traits and productivity, etc? These questions pose a huge challenge to rice scientists since it would be very difficult to clone those downstream QTL by the map-based cloning approach because of their relatively small effects and complex epistasis involved. Fortunately, with the genetic information on these downstream loci, specific genetic stocks (NILs and ILs of different allelic combinations at the downstream loci in different GBs), available sequences of the parental genomes, different omic technologies are now being used to address these questions. We also realize that the SS traits measured in this study included only morphological components, but not biochemical components, though the association of the Takanari-*GPS* (*indica*) allele at *NAL1* with increased photosynthesis rate already suggests such possibilities. Also, the SS relationship in rice and other cereals is dynamic, which can be better addressed using systems mapping [60] in future.

### Implications in rice improvement

Our results have important implications for further improving rice yield potential and stability through strategies of “molecular ideotype breeding” and “molecular population improvement”. Currently, three major ideotypes have been proposed and practiced in breeding to break the yield plateaus of semidwarf rice inbred and hybrid cultivars, including the large panicle (LP) type with large SS traits with fewer panicles per unit area [61, 62], the numerous panicle (NP) type with small SS traits and more panicles per unit area [62] and the intermediate panicle (IP) type [63]. In practice, the LP type cultivars tend to show high yield potential under high-input (fertilization) conditions, while the NP type varieties tend to perform better under low-input conditions. The IP type varieties tend to show a much broader adaptability and higher yield potential across both high- and low-input conditions. Lines with extreme large panicle or leaf size are not normally high yielding due to disharmony between the SS traits [6, 64]. Our results suggest that varieties with different SS trait combinations can be developed by accurately manipulating different downstream loci to achieve enhanced and balanced SS relationships to maximize yield performances under various scenarios of input. Obviously, this can not be achieved by transferring single or a few major QTL because of the pleiotropic effects of single regulatory genes such as *SS1* and *SS2* are expected to vary considerably depending on the functionality of its downstream pathways in different GBs. Also, one can perceive that the pleiotropic effects of a downstream pathway is physiological, which can not be broken by recombination. Thus, the long-term breeding strategy for further improving the yield potential and stability of these high yielding Chinese *indica* lines requires systematic exploitation of more and novel functional alleles from exotic germplasm [65] and efficient transfer and pyramiding of these favorable alleles in the elite rice GBs using the molecular recurrent selection strategy [66].

## Supporting Information

S1 Fig Chromosomal locations of 69 QTL affecting flag leaf length (FLL), flag leaf width (FLW), grain number per panicle (GNP), 1000-grain weight (GW) and grain yield per plant (GY) detected in the two sets of reciprocal introgression lines (ILs), derived from the Lemont (LT)/Teqing (TQ) cross. Red and blue lined boxes are QTL at which the alleles for increased trait values are from TQ and LT, respectively. Unfilled and patch-filled boxes are QTL detected in the Sanya and Beijing environments. Filled (black) boxes are QTL detected in both environments. Underlined QTL are those previously detected in the related mapping populations derived from the same parents [10, 14, 15, 38]. The detailed information for the 478 SSR and SNP markers is shown in S1 Table.
(PPTX)

S2 Fig The process of the material development for fine mapping *SS1*. (PPTX)

S3 Fig Comparison of *LOC_Os04g52440* DNA sequences for the NIL-*SS1* and its parents. The gray regions indicate the coding region. The red bars indicate the substitutions and deletions between LT, NIL (NIL-*SS1*) and TQ. Asterisks indicate complete homology; semicolons indicate substitution of DNA sequences; and spaces indicate a complete lack of homology. Integers on the right indicate the cumulative number of nucleotide.
(DOCX)

S4 Fig Comparison of *LOC_Os04g52450* DNA sequences for the NIL-*SS1* and parents. The gray regions indicate the coding region. The red bars indicate the substitutions and deletions between LT, NIL (NIL-*SS1*) and TQ. Asterisks indicate complete homology; semicolons indicate substitution of DNA sequences; and spaces indicate complete lack of homology. Integers on the right indicate the cumulative number of nucleotides in the coding region.
(DOCX)

S5 Fig Comparison of the *LOC_Os04g52479* DNA sequences for NIL-*SS1* and its parents. The gray regions indicate the coding region. The green regions indicate the trypsin-like serine and cysteine protease domain of *NAL1* mutant. Underlined sequence indicates the deletion corresponding to the 10 amino acid. The red bars indicate the substitutions and deletions between LT, NIL-*SS1* and TQ. Asterisks indicate complete homology; semicolons indicate substitution of DNA sequences; and spaces indicate complete lack of homology. Integers on the right indicate the cumulative number of nucleotide in the coding region [48].
(DOCX)

S6 Fig Relative expressions of the three candidate genes in the *SS1* region in TQ and its NIL-*SS1* in panicles, flag leaves, leaf sheaths, nodes, internodes, stem junctions and roots at the panicle initiation stage, A: *LOC_Os04g52440*, B: *LOC_Os04g52450* and C: *LOC_Os04g52479*. (PPTX)

S7 Fig Characterization of plant type, sink and source related traits of NIL-*SS1* and TQ in Beijing. (A) Plant morphologies (left), panicle type (middle), and flag leaves (right); (B) Population performances of TQ (left) and NIL-*SS1* (right) in 2012 summer season in Beijing.
(PPTX)

S8 Fig Phenotypic differences between TQ and its NIL-*SS1* in phenotypic and physiological traits, A: Flag leaf width and area; B: Photosynthesis rate; C: Accumulation of dry matter in panicles; D: Rates of dry matter translocation in stems and sheaths. (PPTX)

S1 Table The SSR (RM) and SNP (Os) marker and bin information used for the linkage map construction and QTL analyses in the reciprocal sets of introgression populations of the LT/TQ cross. (DOC)

S2 Table InDel and CAPS markers designed for fine mapping of the *SS1*. (DOC)

S3 Table Primers used for sequencing analysis and quantitative real-time PCR (qRT-PCR) of *SS1*. (DOC)

S4 Table Summarized statistics of the reciprocal introgression lines (ILs) and their parents, Lemont (LT) and Teqing (TQ) for five source leaf and yield traits evaluated in Beijing (BJ) and Sanya (SY). (DOC)

S5 Table ANOVA results of the reciprocal introgression lines (ILs) of Teqing (TQ) and Lemont (LT) for five source leaf and yield traits evaluated in Beijing (BJ) and Sanya (SY). (DOC)

S6 Table Main-effect QTL affecting SS-related traits flag leaf width (FLW, in mm) and length (FLL, in cm), grain number per panicle (GNP), 1000 grain weight (GW, in g) and grain yield (GY, in g) detected in the LT/TQ reciprocal IL populations and two environments (Beijing–BJ and Sanya–SY). (DOC)

S7 Table Eighty-seven pairwise epistatic interactions between 46 QTL and estimated pathway effects “a” on flag leaf width (FLW, in mm) and length (FLL, in cm), grain number per panicle (GNP), grain yield per plant (GY, in g), and 1000-grain weight (GW, in g) detected in the Lemont (LT-ILs) and Teqing (TQ-ILs) reciprocal introgression lines in Beijing and Sanya. (DOC)

S8 Table Comparison of 27 main-effect QTL and 46 epistatic QTL for different source-sink (SS) and yield traits detected in the reciprocal introgression populations of Lemont (LT) and Teqing (TQ) in Bejing and Sanya. (DOC)

S9 Table Allelic diversity of 144 rice accessions at the position of 698 bp in the coding region of *LOC_Os04g52479* (*NAL1*) gene. (DOC)

S10 Table Phenotypic differences between Teqing (TQ) and its *SS1* near isogenic line (NIL-*SS1*) and Lemont (donor, LT) for SS and yield traits in Hangzhou, Beijing and Sanya. (DOC)

S11 Table Flag leaf net photosynthetic rate *(P*_*n*_, μmol CO_2_ m^-2^s^-1^), stomatal conductance (*g*_*s*_, mol H_2_Om^-2^s^-1^), intercellular CO_2_ concentration (*C*_*i*_, μmol CO^-2^mol^-1^), transpiration rate (*T*_*r*_, mmol H_2_Om^-2^s^-1^), and specific leaf weight (SLW, mg cm^-2^) of leaf area of the two parents (Teqing and Lemont) and NIL*-SS1* evaluated under the irrigated condition in Beijing. (DOC)
