# Supplementary material for: SS1 (NAL1)- and SS2-Mediated Genetic Networks Underlying Source-Sink and Yield Traits in Rice (Oryza sativa L.)
Source: PLoS One. 2015 Jul 10;10(7):e0132060. doi: 10.1371/journal.pone.0132060 (PMC4498882; doi:10.1371/journal.pone.0132060)
Supplement: S1 Fig — Red and blue lined boxes are QTL at which the alleles for increased trait values are from TQ and LT, respectively. Unfilled and patch-filled boxes are QTL detected in the Sanya and Beijing environments. Filled (black) boxes are QTL detected in both environments. Underlined QTL are those previously detected in the related mapping populations derived from the same parents [10, 14, 15, 38]. The detailed information for the 478 SSR and SNP markers is shown in S1 Table. (PPTX) [file pone.0132060.s001.pptx]

## Slide 1
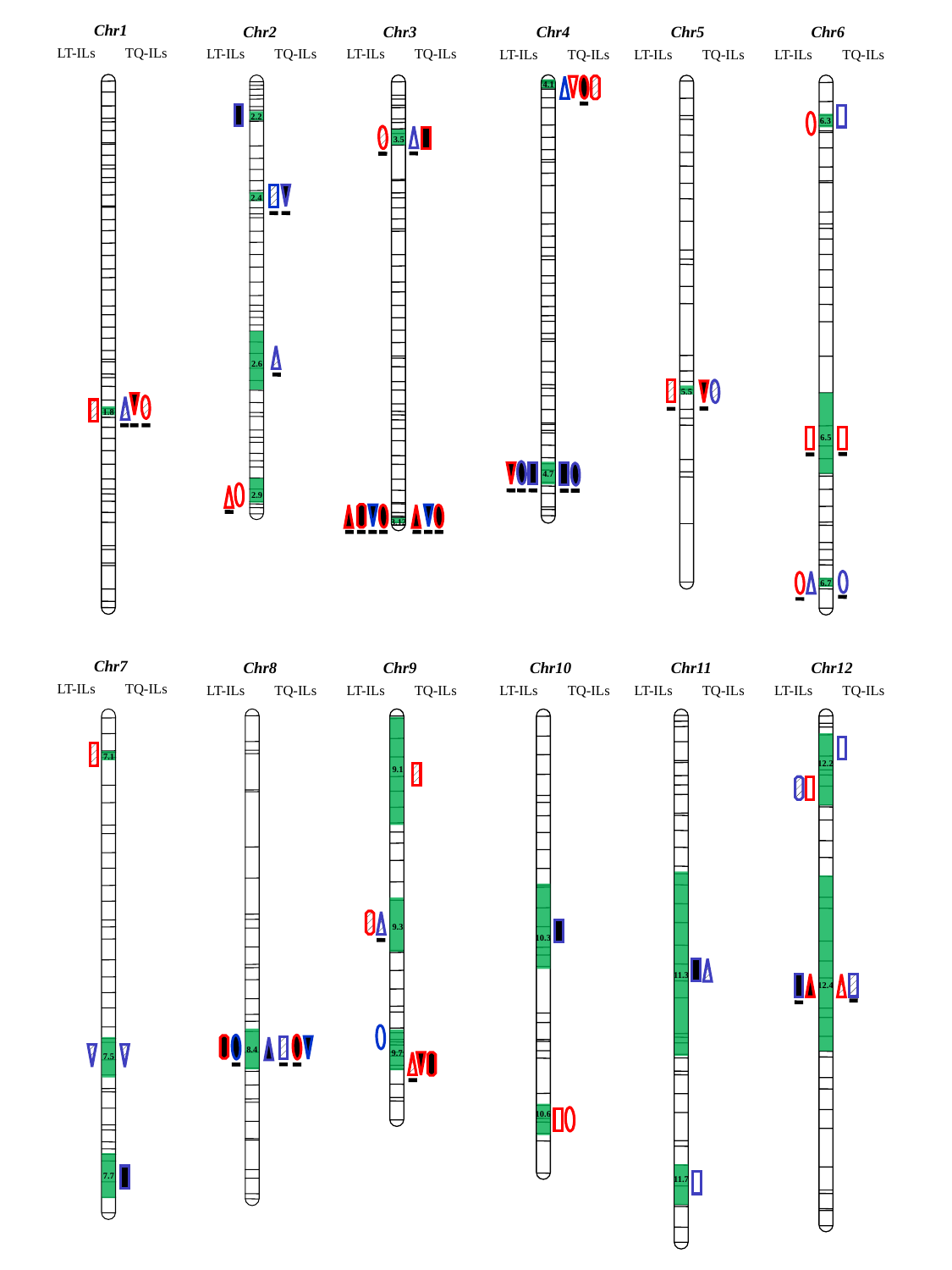

Chr1
 Chr2
 Chr3
 Chr5
 Chr6
 Chr4
LT-ILs
TQ-ILs
LT-ILs
TQ-ILs
LT-ILs
TQ-ILs
LT-ILs
TQ-ILs
LT-ILs
TQ-ILs
LT-ILs
TQ-ILs
4.1
4.7
3.5
3.12
2.2
6.3
2.4
2.6
5.5
1.8
6.5
2.9
6.7
 Chr7
 Chr8
 Chr9
 Chr11
 Chr12
 Chr10
LT-ILs
TQ-ILs
LT-ILs
TQ-ILs
LT-ILs
TQ-ILs
LT-ILs
TQ-ILs
LT-ILs
TQ-ILs
LT-ILs
TQ-ILs
7.1
7.5
7.7
12.2
9.1
9.3
10.3
11.3
12.4
8.4
9.7
10.6
11.7

## Slide 2
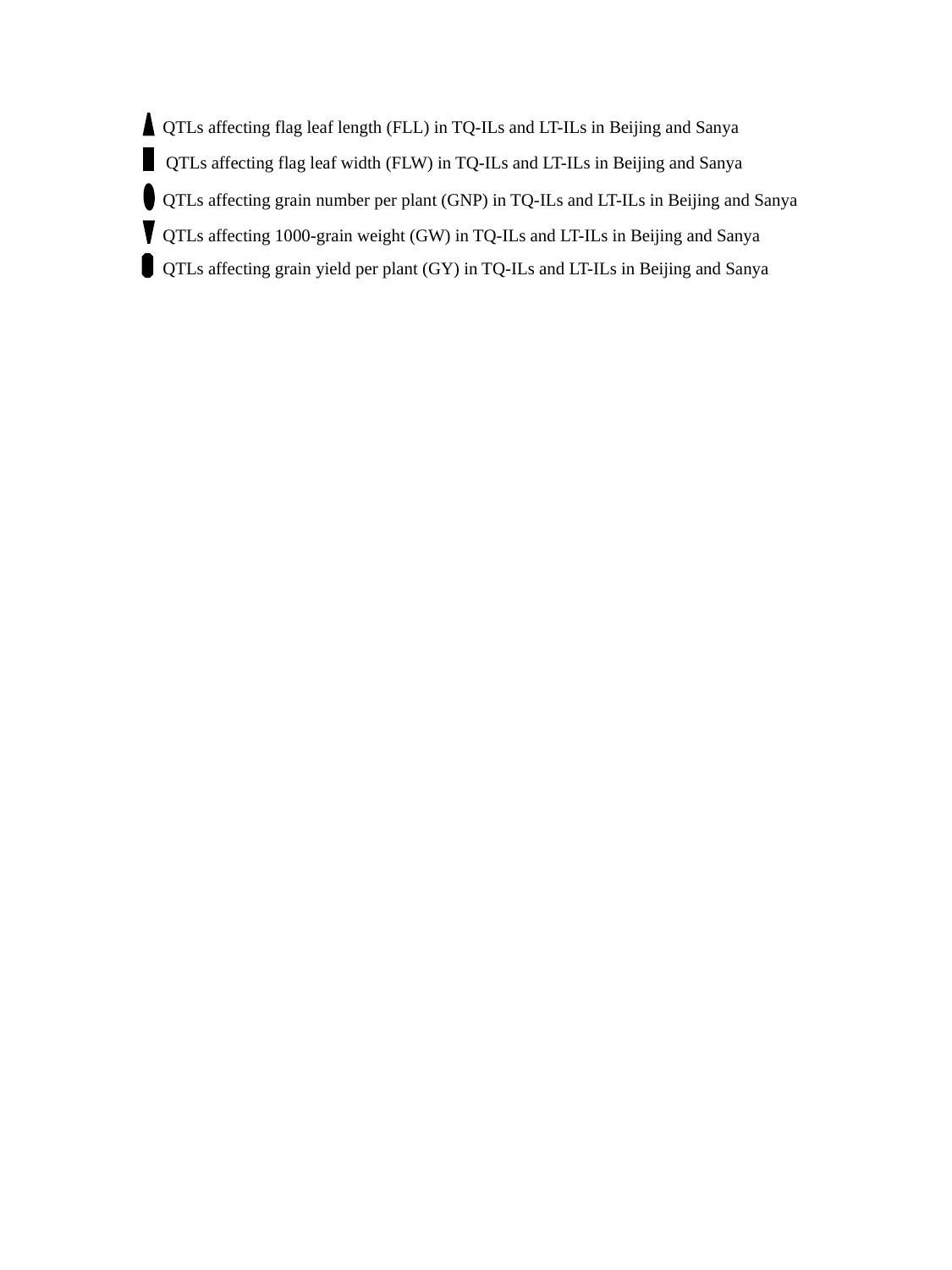

QTLs affecting flag leaf length (FLL) in TQ-ILs and LT-ILs in Beijing and Sanya
QTLs affecting flag leaf width (FLW) in TQ-ILs and LT-ILs in Beijing and Sanya
QTLs affecting grain number per plant (GNP) in TQ-ILs and LT-ILs in Beijing and Sanya
QTLs affecting 1000-grain weight (GW) in TQ-ILs and LT-ILs in Beijing and Sanya
QTLs affecting grain yield per plant (GY) in TQ-ILs and LT-ILs in Beijing and Sanya
