# Supplementary material for: SS1 (NAL1)- and SS2-Mediated Genetic Networks Underlying Source-Sink and Yield Traits in Rice (Oryza sativa L.)
Source: PLoS One. 2015 Jul 10;10(7):e0132060. doi: 10.1371/journal.pone.0132060 (PMC4498882; doi:10.1371/journal.pone.0132060)
Supplement: S2 Fig — (PPTX) [file pone.0132060.s002.pptx]

## Slide 1
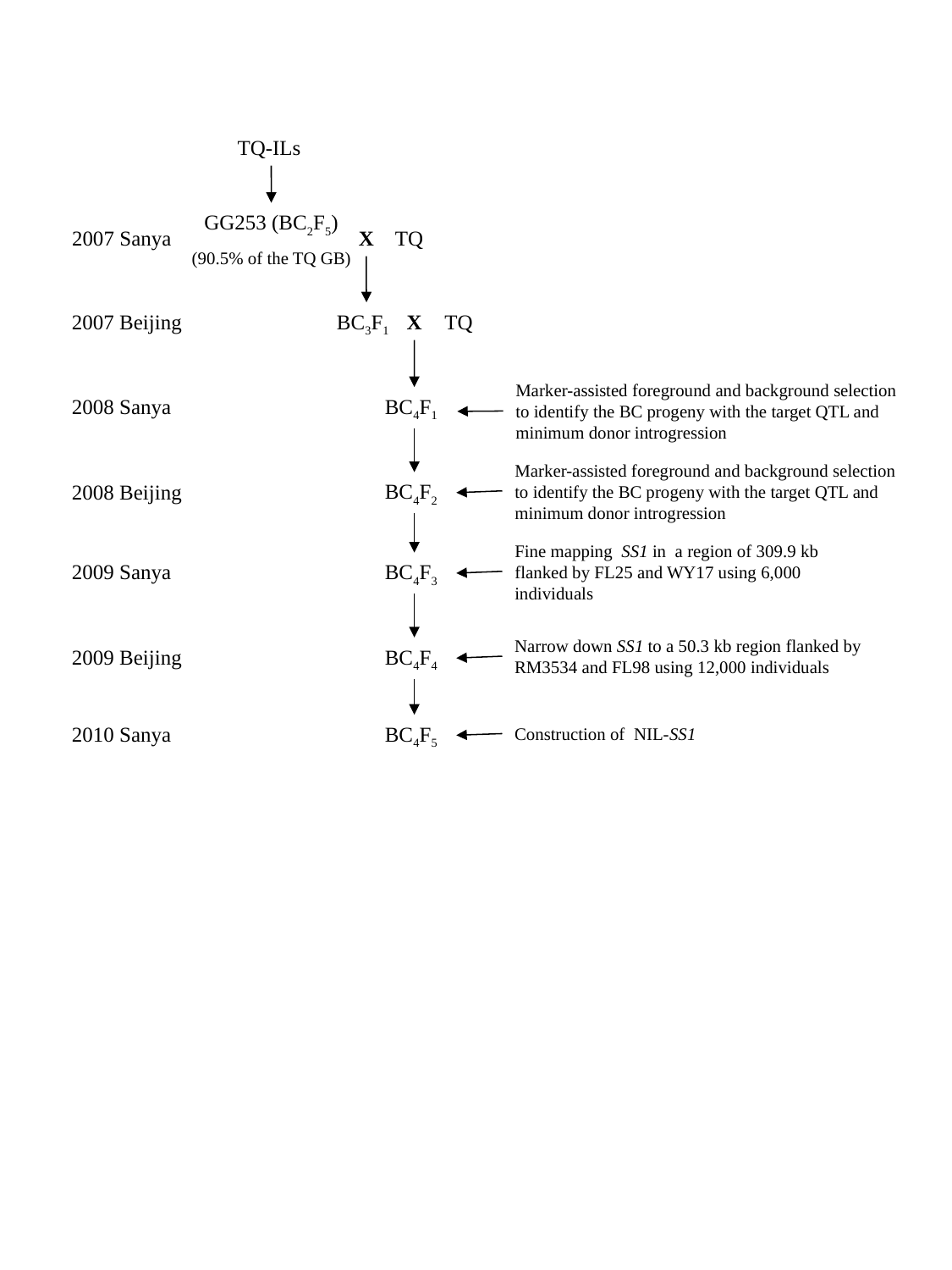

TQ-ILs
GG253 (BC2F5)
(90.5% of the TQ GB)
2007 Sanya
X
TQ
2007 Beijing
BC3F1
X
TQ
Marker-assisted foreground and background selection to identify the BC progeny with the target QTL and minimum donor introgression
2008 Sanya
BC4F1
Marker-assisted foreground and background selection to identify the BC progeny with the target QTL and minimum donor introgression
2008 Beijing
BC4F2
Fine mapping SS1 in a region of 309.9 kb
flanked by FL25 and WY17 using 6,000 individuals
2009 Sanya
BC4F3
Narrow down SS1 to a 50.3 kb region flanked by RM3534 and FL98 using 12,000 individuals
2009 Beijing
BC4F4
2010 Sanya
BC4F5
Construction of NIL-SS1
