# Supplementary material for: SS1 (NAL1)- and SS2-Mediated Genetic Networks Underlying Source-Sink and Yield Traits in Rice (Oryza sativa L.)
Source: PLoS One. 2015 Jul 10;10(7):e0132060. doi: 10.1371/journal.pone.0132060 (PMC4498882; doi:10.1371/journal.pone.0132060)
Supplement: S3 Fig — The gray regions indicate the coding region. The red bars indicate the substitutions and deletions between LT, NIL (NIL-SS1) and TQ. Asterisks indicate complete homology; semicolons indicate substitution of DNA sequences; and spaces indicate a complete lack of homology. Integers on the right indicate the cumulative number of nucleotide. (DOCX) [file pone.0132060.s003.docx]

LOC_Os04g52440-LT AAACGGATTCGAACTCAGGCCGGTAGGCACGCTGCTCCTGACAGCTCCCTGCTAGCTGAT

LOC_Os04g52440-NIL AAACGGATTCGAACTCAGGCCGGTAGGCACGCTGCTCCTGACAGCTCCCTGCTAGCTGAT

LOC_Os04g52440-TQ AAACAGATTCGAACTCAGGCCGGTAGGCACGCTGCTCCTGGCAGCTCCCTGCTAGCTGAT

****:***********************************:*******************

LOC_Os04g52440-LT CTACGTGCTGTTCTCTACACGTGGAAAATGAAGAAGTAGAAGTTAATATCAGGTAGTATA

LOC_Os04g52440-NIL CTACGTGCTGTTCTCTACACGTGGAAAATGAAGAAGTAGAAGTTAATATCAGGTAGTATA

LOC_Os04g52440-TQ CTACGTGCTGTTCTCTACACGTGGAAAATGAAGAAGTAGAAGTTGATATCAGGTAGTATA

********************************************:***************

LOC_Os04g52440-LT GAAACATACCTTAAGGAAATACTAGGTCTCTAAATCGTTGCCTGCTATAATAATGTACCA

LOC_Os04g52440-NIL GAAACATACCTTAAGGAAATACTAGGTCTCTAAATCGTTGCCTGCTATAATAATGTACCA

LOC_Os04g52440-TQ GAAACATACCTTAAGGAAATACTAGGTCTCTAAATCATTGCCTGCTAGAATAATGTACCA

************************************:**********:************

LOC_Os04g52440-LT GAGGTTACAAAAGAATAATTTATAAATTAGAAAGTTGGAAAAATGGTCTTAGAAAGATTG

LOC_Os04g52440-NIL GAGGTTACAAAAGAATAATTTATAAATTAGAAAGTTGGAAAAATGGTCTTAGAAAGATTG

LOC_Os04g52440-TQ GAGGTTACAAAAGAATAATTTATAAATTAGAAAGTTGGAAAAATGGTCTTAGAAAGACTG

*********************************************************:**

LOC_Os04g52440-LT AGAAGAAGTATTTATAAATAAATTTTTAAAAATATATCATCTAGTAGTTTAGAAAGCATA

LOC_Os04g52440-NIL AGAAGAAGTATTTATAAATAAATTTTTAAAAATATATCATCTAGTAGTTTAGAAAGCATA

LOC_Os04g52440-TQ AGAAGAAGTATTTATAAATGAATTTTTAAAAATATATCATTTAGTAGTTTAGAAAGCATA

*******************:********************:*******************

LOC_Os04g52440-LT TGGACGATACAATCCATAGTTCATAAGCCCGAAAATAACGGGCACATTAGCACATAACGC

LOC_Os04g52440-NIL TGGACGATACAATCCATAGTTCATAAGCCCGAAAATAACGGGCACATTAGCACATAACGC

LOC_Os04g52440-TQ TGGACGATACAATCCATAGTTCATAAGCCCGAAAATAACGGGCACATTAGCACATAACAC

**********************************************************:*

LOC_Os04g52440-LT TGTAGATGTAGCATCGCATAATTAATTAATTATTAGCTTAAAAAATAAAAAATAATATGA

LOC_Os04g52440-NIL TGTAGATGTAGCATCGCATAATTAATTAATTATTAGCTTAAAAAATAAAAAATAATATGA

LOC_Os04g52440-TQ TGTAGATGTAGCATCGCATAATTAATTAATTATTAGCT-AAAAAATAAAAAATAATATGA

************************************** *********************

LOC_Os04g52440-LT TTTTTTCAAGCAAATTTTTTATAGAACTTTTTTTTAAAAAAAATCATACTCCAAAAACAT

LOC_Os04g52440-NIL TTTTTTCAAGCAAATTTTTTATAGAACTTTTTTTTAAAAAAAATCATACTCCAAAAACAT

LOC_Os04g52440-TQ TTTTTTCAAGCAAATTTTTTATAGAACTTTTTTTTAAAAAAAATCATACTCCAAAAACAT

************************************************************

LOC_Os04g52440-LT GCACGCGGAAAATGAAGTAGAAGTTAGAGAACGAGTAGTATAGAACAGAGCAGGTCTCTA

LOC_Os04g52440-NIL GCACGCGGAAAATGAAGTAGAAGTTAGAGAACGAGTAGTATAGAACAGAGCAGGTCTCTA

LOC_Os04g52440-TQ GCACGCGGAAAATGAAGTAGAAGTTAGAGAACGAGTAGTATAGAACAGAGCAGGTCTCTA

************************************************************

LOC_Os04g52440-LT AATCGTTGCCTGCCGGAATAATCTGCCAGAGGTTACAAAAGAATAATCTATAAATTAGAA

LOC_Os04g52440-NIL AATCGTTGCCTGCCGGAATAATCTGCCAGAGGTTACAAAAGAATAATCTATAAATTAGAA

LOC_Os04g52440-TQ AATCGTTGCCTGCCGGAATAATCTGCCAGAGGTTACAAAAGAATAATCTATAAATTAGAA

************************************************************

LOC_Os04g52440-LT AGTTGGAAAAATTATCTTAGAAAGATTGAGAAGAAGTATTTATAAAGGTTTTTTTTTTCT

LOC_Os04g52440-NIL AGTTGGAAAAATTATCTTAGAAAGATTGAGAAGAAGTATTTATAAAGGTTTTTTTTTTCT

LOC_Os04g52440-TQ AGTTGGAAAAATTATCTTAGAAAGATTGAGAAGAAGTATTTATAAAG-TTTTTTTTTTCT

*********************************************** ************

LOC_Os04g52440-LT AAATATATCGTTTAATAGTTTAGAAAGACGGTACAATCCACAGTTCATAAGCCCGAAAAT

LOC_Os04g52440-NIL AAATATATCGTTTAATAGTTTAGAAAGACGGTACAATCCACAGTTCATAAGCCCGAAAAT

LOC_Os04g52440-TQ AAATATATCGTTTAGTAGTCTAGAAAGACGGTACAATCCACAGTTCATAAGCCCGAAAAT

**************:****:****************************************

LOC_Os04g52440-LT AACCGACACCTAACGCTGTAGCAGTAGCATCGCATCTGCTCAGCTCTAGAGTGGAGGATT

LOC_Os04g52440-NIL AACCGACACCTAACGCTGTAGCAGTAGCATCGCATCTGCTCAGCTCTAGAGTGGAGGATT

LOC_Os04g52440-TQ AACCGACACCTAACGCTGTAGCAGTAGCATCGCATCTGCTCAGCTCTAGAGTGGAGGATT

************************************************************

LOC_Os04g52440-LT TTCAGGAAGTTAAACTAATCTCTGTATAAATATACAAAAATTATCCGAGTTTACAAGAGG

LOC_Os04g52440-NIL TTCAGGAAGTTAAACTAATCTCTGTATAAATATACAAAAATTATCCGAGTTTACAAGAGG

LOC_Os04g52440-TQ TTCAGGAAGTTAAACTAATCTCTGGATAAATATACAAAAATTATCCGAGTTTACAAGAGG

************************:***********************************

LOC_Os04g52440-LT CCTTGAACTGCCACGGTTAGGACTTAGGACTTCTTCTGATGGGCCGTCTATGGCACGGCC

LOC_Os04g52440-NIL CCTTGAACTGCCACGGTTAGGACTTAGGACTTCTTCTGATGGGCCGTCTATGGCACGGCC

LOC_Os04g52440-TQ CCTTGAACTGCCACGGTTAGGACTTAGGACTTCTTCTGATGGGCCGTCTATGGCACGGCC

************************************************************

LOC_Os04g52440-LT CGTCTCGCAAAGCCATAATGGGCCCGGGAGCTTCACCAGCCCAATTTCCGGTGCCGCGAG

LOC_Os04g52440-NIL CGTCTCGCAAAGCCATAATGGGCCCGGGAGCTTCACCAGCCCAATTTCCGGTGCCGCGAG

LOC_Os04g52440-TQ CGTCTCGCAAAGCCATAATGGGCCCGGGAGCTTCACCAGCCCAATTTCCGGTGCCGCGAG

************************************************************

LOC_Os04g52440-LT CGCTCATGTGGGCCCCCTCCTTCAAGCTTCCAACTGCAACTTCCCCGGCGAGGCGGAGGC

LOC_Os04g52440-NIL CGCTCATGTGGGCCCCCTCCTTCAAGCTTCCAACTGCAACTTCCCCGGCGAGGCGGAGGC

LOC_Os04g52440-TQ CGCTCATGTGGGCCCCCTCCTTCAAGCTTCCAACCGCAACATCCCCGGCGAGGCGGAGGC

**********************************:*****:*******************

LOC_Os04g52440-LT GCCGCCGCCGC-----------------GGCGGCGGCGGCGGCGCAGCGTGAGACTAAGG

LOC_Os04g52440-NIL GCCGCCGCCGC-----------------GGCGGCGGCGGCGGCGCAGCGTGAGACTAAGG

LOC_Os04g52440-TQ GCCGCCGCCGCCGTGAAAATGTCACGGCGGCGGCGGCGGCGGCGCAGCGTGAGACTAAGG

*********** ********************************

LOC_Os04g52440-LT AAAGCCGATAGATCGGCGGAGTATCGTACGTGTCAGCGACGGCCTGCCTTCGAAGGCCGT

LOC_Os04g52440-NIL AAAGCCGATAGATCGGCGGAGTATCGTACGTGTCAGCGACGGCCTGCCTTCGAAGGCCGT

LOC_Os04g52440-TQ AAAGCCGATAGATCGGCGGAGTATCGTACGTGTCAGCGACGGCCTGCCTTCGAAGGCCGT

************************************************************

LOC_Os04g52440-LT GTGATCATGTTACTGAATGTCGATCGCCTAATCTTGTTTTCGACTGTTGATTAGCCAAGA

LOC_Os04g52440-NIL GTGATCATGTTACTGAATGTCGATCGCCTAATCTTGTTTTCGACTGTTGATTAGCCAAGA

LOC_Os04g52440-TQ GTGATCATGTTACTGAATGTCGATCGCCTAATCTTGTTTTCGACTGTTGATTAGCCAAGA

************************************************************

LOC_Os04g52440-LT GGGAGGGGATTATAGGCTTTCTTCTGTGGTAAGACGGGTCTCTGATTTTGTTCTTCCATC

LOC_Os04g52440-NIL GGGAGGGGATTATAGGCTTTCTTCTGTGGTAAGACGGGTCTCTGATTTTGTTCTTCCATC

LOC_Os04g52440-TQ GGGAGGGGATTATAGGCTTTCTTCTGTGGTAAGACGGGTCTCTGATTTTGTTCTTCCATC

************************************************************

LOC_Os04g52440-LT AGTTCCATGTCGGAAGAAGTTGCAATCATGATCTCTTTGGGAGTAGCATTCTTTGCATAG

LOC_Os04g52440-NIL AGTTCCATGTCGGAAGAAGTTGCAATCATGATCTCTTTGGGAGTAGCATTCTTTGCATAG

LOC_Os04g52440-TQ AGTTCCATGTCGGAAGAAGTTGCAATCATGATCTCTTTGGGAGTAGCATTCTTTGCATAG

************************************************************

LOC_Os04g52440-LT AAGCTTTTGTTTTGGTGTTCTTGTTCAGAATTTCACCTTCCACTGTCTGTCATATCACAG

LOC_Os04g52440-NIL AAGCTTTTGTTTTGGTGTTCTTGTTCAGAATTTCACCTTCCACTGTCTGTCATATCACAG

LOC_Os04g52440-TQ AAGCTTTTGTTTTGGTGTTCTTGTTCAGAATTTCACCTTCCACTGTCTGTCATATCACAG

************************************************************

LOC_Os04g52440-LT GTGTAAGCTTTTCATCACTGATTCATCATGAATTCCCCCTCATTTGAAAATCAACAGGCA

LOC_Os04g52440-NIL GTGTAAGCTTTTCATCACTGATTCATCATGAATTCCCCCTCATTTGAAAATCAACAGGCA

LOC_Os04g52440-TQ GTGTAAGCTTTTCATCACTGATTCATCATGAATTCCCCCTCATTTGAAAATCAACAGGCA

************************************************************

LOC_Os04g52440-LT ATGAATTTGATAAAGCATGCAGCTTTTGCTGCAAGTTTTCAAGGGGAAACGGATTGTACC 60

LOC_Os04g52440-NIL ATGAATTTGATAAAGCATGCAGCTTTTGCTGCAAGTTTTCAAGGGGAAACGGATTGTACC 60

LOC_Os04g52440-TQ ATGAATTTGATAAAGCATGCAGCTTTTGCTGCAAGTTTTCAAGGGGAAACGGATTGTACC 60

************************************************************

LOC_Os04g52440-LT TCACATGCATCAGCTAGGAAGTTCAGCACCTCAGGATCTTCCCCTCTGCTAGACTCAACC 120

LOC_Os04g52440-NIL TCACATGCATCAGCTAGGAAGTTCAGCACCTCAGGATCTTCCCCTCTGCTAGACTCAACC 120

LOC_Os04g52440-TQ TCACATGCATCAGCTAGGAAGTTCAGCACCTCAGGATCTTCCCCTCTGCTAGACTCAACC 120

************************************************************

LOC_Os04g52440-LT GAAGGAAATGGGTTTAAGGGACACTCCATGCTGGCACCATTCACTGCCGGATGGCACTCC 180

LOC_Os04g52440-NIL GAAGGAAATGGGTTTAAGGGACACTCCATGCTGGCACCATTCACTGCCGGATGGCACTCC 180

LOC_Os04g52440-TQ GAAGGAAATGGGTTTAAGGGACACTCCATGCTGGCACCATTCACTGCCGGATGGCACTCC 180

************************************************************

LOC_Os04g52440-LT ACAGACCTGGAGCCCTTGATTATTGAAAGATCAGAGGGTTCTTATGTCTATGATAGCAAG 240

LOC_Os04g52440-NIL ACAGACCTGGAGCCCTTGATTATTGAAAGATCAGAGGGTTCTTATGTCTATGATAGCAAG 240

LOC_Os04g52440-TQ ACAGACCTGGAGCCCTTGATTATTGAAAGATCAGAGGGTTCTTATGTCTATGATAGCAAG 240

************************************************************

LOC_Os04g52440-LT GGGAACAAGTACTTGGATACACTAGCAGGATTGTGGTGTACAGCCTTAGGTGGCAGTGAG 300

LOC_Os04g52440-NIL GGGAACAAGTACTTGGATACACTAGCAGGATTGTGGTGTACAGCCTTAGGTGGCAGTGAG 300

LOC_Os04g52440-TQ GGGAACAAGTACTTGGATACACTAGCAGGATTGTGGTGTACAGCCTTAGGTGGCAGTGAG 300

************************************************************

LOC_Os04g52440-LT CCTCGGTTAGTGAAAGCAGCAACCGACCAATTAAACAAGTTGCCGTTCTACCACTCCTTT 360

LOC_Os04g52440-NIL CCTCGGTTAGTGAAAGCAGCAACCGACCAATTAAACAAGTTGCCGTTCTACCACTCCTTT 360

LOC_Os04g52440-TQ CCTCGGTTAGTGAAAGCAGCAACCGACCAATTAAACAAGTTGCCGTTCTACCACTCCTTT 360

************************************************************

LOC_Os04g52440-LT TGGAACAGTACAGCCAAGCCACCATTGGATCTTGCTGAGGAACTTATCAGCATGTTCACT 420

LOC_Os04g52440-NIL TGGAACAGTACAGCCAAGCCACCATTGGATCTTGCTGAGGAACTTATCAGCATGTTCACT 420

LOC_Os04g52440-TQ TGGAACAGTACAGCCAAGCCACCATTGGATCTTGCTGAGGAACTTATCAGCATGTTCACT 420

************************************************************

LOC_Os04g52440-LT GCCAAGGAAATGGGAAAAGTGTTCTTTACAAACAGCGGTTCTGAAGCAAATGACTCTCAG 480

LOC_Os04g52440-NIL GCCAAGGAAATGGGAAAAGTGTTCTTTACAAACAGCGGTTCTGAAGCAAATGACTCTCAG 480

LOC_Os04g52440-TQ GCCAAGGAAATGGGAAAAGTGTTCTTTACAAACAGCGGTTCTGAAGCAAATGACTCTCAG 480

************************************************************

LOC_Os04g52440-LT GTCAAACTAGTATGGTACTATAACAATGCATTGGGGAGACCAAACAAGAAAAAGATCATT 540

LOC_Os04g52440-NIL GTCAAACTAGTATGGTACTATAACAATGCATTGGGGAGACCAAACAAGAAAAAGATCATT 540

LOC_Os04g52440-TQ GTCAAACTAGTATGGTACTATAACAATGCATTGGGGAGACCAAACAAGAAAAAGATCATT 540

************************************************************

LOC_Os04g52440-LT GCACAATCACAAGCATATCACGGATCAACATTAATATCAGCTAGTCTGTCTGGCCTCCCT 600

LOC_Os04g52440-NIL GCACAATCACAAGCATATCACGGATCAACATTAATATCAGCTAGTCTGTCTGGCCTCCCT 600

LOC_Os04g52440-TQ GCACAATCACAAGCATATCACGGATCAACATTAATATCAGCTAGTCTGTCTGGCCTCCCT 600

************************************************************

LOC_Os04g52440-LT GCGATGCATCTGAAGTTTGATCTACCAGCACCTTTTGTTCTGCACACAGACTGCCCTCAC 660

LOC_Os04g52440-NIL GCGATGCATCTGAAGTTTGATCTACCAGCACCTTTTGTTCTGCACACAGACTGCCCTCAC 660

LOC_Os04g52440-TQ GCGATGCATCTGAAGTTTGATCTACCAGCACCTTTTGTTCTGCACACAGACTGCCCTCAC 660

************************************************************

LOC_Os04g52440-LT TACTGGCGCTTCGGTCTTCCTGGTGAGGCAGAAGAAGAATTTGCAACCAGACTTGCCGAT 720

LOC_Os04g52440-NIL TACTGGCGCTTCGGTCTTCCTGGTGAGGCAGAAGAAGAATTTGCAACCAGACTTGCCGAT 720

LOC_Os04g52440-TQ TACTGGCGCTTCGGTCTTCCTGGTGAGGCAGAAGAAGAATTTGCAACCAGACTTGCCGAT 720

************************************************************

LOC_Os04g52440-LT AATTTAGAGAATCTTATCCTCAAAGAAGGGCCAGAAACAGTTGCTGCTTTCATTGCTGAA 780

LOC_Os04g52440-NIL AATTTAGAGAATCTTATCCTCAAAGAAGGGCCAGAAACAGTTGCTGCTTTCATTGCTGAA 780

LOC_Os04g52440-TQ AATTTAGAGAATCTTATCCTCAAAGAAGGGCCAGAAACAGTTGCTGCTTTCATTGCTGAA 780

************************************************************

LOC_Os04g52440-LT CCTGTAATTGGTGCTGGAGGTGTCATCCCTCCTCCAAAGACATATTTTGAGAAGATTCAA 840

LOC_Os04g52440-NIL CCTGTAATTGGTGCTGGAGGTGTCATCCCTCCTCCAAAGACATATTTTGAGAAGATTCAA 840

LOC_Os04g52440-TQ CCTGTAATTGGTGCTGGAGGTGTCATCCCTCCTCCAAAGACATATTTTGAGAAGATTCAA 840

************************************************************

LOC_Os04g52440-LT GCAGTACTTCAGAAGTACGATGTCCTTTTCATAGCAGATGAGGTAATTACTGGATTTGGA 900

LOC_Os04g52440-NIL GCAGTACTTCAGAAGTACGATGTCCTTTTCATAGCAGATGAGGTAATTACTGGATTTGGA 900

LOC_Os04g52440-TQ GCAGTACTTCAGAAGTACGATGTCCTTTTCATAGCAGATGAGGTAATTACTGGATTTGGA 900

************************************************************

LOC_Os04g52440-LT CGGCTGGGAACCATGTTTGGATCTGATCTGTACAACATCAAGCCAGATCTCGTCTCCTTA 960

LOC_Os04g52440-NIL CGGCTGGGAACCATGTTTGGATCTGATCTGTACAACATCAAGCCAGATCTCGTCTCCTTA 960

LOC_Os04g52440-TQ CGGCTGGGAACCATGTTTGGATCTGATCTGTACAACATCAAGCCAGATCTCGTCTCCTTA 960

************************************************************

LOC_Os04g52440-LT GCAAAGGCACTCTCATCTGCGTATGTCCCAATTGGAGCGACTCTTGTCAGCCCAGAAATA 1020

LOC_Os04g52440-NIL GCAAAGGCACTCTCATCTGCGTATGTCCCAATTGGAGCGACTCTTGTCAGCCCAGAAATA 1020

LOC_Os04g52440-TQ GCAAAGGCACTCTCATCTGCGTATGTCCCAATTGGAGCGACTCTTGTCAGCCCAGAAATA 1020

************************************************************

LOC_Os04g52440-LT TCCGATGTAGTTCATTCTCAGAGCAATAAGATTGGCTTTTTTGCTCATGGCTTTACATAC 1080

LOC_Os04g52440-NIL TCCGATGTAGTTCATTCTCAGAGCAATAAGATTGGCTTTTTTGCTCATGGCTTTACATAC 1080

LOC_Os04g52440-TQ TCCGATGTAGTTCATTCTCAGAGCAATAAGATTGGCTTTTTTGCTCATGGCTTTACATAC 1080

************************************************************

LOC_Os04g52440-LT TCTGGCCATCCAGTTTCTTGTGCTGTCGCCCTAGAAGCTCTGAAAATTTATCGGGAAAGG 1140

LOC_Os04g52440-NIL TCTGGCCATCCAGTTTCTTGTGCTGTCGCCCTAGAAGCTCTGAAAATTTATCGGGAAAGG 1140

LOC_Os04g52440-TQ TCTGGCCATCCAGTTTCTTGTGCTGTCGCCCTAGAAGCTCTGAAAATTTATCGGGAAAGG 1140

************************************************************

LOC_Os04g52440-LT AACATCCCTGCTCATGTCAAGCAAATTTCTCCAAGGTTCCAGGAGGGAATCAAGGCCTTC 1200

LOC_Os04g52440-NIL AACATCCCTGCTCATGTCAAGCAAATTTCTCCAAGGTTCCAGGAGGGAATCAAGGCCTTC 1200

LOC_Os04g52440-TQ AACATCCCTGCTCATGTCAAGCAAATTTCTCCAAGGTTCCAGGAGGGAATCAAGGCCTTC 1200

************************************************************

LOC_Os04g52440-LT GCGGGAAGTTCAATTATAGGAGAGACACGTGGTGTAGGTTTGCTGCTCGCGACTGAATTT 1260

LOC_Os04g52440-NIL GCGGGAAGTTCAATTATAGGAGAGACACGTGGTGTAGGTTTGCTGCTCGCGACTGAATTT 1260

LOC_Os04g52440-TQ GCGGGAAGTTCAATTATAGGAGAGACACGTGGTGTAGGTTTGCTGCTCGCGACTGAATTT 1260

************************************************************

LOC_Os04g52440-LT GCTAATAACAAATCACCAAATGATCCATTTCCTGTTGAGTGGGGAGTTGCGCAAATCTTT 1320

LOC_Os04g52440-NIL GCTAATAACAAATCACCAAATGATCCATTTCCTGTTGAGTGGGGAGTTGCGCAAATCTTT 1320

LOC_Os04g52440-TQ GCTAATAACAAATCACCAAATGATCCATTTCCTGTTGAGTGGGGAGTTGCGCAAATCTTT 1320

************************************************************

LOC_Os04g52440-LT GGAGCAGAGTGTAAGAAGCGTGGTATGCTAGTTAAGGTTGTTGGAGATGAGATCGCCATG 1380

LOC_Os04g52440-NIL GGAGCAGAGTGTAAGAAGCGTGGTATGCTAGTTAAGGTTGTTGGAGATGAGATCGCCATG 1380

LOC_Os04g52440-TQ GGAGCAGAGTGTAAGAAGCGTGGTATGCTAGTTAAGGTTGTTGGAGATGAGATCGCCATG 1380

************************************************************

LOC_Os04g52440-LT TCGCCACCACTAATAATGAGCCAAAGAGAAGTTGATGGACTGGTGAGCATATACGGTGAA 1440

LOC_Os04g52440-NIL TCGCCACCACTAATAATGAGCCAAAGAGAAGTTGATGGACTGGTGAGCATATACGGTGAA 1440

LOC_Os04g52440-TQ TCGCCACCACTAATAATGAGCCAAAGAGAAGTTGATGGACTGGTGAGCATATACGGTGAA 1440

************************************************************

LOC_Os04g52440-LT GCTTTGAAGGCCACCGAGGAAAGAGTGGCAGAGCTGAGGTCCAAGAAAAAATAG 1494

LOC_Os04g52440-NIL GCTTTGAAGGCCACCGAGGAAAGAGTGGCAGAGCTGAGGTCCAAGAAAAAATAG 1494

LOC_Os04g52440-TQ GCTTTGAAGGCCACCGAGGAAAGAGTGGCAGAGCTGAGGTCCAAGAAAAAATAG 1494

******************************************************
