# Supplementary material for: SS1 (NAL1)- and SS2-Mediated Genetic Networks Underlying Source-Sink and Yield Traits in Rice (Oryza sativa L.)
Source: PLoS One. 2015 Jul 10;10(7):e0132060. doi: 10.1371/journal.pone.0132060 (PMC4498882; doi:10.1371/journal.pone.0132060)
Supplement: S4 Fig — The gray regions indicate the coding region. The red bars indicate the substitutions and deletions between LT, NIL (NIL-SS1) and TQ. Asterisks indicate complete homology; semicolons indicate substitution of DNA sequences; and spaces indicate complete lack of homology. Integers on the right indicate the cumulative number of nucleotides in the coding region. (DOCX) [file pone.0132060.s004.docx]

LOC_Os04g52450-LT ACGAGACCGACCGAGTGACAGGTGAGCAGTGCAAACGGCAAAGCGTGTATCAGCGCACAT

LOC_Os04g52450-NIL ACGAGACCGACCGAGTGACAGGTGAGCAGTGCAAACGGCAAAGCGTGTATCAGCGCACAT

LOC_Os04g52450-TQ ACGAGACCGACCGAGTGACAGGTGAGCAGTGCAAACGGCAAAGCGTGTATCAGCGCACAT

************************************************************

LOC_Os04g52450-LT GATGATGCGAAACGCTATCTGCTTGGCATTCATCTGCAACTTGGCAACTTGATTTGCGGG

LOC_Os04g52450-NIL GATGATGCGAAACGCTATCTGCTTGGCATTCATCTGCAACTTGGCAACTTGATTTGCGGG

LOC_Os04g52450-TQ GATGATGCGAAACGCTATCTGCTTGGCATTCATCTGCAACTTGGCAACTTGATTTGCGGG

************************************************************

LOC_Os04g52450-LT TGCGATGTACTCTCTCCATTAAAAAAAAACACACAAACACTAAGTTTCCGTATTTAACGT

LOC_Os04g52450-NIL TGCGATGTACTCTCTCCATTAAAAAAAAACACACAAACACTAAGTTTCCGTATTTAACGT

LOC_Os04g52450-TQ TGCGATGTACTCTCTCCATGAAAAAAAA---CACAAACACTAAGTTTCCGTATTCAACGT

******************* ******** *********************** *****

LOC_Os04g52450-LT TTGACTGTGTATTTTATATAAATTTTTTTTATAATTAGTATTTTTTTTGTTGTTACATGA

LOC_Os04g52450-NIL TTGACTGTGTATTTTATATAAATTTTTTTTATAATTAGTATTTTTTTTGTTGTTACATGA

LOC_Os04g52450-TQ TTGACTGTGCATCTTATATAAATTTTTTTTATAATTAGTATTTTCATTGTTGTTAGATGA

*********:**:*******************************::*********:****

LOC_Os04g52450-LT TAAAACATGATTAATACTTTATACATGATTTGTCTTTTTATTTTTTTATAATTTTTTTAA

LOC_Os04g52450-NIL TAAAACATGATTAATACTTTATACATGATTTGTCTTTTTATTTTTTTATAATTTTTTTAA

LOC_Os04g52450-TQ TAAAACATGATTAATACTTTATGCGTGATTTGTCTTTTTATTTTTTTATAATTTTTT-AA

**********************:*:******************************** **

LOC_Os04g52450-LT ATAAGACAAACAGTTAAACGTTGGA-ACGGAAACTCAAGGTTTATCTTTTTTTTTTGGAA

LOC_Os04g52450-NIL ATAAGACAAACAGTTAAACGTTGGA-ACGGAAACTCAAGGTTTATCTTTTTTTTTTGGAA

LOC_Os04g52450-TQ ATAAGACAGACGGTCAAACGTTGGACACGAAAACTCAAGGTTTA--TTTTTTTTTAAGTA

********:**:**:********** ***:************** *********::*:*

LOC_Os04g52450-LT CGGAGGGAGTATTATTTGGTCACGAGTGAGCATGATTTGATTCGCACAAGCACACCTGAA

LOC_Os04g52450-NIL CGGAGGGAGTATTATTTGGTCACGAGTGAGCATGATTTGATTCGCACAAGCACACCTGAA

LOC_Os04g52450-TQ CGGAGGGAGTATTATTTGGTCACGAGTGAGCATGATTTGATTCGCACAAGCACACCTGAA

************************************************************

LOC_Os04g52450-LT GTACTGTACTCCATTTTGCAAAATTTCTTCAAAATCCGTGTGGGACATTAGGCTTCCAAA

LOC_Os04g52450-NIL GTACTGTACTCCATTTTGCAAAATTTCTTCAAAATCCGTGTGGGACATTAGGCTTCCAAA

LOC_Os04g52450-TQ GTACTGTACTTCATTTTGCAAAATTTCTTCAAAATCCGTGTGGGACATTAGGCTCCCAAA

**********:*******************************************:*****

LOC_Os04g52450-LT AAAAAAGGCACATGAACTTTTGTTTGAATCAAACAACTGCCAGGGTGTACTCAACTACAC

LOC_Os04g52450-NIL AAAAAAGGCACATGAACTTTTGTTTGAATCAAACAACTGCCAGGGTGTACTCAACTACAC

LOC_Os04g52450-TQ AAAAAAGGCACATGAATTTTTGTTTGAATCAAACAACTGCCAGGGTGTACTCAACTACAC

****************:*******************************************

LOC_Os04g52450-LT ATGGCTTCAATTCTGTAGACGCCATCTCTGCAAGACAA—TCTAGAGAGGGAAGGAGATA

LOC_Os04g52450-NIL ATGGCTTCAATTCTGTAGACGCCATCTCTGCAAGACAA--TCTAGAGAGGGAAGGAGATA

LOC_Os04g52450-TQ ATGGCTTCAATTCTGTAGACGCCACCTCTGCAAGACAAAATCTAGAGAGGGAAGGAGATA

************************ ************* ********************

LOC_Os04g52450-LT AACTCGTGACAAAGCAGACACGTGCTCAATGCGTAACAAG-ACACAAGCGTTTGAACTTG

LOC_Os04g52450-NIL AACTCGTGACAAAGCAGACACGTGCTCAATGCGTAACAAG-ACACAAGCGTTTGAACTTG

LOC_Os04g52450-TQ AACTCGTGACAAAGCAGACACGTGCTCAATGCGTAACAAGGACACAAGCGTTTGAACTTG

**************************************** *******************

LOC_Os04g52450-LT GATTCCCTGGCTACAGTCATTAAAATTAAATTTTTCCGGAAGCAAACAGTGCATTTAAAA

LOC_Os04g52450-NIL GATTCCCTGGCTACAGTCATTAAAATTAAATTTTTCCGGAAGCAAACAGTGCATTTAAAA

LOC_Os04g52450-TQ GATTCCCTGGCTACAGTCAATAAAATTAAATTTTTCCGGAAGCAAACAGTGCATTTAAAA

*******************:****************************************

LOC_Os04g52450-LT ACCAAAAGATCTTGCTCAACTTGATTCGTTTCAAAAACTGAAATCTATTACCTCCGTCCA

LOC_Os04g52450-NIL ACCAAAAGATCTTGCTCAACTTGATTCGTTTCAAAAACTGAAATCTATTACCTCCGTCCA

LOC_Os04g52450-TQ ACCAAAAGATCTTGCTCAACTTGATTCGTTTCAAAAACTGAAATCTATTACCTCCGTCCA

************************************************************

LOC_Os04g52450-LT CAAAGAGTGCAGTTTTGTACTTTTCATATTCAATGTTTGATCGTCCGTC----TTATTTG

LOC_Os04g52450-NIL CAAAGAGTGCAGTTTTGTACTTTTCATATTCAATGTTTGATCGTCCGTC----TTATTTG

LOC_Os04g52450-TQ CAAAGAGTGCAGTTTTGTACTTTTCATATTCAATGTTTGATCGTCCGTCCGTCTTATTTG

************************************************* *******

LOC_Os04g52450-LT AAGATTTTTTATGATTAGTATTTT----------------------------------TT

LOC_Os04g52450-NIL AAGATTTTTTATGATTAGTATTTT----------------------------------TT

LOC_Os04g52450-TQ AAGATTTTTTATGATTAGTATTTTCGTTTTATTTGAAGATTTTTTATGATTAGTATTTTT

************************ **

LOC_Os04g52450-LT ATAATTAGATGATAAAACATAAATAATATTTAATGTTTAACTATTTTTTAAAAATAATAT

LOC_Os04g52450-NIL ATAATTAGATGATAAAACATAAATAATATTTAATGTTTAACTATTTTTTAAAAATAATAT

LOC_Os04g52450-TQ TTAATTAGATGATAAAACATAAATAATATTTAATGTTTAACTATTTTTTAAAAATAATAT

************************************************************

LOC_Os04g52450-LT AA-TTTTTTAAATTGACAGACGGTTAAATGTTGATATAGAAGCAACGACTTCACTTGAAT

LOC_Os04g52450-NIL AA-TTTTTTAAATTGACAGACGGTTAAATGTTGATATAGAAGCAACGACTTCACTTGAAT

LOC_Os04g52450-TQ AAATTTTTTAAATTGACGGACGGTTAAATGTCGATATAGAAGGAACGACTTCACTTGAAT

** **************:*************:**********:*****************

LOC_Os04g52450-LT TAGAATGGAGGTAGTATTTTATAAGAGCAATTCTACGGTTCTTGAGGAGGTACCATGAGA

LOC_Os04g52450-NIL TAGAATGGAGGTAGTATTTTATAAGAGCAATTCTACGGTTCTTGAGGAGGTACCATGAGA

LOC_Os04g52450-TQ TGGGATGGAGGTAGTATTTTATAAGAGCAATTCTATAGTCCTTGAGGAGGTACCATGAGG

*:*:*******************************::**:*******************:

LOC_Os04g52450-LT TACTAAAAATTTAGTATAAAATTTGATACCTCATGGTATCTCATAGTACCTAGTACTAAT

LOC_Os04g52450-NIL TACTAAAAATTTAGTATAAAATTTGATACCTCATGGTATCTCATAGTACCTAGTACTAAT

LOC_Os04g52450-TQ TAACAAAAATTTAGTATAAAATTTAATACCTCATGGTACCTCATAGTACCTAGTACTAAT

**::********************:*************:*********************

LOC_Os04g52450-LT GAGGTATCACTTTTTATCTCCTCAAGAACCATAAAATTGCTCATTTTATAATTATCCTTT

LOC_Os04g52450-NIL GAGGTATCACTTTTTATCTCCTCAAGAACCATAAAATTGCTCATTTTATAATTATCCTTT

LOC_Os04g52450-TQ AAGGTACCACTTTTTATCTCTTCAGGAACCATAAAATTGCTCATTTTATAATTATCTTTT

******:************:****:*******************************:***

LOC_Os04g52450-LT TTTTTCTTTTTTTGGTTTTGGGACACTTCAGCTGGAGAAGGAGAAGAACAGTCTGCACTC

LOC_Os04g52450-NIL TTTTTCTTTTTTTGGTTTTGGGACACTTCAGCTGGAGAAGGAGAAGAACAGTCTGCACTC

LOC_Os04g52450-TQ TTTTTCTTTTTTTGGTTTTGGGACACTTCAGCTGGAGAAGGAGAAGAACAGTCTGCACTC

************************************************************

LOC_Os04g52450-LT TGCACACGTCCCCATCTCCCCCGTCTCTCCTGCGTTCTCTTCGCCTACCTTACGATAAAC

LOC_Os04g52450-NIL TGCACACGTCCCCATCTCCCCCGTCTCTCCTGCGTTCTCTTCGCCTACCTTACGATAAAC

LOC_Os04g52450-TQ TGCACACGTCCCCATCTCCCCCGTCTCTCCTGCGTTCTCTTCGCCTACCTTACGATAAAC

************************************************************

LOC_Os04g52450-LT TTGGCCACGTCTCAATCTCTCTCTCTC—GCGCTCTCCTCTCCTTCTATAATACTCGGCC

LOC_Os04g52450-NIL TTGGCCACGTCTCAATCTCTCTCTCTC--GCGCTCTCCTCTCCTTCTATAATACTCGGCC

LOC_Os04g52450-TQ TTGGCCACGTCTCAATCTCTCTCTCTCTCGCGCTCTCCTCTCCTTCTATAATACTCGGCC

*************************** *******************************

LOC_Os04g52450-LT TCCCCCTCGCTTCTCGCTCTGTTTCTCCGAGAATTAGGGGGGAGGTTGGAGCTGTGCAAG

LOC_Os04g52450-NIL TCCCCCTCGCTTCTCGCTCTGTTTCTCCGAGAATTAGGGGGGAGGTTGGAGCTGTGCAAG

LOC_Os04g52450-TQ TCCCCCTCGCTTCTCGCTCTGTTTCTCCGAGAATTAGGGGGGAGGTTGGAGCTGTGCAAG

************************************************************

LOC_Os04g52450-LT ATGGTGATTTCGCGCGGCCTTCTCCGATCGAATGCCTCTTCTTCCTCCTCTCAGGCAATC 60

LOC_Os04g52450-NIL ATGGTGATTTCGCGCGGCCTTCTCCGATCGAATGCCTCTTCTTCCTCCTCTCAGGCAATC 60

LOC_Os04g52450-TQ ATGGTGATTTCGCGCGGCCTTCTCCGATCGAATGCCTCTTCTTCCTCCTCTCAGGCAATC 60

************************************************************

LOC_Os04g52450-LT AACTTATTGAAGTATGTAACTAGCACTGGAAGTCTGCAAGGGCACTCGCAAAATTTGTGT 120

LOC_Os04g52450-NIL AACTTATTGAAGTATGTAACTAGCACTGGAAGTCTGCAAGGGCACTCGCAAAATTTGTGT 120

LOC_Os04g52450-TQ AACTTATTGAAGTATGTAACTAGCACTGGAAGTCTGCAAGGGCACACGCAAAATTTGTGT 120

*********************************************:**************

LOC_Os04g52450-LT GATGCATCAACCAGACATTTCAGTTCAGTACCATCGCCCCAGTACAACTCAACTGAAGAG 180

LOC_Os04g52450-NIL GATGCATCAACCAGACATTTCAGTTCAGTACCATCGCCCCAGTACAACTCAACTGAAGAG 180

LOC_Os04g52450-TQ GATGCATCAACCAGACATTTCAGTTCAGTACCATCGCCCCAGTCCAACTCAACTGAAGAG 180

*******************************************:****************

LOC_Os04g52450-LT AATGGGTTCAAGGGGCATGGCATGTTGGCACCATTCACAGCTGGTTGGCAGAGCACCGAT 240

LOC_Os04g52450-NIL AATGGGTTCAAGGGGCATGGCATGTTGGCACCATTCACAGCTGGTTGGCAGAGCACCGAT 240

LOC_Os04g52450-TQ AATGGGTTCAAGGGGCATGGCATGTTGGCACCATTCACAGCTGGTTGGCAGAGCACCGAT 240

************************************************************

LOC_Os04g52450-LT GTTCATCCGTTAGTCATTGAAAGATCTGAGGGTTCGTATGTATATGATATCGACGGAAAG 300

LOC_Os04g52450-NIL GTTCATCCGTTAGTCATTGAAAGATCTGAGGGTTCGTATGTATATGATATCGACGGAAAG 300

LOC_Os04g52450-TQ GTTCATCCGTTAGTCATTGAAAGATCTGAGGGTTCGTATGTATATGATATCGACGGAAAG 300

************************************************************

LOC_Os04g52450-LT AAGTATCTAGATTCTCTTGCGGGACTATGGTGTACAGCTTTAGGTGGTAGTGAACCTCGA 360

LOC_Os04g52450-NIL AAGTATCTAGATTCTCTTGCGGGACTATGGTGTACAGCTTTAGGTGGTAGTGAACCTCGA 360

LOC_Os04g52450-TQ AAGTATCTAGATTCTCTTGCGGGACTATGGTGTACAGCTTTAGGTGGTAGTGAACCTCGA 360

************************************************************

LOC_Os04g52450-LT TTAGTCAAAGCTGCAACTGAGCAATTACACAAGTTGCCCTTCTATCACTCCTTTTGGAAC 420

LOC_Os04g52450-NIL TTAGTCAAAGCTGCAACTGAGCAATTACACAAGTTGCCCTTCTATCACTCCTTTTGGAAC 420

LOC_Os04g52450-TQ TTAGTCAAAGCTGCAACTGAGCAATTACACAAGTTGCCCTTCTATCACTCCTTTTGGAAC 420

************************************************************

LOC_Os04g52450-LT CGTACGACCAAACCATCTCTGGATCTTGCAAAGGAACTGCTTAGCATGTTCACTGCAAGG 480

LOC_Os04g52450-NIL CGTACGACCAAACCATCTCTGGATCTTGCAAAGGAACTGCTTAGCATGTTCACTGCAAGG 480

LOC_Os04g52450-TQ CGTACGACCAAACCATCTCTGGATCTTGCAAAGGAACTGCTTAGCATGTTCACTGCAAGG 480

************************************************************

LOC_Os04g52450-LT GAAATGGGAAAAGTGTTCTTCACAAATAGTGGTTCAGAAGCAAATGATTCTCAGGTAAAA 540

LOC_Os04g52450-NIL GAAATGGGAAAAGTGTTCTTCACAAATAGTGGTTCAGAAGCAAATGATTCTCAGGTAAAA 540

LOC_Os04g52450-TQ GAAATGGGAAAAGTGTTCTTCACAAATAGTGGTTCAGAAGCAAATGATTCTCAGGTAAAA 540

************************************************************

LOC_Os04g52450-LT CTGGTCTGGTATTATAACAATGCACTGGGAAGGCCAGACAAGAAGAAATTTATTGCACGA 600

LOC_Os04g52450-NIL CTGGTCTGGTATTATAACAATGCACTGGGAAGGCCAGACAAGAAGAAATTTATTGCACGA 600

LOC_Os04g52450-TQ CTGGTCTGGTATTATAACAATGCACTGGGAAGGCCAGACAAGAAGAAATTTATTGCACGA 600

************************************************************

LOC_Os04g52450-LT TCAAAATCATACCATGGATCAACACTAATATCAGCTAGTCTATCCGGTCTTCCTGCACTG 660

LOC_Os04g52450-NIL TCAAAATCATACCATGGATCAACACTAATATCAGCTAGTCTATCCGGTCTTCCTGCACTG 660

LOC_Os04g52450-TQ TCAAAATCATACCATGGATCAACATTAATATCAGCTAGTCTATCCGGTCTTCCTGCACTG 660

************************:***********************************

LOC_Os04g52450-LT CATCAGAAGTTTGATCTGCCTGCACCTTTTGTTCTGCACACGGACTGCCCTCACTACTGG 720

LOC_Os04g52450-NIL CATCAGAAGTTTGATCTGCCTGCACCTTTTGTTCTGCACACGGACTGCCCTCACTACTGG 720

LOC_Os04g52450-TQ CATCAGAAGTTTGATCTGCCTGCACCTTTTGTTCTGCACACGGACTGCCCTCACTACTGG 720

************************************************************

LOC_Os04g52450-LT CGCTTCCATCTTCCTGGTGAAACAGAAGAAGAATTTGCAACTAGACTTGCCAATAATTTA 780

LOC_Os04g52450-NIL CGCTTCCATCTTCCTGGTGAAACAGAAGAAGAATTTGCAACTAGACTTGCCAATAATTTA 780

LOC_Os04g52450-TQ CGCTTCCATCTTCCTGGTGAAACAGAAGAAGAATTTGCAACTAGACTTGCCAATAATTTA 780

************************************************************

LOC_Os04g52450-LT GAGGAACTTATCCTCAAAGAAGGACCAGAAACAATTGCTGCATTCATTGCAGAGCCTGTG 840

LOC_Os04g52450-NIL GAGGAACTTATCCTCAAAGAAGGACCAGAAACAATTGCTGCATTCATTGCAGAGCCTGTG 840

LOC_Os04g52450-TQ GAGGAACTTATCCTCAAAGAAGGACCAGAAACAATTGCTGCATTCATTGCAGAGCCTGTG 840

************************************************************

LOC_Os04g52450-LT ATGGGTGCTGGTGGTGTCATACCTCCTCCAAAGACCTATTTTGAAAAGGTCCAAGCGATC 900

LOC_Os04g52450-NIL ATGGGTGCTGGTGGTGTCATACCTCCTCCAAAGACCTATTTTGAAAAGGTCCAAGCGATC 900

LOC_Os04g52450-TQ ATGGGTGCTGGTGGTGTCATACCTCCTCCAAAGACCTATTTTGAAAAGGTCCAAGCGATC 900

************************************************************

LOC_Os04g52450-LT GTTAAGAAGTATGACATCCTTTTCATAGCAGATGAGGTCATTACTGCATTTGGAAGGTTG 960

LOC_Os04g52450-NIL GTTAAGAAGTATGACATCCTTTTCATAGCAGATGAGGTCATTACTGCATTTGGAAGGTTG 960

LOC_Os04g52450-TQ GTTAAGAAGTATGACATCCTTTTCATAGCAGATGAGGTCATTACTGCATTTGGAAGGTTG 960

************************************************************

LOC_Os04g52450-LT GGAACCATGTTTGGAAGTGATATGTATAACATCAAGCCAGATCTAGTCTCCATGGCCAAG 1020

LOC_Os04g52450-NIL GGAACCATGTTTGGAAGTGATATGTATAACATCAAGCCAGATCTAGTCTCCATGGCCAAG 1020

LOC_Os04g52450-TQ GGAACCATGTTTGGAAGTGATATGTATAACATCAAGCCAGATCTAGTCTCCATGGCCAAG 1020

************************************************************

LOC_Os04g52450-LT GCGCTTTCATCTGCCTATGTGCCCATTGGAGCAATTATGGTTAGCCCAGAAATATCAGAT 1080

LOC_Os04g52450-NIL GCGCTTTCATCTGCCTATGTGCCCATTGGAGCAATTATGGTTAGCCCAGAAATATCAGAT 1080

LOC_Os04g52450-TQ GCGCTTTCATCTGCCTATGTGCCCATTGGAGCAATTATGGTTAGCCCAGAAATATCAGAT 1080

************************************************************

LOC_Os04g52450-LT GTTATTCATTCTCAGAGCAATAAGCTCGGTTCATTTGCTCATGGATTTACATACTCTGGC 1140

LOC_Os04g52450-NIL GTTATTCATTCTCAGAGCAATAAGCTCGGTTCATTTGCTCATGGATTTACATACTCTGGC 1140

LOC_Os04g52450-TQ GTTATTCATTCTCAGAGCAATAAGCTCGGTTCATTTGCTCATGGATTTACATACTCTGGC 1140

************************************************************

LOC_Os04g52450-LT CATCCAGTTGCATGTGCTGTCGCCATAGAAGCCCTGAAAATTTATCAGGAAAGGAACATT 1200

LOC_Os04g52450-NIL CATCCAGTTGCATGTGCTGTCGCCATAGAAGCCCTGAAAATTTATCAGGAAAGGAACATT 1200

LOC_Os04g52450-TQ CATCCAGTTGCATGTGCTGTCGCCATAGAAGCCCTGAAAATTTATCAGGAAAGGAACATT 1200

************************************************************

LOC_Os04g52450-LT CCTGATCATGTGAAGCAAATTTCTCCAAGGTTCCAGGAGGGAGTCAAGGCCTTTGCAGGA 1260

LOC_Os04g52450-NIL CCTGATCATGTGAAGCAAATTTCTCCAAGGTTCCAGGAGGGAGTCAAGGCCTTTGCAGGA 1260

LOC_Os04g52450-TQ CCTGATCATGTGAAGCAAATTTCTCCAAGGTTCCAGGAGGGAGTCAAGGCCTTTGCAGGA 1260

************************************************************

LOC_Os04g52450-LT AGTCCGATTGTTGGAGAGATACGTGGTGTAGGGTTGATACTTGGAACTGAATTCGCAGAC 1320

LOC_Os04g52450-NIL AGTCCGATTGTTGGAGAGATACGTGGTGTAGGGTTGATACTTGGAACTGAATTCGCAGAC 1320

LOC_Os04g52450-TQ AGTCCGATTGTTGGAGAGATACGTGGTGTAGGGTTGATACTTGGAACTGAATTCGCAGAC 1320

************************************************************

LOC_Os04g52450-LT AACAAATCGCCAAATGATCCATTCCCTGCTGAATGGGGCGTCGGCGCGATCTTTGGAGCG 1380

LOC_Os04g52450-NIL AACAAATCGCCAAATGATCCATTCCCTGCTGAATGGGGCGTCGGCGCGATCTTTGGAGCG 1380

LOC_Os04g52450-TQ AACAAATCGCCAAATGATCCATTCCCTGCTGAATGGGGCGTCGGCGCGATCTTTGGAGCG 1380

************************************************************

LOC_Os04g52450-LT GAGTGCCAGAAGCGCGGCATGCTGGTTAGAGTTGCTGGAGACAACATCATGATGTCACCA 1440

LOC_Os04g52450-NIL GAGTGCCAGAAGCGCGGCATGCTGGTTAGAGTTGCTGGAGACAACATCATGATGTCACCA 1440

LOC_Os04g52450-TQ GAGTGCCAGAAGCGCGGCATGCTGGTTAGAGTTGCTGGAGACAACATCATGATGTCACCA 1440

************************************************************

LOC_Os04g52450-LT CCATTGATAATGACCCCTGATGAAGTCGAGGAGCTGGTGAGCATCTATGGAGATGCACTC 1500

LOC_Os04g52450-NIL CCATTGATAATGACCCCTGATGAAGTCGAGGAGCTGGTGAGCATCTATGGAGATGCACTC 1500

LOC_Os04g52450-TQ CCATTGATAATGACCCCTGATGAAGTCGAGGAGCTGGTGAGCATCTATGGAGATGCACTC 1500

************************************************************

LOC_Os04g52450-LT AAGGCCACCGAGGAGAGGGTGGCTGAGCTGAAATCCAAGAAGAACAATTAG 1551

LOC_Os04g52450-NIL AAGGCCACCGAGGAGAGGGTGGCTGAGCTGAAATCCAAGAAGAACAATTAG 1551

LOC_Os04g52450-TQ AAGGCCACCGAGGAGAGGGTGGCTGAGCTGAAATCCAAGAAGAACAATTAG 1551

******************************************************
