# Supplementary material for: SS1 (NAL1)- and SS2-Mediated Genetic Networks Underlying Source-Sink and Yield Traits in Rice (Oryza sativa L.)
Source: PLoS One. 2015 Jul 10;10(7):e0132060. doi: 10.1371/journal.pone.0132060 (PMC4498882; doi:10.1371/journal.pone.0132060)
Supplement: S5 Fig — The gray regions indicate the coding region. The green regions indicate the trypsin-like serine and cysteine protease domain of NAL1 mutant. Underlined sequence indicates the deletion corresponding to the 10 amino acid. The red bars indicate the substitutions and deletions between LT, NIL-SS1 and TQ. Asterisks indicate complete homology; semicolons indicate substitution of DNA sequences; and spaces indicate complete lack of homology. Integers on the right indicate the cumulative number of nucleotide in the coding region [48]. (DOCX) [file pone.0132060.s005.docx]

LOC_Os04g52479-LT GAGTGCTCCGGGTGAGGTGAGGTGAGCGGCTCGAGATCCTGCCAAGATCAGCTCCTTGGC

LOC_Os04g52479-NIL GAGTGCTCCGGGTGAGGTGAGGTGAGCGGCTCGAGATCCTGCCAAGATCAGCTCCTTGGC

LOC_Os04g52479-TQ GAGTGCTCCGGGTGAGGTGAGGTGAGCGGCTCGAGATCCTGCCAAGATCAGCTCCTTGGC

************************************************************

LOC_Os04g52479-LT CTGGGGGTGGAGGACAGGTGGTATCTTGTGCCGTCTTTCTTGAGTTCTTGCGGTGGTTTT

LOC_Os04g52479-NIL CTGGGGGTGGAGGACAGGTGGTATCTTGTGCCGTCTTTCTTGAGTTCTTGCGGTGGTTTT

LOC_Os04g52479-TQ CTGGGGGTGGAGGACAGGTGGTATCTTGTGCCGTCTTTCTTGAGTTCTTGCGGTGGTTTT

************************************************************

LOC_Os04g52479-LT CTTTTTTTGAAGTTTGTTGGTTTCTTTTCTTCTTTTTTTTTTTGGGTTGGTTCGTGATTT

LOC_Os04g52479-NIL CTTTTTTTGAAGTTTGTTGGTTTCTTTTCTTCTTTTTTTTTTTGGGTTGGTTCGTGATTT

LOC_Os04g52479-TQ CTTTTTTTGAAGTTTGTTGGTTTCTTTTCTTCTTTTTTTTTTTGGGTTGGTTCGTGATTT

************************************************************

LOC_Os04g52479-LT TTTTGTTGTTGTGATGGCGGCTGAGTTGTTTGTTTAGTCTGTTCTTGATGTTTGCTTAGG

LOC_Os04g52479-NIL TTTTGTTGTTGTGATGGCGGCTGAGTTGTTTGTTTAGTCTGTTCTTGATGTTTGCTTAGG

LOC_Os04g52479-TQ TTTTGTTGTTGTGATGGCGGCTGAGTTGTTTGTTTAGTCTGTTCTTGATGTTTGCTTAGG

************************************************************

LOC_Os04g52479-LT GGGGGTACTGATGTGTGGGTAGGGTTCGGGTCCTTTTTTTTTTGCGTTTATCTTCCCTTT

LOC_Os04g52479-NIL GGGGGTACTGATGTGTGGGTAGGGTTCGGGTCCTTTTTTTTTTGCGTTTATCTTCCCTTT

LOC_Os04g52479-TQ GGGGGTACTGATGTGTGGGTAGGGTTCGGGTCCTTTTTTTTTTGCGTTTATCTTCCCTTT

************************************************************

LOC_Os04g52479-LT CCTTTTTTTTTTCTTCTTCCCCTTTTGAGATTTCGATCGTAGCATTGGTTAGCGTGTTGG

LOC_Os04g52479-NIL CCTTTTTTTTTTCTTCTTCCCCTTTTGAGATTTCGATCGTAGCATTGGTTAGCGTGTTGG

LOC_Os04g52479-TQ CCTTTTTTTTTTCTTCTTCCCCTTTTGAGATTTCGATCGTAGCATTGGTTAGCGTGTTGG

************************************************************

LOC_Os04g52479-LT TGGATGTAGTTAATCAATCCCCTTTTAGGTGCCAAATGTCATGGAAAGCGAGCTCTTTGC

LOC_Os04g52479-NIL TGGATGTAGTTAATCAATCCCCTTTTAGGTGCCAAATGTCATGGAAAGCGAGCTCTTTGC

LOC_Os04g52479-TQ TGGATGTAGTTAATCAATCCCCTTTTAGGTGCCAAATGTCATGGAAAGCGAGCTCTTTGC

************************************************************

LOC_Os04g52479-LT CCTTGTTTAACCAAGCTTTTGATCGCAGCATTCAAGAATTGGCTCCATCTCCCCGCAAAA

LOC_Os04g52479-NIL CCTTGTTTAACCAAGCTTTTGATCGCAGCATTCAAGAATTGGCTCCATCTCCCCGCAAAA

LOC_Os04g52479-TQ CCTTGTTTAACCAAGCTTTTGATCGCAGCATTCAAGAATTGGCTCCATCTCCCCGCAAAA

************************************************************

LOC_Os04g52479-LT AAAAAAAGAAAGAAAAAGGAATTGGCTCCATCGATTCAGTAAAAAAACCCGAATGCCGCG

LOC_Os04g52479-NIL AAAAAAAGAAAGAAAAAGGAATTGGCTCCATCGATTCAGTAAAAAAACCCGAATGCCGCG

LOC_Os04g52479-TQ AAAAAAAGAAAGAAAAAGGAATTGGCTCCATCGATTCAGTAAAAAAACCCGAATGCCGCG

************************************************************

LOC_Os04g52479-LT GAAGTTAGCATTGCTCATCCGGGGATTCGACGCTGGCACACTGGTTTCTTGGCTAGAGAG

LOC_Os04g52479-NIL GAAGTTAGCATTGCTCATCCGGGGATTCGACGCTGGCACACTGGTTTCTTGGCTAGAGAG

LOC_Os04g52479-TQ GAAGTTAGCATTGCTCATCCGGGGATTCGACGCTGGCACACTGGTTTCTTGGCTAGAGAG

************************************************************

LOC_Os04g52479-LT AAACAACGATGTGCTTGCTGTTTCACCCACCAATTTCTTCCCGGCTTGGGATTTTTGCCT

LOC_Os04g52479-NIL AAACAACGATGTGCTTGCTGTTTCACCCACCAATTTCTTCCCGGCTTGGGATTTTTGCCT

LOC_Os04g52479-TQ AAACAACGATGTGCTTGCTGTTTCACCCACCAATTTCTTCCCGGCTTGGGATTTTTGCCT

************************************************************

LOC_Os04g52479-LT TTTGTATCTGCTTTCTTTTTCCTCTTTTGAAAAAGTACACGATTCCTTTGCCTACCTTCA

LOC_Os04g52479-NIL TTTGTATCTGCTTTCTTTTTCCTCTTTTGAAAAAGTACACGATTCCTTTGCCTACCTTCA

LOC_Os04g52479-TQ TTTGTATCTGCTTTCTTTTTCCTCTTTTGAAAAAGTACACGATTCCTTTGCCTACCTTCA

************************************************************

LOC_Os04g52479-LT TTTTCAGTGCCGATTACTAAATTTGCAGTAGGTCCTACTCTCTCGCCGTGGTGTTGGACT

LOC_Os04g52479-NIL TTTTCAGTGCCGATTACTAAATTTGCAGTAGGTCCTACTCTCTCGCCGTGGTGTTGGACT

LOC_Os04g52479-TQ TTTTCAGTGCCGATTACTAAATTTGCAGTAGGTCCTACTCTCTCGCCGTGGTGTTGGACT

************************************************************

LOC_Os04g52479-LT ATCATTGGGAGTAGTTGTCATGGGGATTTATGCCCATTTGCTGCGATTAAAGAGAGAATA

LOC_Os04g52479-NIL ATCATTGGGAGTAGTTGTCATGGGGATTTATGCCCATTTGCTGCGATTAAAGAGAGAATA

LOC_Os04g52479-TQ ATCATTGGGAGTAGTTGTCATGGGGATTTATGCCCATTTGCTGCGATTAAAGAGAGAATA

************************************************************

LOC_Os04g52479-LT TGTGCTACGCCTTAACGTTCATGGACCTGATTTCTTGAGGTCGCTGTAACTGTTGCCTTT

LOC_Os04g52479-NIL TGTGCTACGCCTTAACGTTCATGGACCTGATTTCTTGAGGTCGCTGTAACTGTTGCCTTT

LOC_Os04g52479-TQ TGTGCTACGCCTTAACGTTCATGGACCTGATTTCTTGAGGTCGCTGTAACTGTTGCCTTT

************************************************************

LOC_Os04g52479-LT GGAGAACAAGAGGAAGAGGGTGTGAGTGTGTGGAGACACTAACCGTGGCTTTTGCAGATT

LOC_Os04g52479-NIL GGAGAACAAGAGGAAGAGGGTGTGAGTGTGTGGAGACACTAACCGTGGCTTTTGCAGATT

LOC_Os04g52479-TQ GGAGAACAAGAGGAAGAGGGTGTGAGTGTGTGGAGACACTAACCGTGGCTTTTGCAGATT

************************************************************

LOC_Os04g52479-LT CCTCTTATCTGTTTTCCTTGCATGGCAGTTGTGCCATACATTGATACAGATGGATCATAT

LOC_Os04g52479-NIL CCTCTTATCTGTTTTCCTTGCATGGCAGTTGTGCCATACATTGATACAGATGGATCATAT

LOC_Os04g52479-TQ CCTCTTATCTGTTTTCCTTGCATGGCAGTTGTGCCATACATTGATACAGATGGATCATAT

************************************************************

LOC_Os04g52479-LT TCTTCGTACAACCGGATTGGAGTATGCTCGATCTGTCATCCGTGGAGAACCCGAAATTCA

LOC_Os04g52479-NIL TCTTCGTACAACCGGATTGGAGTATGCTCGATCTGTCATCCGTGGAGAACCCGAAATTCA

LOC_Os04g52479-TQ TCTTCGTACAACCGGATTGGAGTATGCTCGATCTGTCATCCGTGGAGAACCCGAAATTCA

************************************************************

LOC_Os04g52479-LT GATTGCTTTTGTTTTGCGTGGCTCTCCTTTGCAGTTCTTGCGGGAACCTATTCCCCCAAG

LOC_Os04g52479-NIL GATTGCTTTTGTTTTGCGTGGCTCTCCTTTGCAGTTCTTGCGGGAACCTATTCCCCCAAG

LOC_Os04g52479-TQ GATTGCTTTTGTTTTGCGTGGCTCTCCTTTGCAGTTCTTGCGGGAACCTATTCCCCCAAG

************************************************************

LOC_Os04g52479-LT AATATCATGCTACTCCACATCTTGTTGTTTATTCTACTGATTCCATCTTTTCGTTCGGGC

LOC_Os04g52479-NIL AATATCATGCTACTCCACATCTTGTTGTTTATTCTACTGATTCCATCTTTTCGTTCGGGC

LOC_Os04g52479-TQ AATATCATGCTACTCCACATCTTGTTGTTTATTCTACTGATTCCATCTTTTCGTTCGGGC

************************************************************

LOC_Os04g52479-LT CAGGCCAGCTAGCTAGCCGCAAGCGCTGACTGTCTTGATCATTGATTCCTCCTTCCACAA

LOC_Os04g52479-NIL CAGGCCAGCTAGCTAGCCGCAAGCGCTGACTGTCTTGATCATTGATTCCTCCTTCCACAA

LOC_Os04g52479-TQ CAGGCCAGCTAGCTAGCCGCAAGCGCTGACTGTCTTGATCATTGATTCCTCCTTCCACAA

************************************************************

LOC_Os04g52479-LT TAACTCTAAAAGATTGGAAGTACATTTGCATGATTGATGGTTTTCCCGTCGCTTTCGGCA

LOC_Os04g52479-NIL TAACTCTAAAAGATTGGAAGTACATTTGCATGATTGATGGTTTTCCCGTCGCTTTCGGCA

LOC_Os04g52479-TQ TAACTCTAAAAGATTGGAAGCACATTTGCATGATTGATGGTTTTCCCGTCGCTTTCGGCA

********************:***************************************

LOC_Os04g52479-LT TTCGTTATCTACCTGTCCATTAGCCTTCAGGATCATGCTTTCTGACTTGCTTGTTCTCAT

LOC_Os04g52479-NIL TTCGTTATCTACCTGTCCATTAGCCTTCAGGATCATGCTTTCTGACTTGCTTGTTCTCAT

LOC_Os04g52479-TQ TTCGTTATCTACCTGTCCATTAGCCTTCAGGATCATGCTTTCTGACTTGCTTGTTCTCAT

************************************************************

LOC_Os04g52479-LT TCTTAGGGCCATAACTTCAGCTTCTCCCATCTATAATAGGTTCGCAAACTGTTCAGCACA

LOC_Os04g52479-NIL TCTTAGGGCCATAACTTCAGCTTCTCCCATCTATAATAGGTTCGCAAACTGTTCAGCACA

LOC_Os04g52479-TQ TCTTAGGGCCATAACTTCAGCTTCTCCCATCTATAATAGGTTCGCAAACTGTTCAGCACA

************************************************************

LOC_Os04g52479-LT ATGAAGCCTTCGGACGATAAGGCGCAGCTCTCCGGTTTGGCGCAATCAGAAGAATCGTCA 60

LOC_Os04g52479-NIL ATGAAGCCTTCGGACGATAAGGCGCAGCTCTCCGGTTTGGCGCAATCAGAAGAATCGTCA 60

LOC_Os04g52479-TQ ATGAAGCCTTCGGACGATAAGGCGCAGCTCTCCGGTTTGGCGCAATCAGAAGAATCGTCA 60

************************************************************

LOC_Os04g52479-LT CTTGATGTGGATCACCAGTCATTTCCTTGTTCTCCATCAATCCAACCGGTTGCTTCTGGG 120

LOC_Os04g52479-NIL CTTGATGTGGATCACCAGTCATTTCCTTGTTCTCCATCAATCCAACCGGTTGCTTCTGGG 120

LOC_Os04g52479-TQ CTTGATGTGGATCACCAGTCATTTCCTTGTTCTCCATCAATCCAACCGGTTGCTTCTGGG 120

************************************************************

LOC_Os04g52479-LT TGCACACACACAGAGAACAGCGCAGCATACTTCTTATGGCCGACATCCAACCTACAGCAT 180

LOC_Os04g52479-NIL TGCACACACACAGAGAACAGCGCAGCATACTTCTTATGGCCGACATCCAACCTACAGCAT 180

LOC_Os04g52479-TQ TGCACACACACAGAGAACAGCGCAGCATACTTCTTATGGCCGACATCCAACCTACAGCAT 180

************************************************************

LOC_Os04g52479-LT TGTGCAGCCGAGGGACGTGCAAACTACTTTGGAAACCTTCAGAAAGGATTGTTGCCAAGG 240

LOC_Os04g52479-NIL TGTGCAGCCGAGGGACGTGCAAACTACTTTGGAAACCTTCAGAAAGGATTGTTGCCAAGG 240

LOC_Os04g52479-TQ TGTGCAGCCGAGGGACGTGCAAACTACTTTGGAAACCTTCAGAAAGGATTGTTGCCAAGG 240

************************************************************

LOC_Os04g52479-LT CACCCTGGTCGGTTGCCCAAAGGTCAGCAAGCAAATAGCTTGCTTGACTTGATGACTATA 300

LOC_Os04g52479-NIL CACCCTGGTCGGTTGCCCAAAGGTCAGCAAGCAAATAGCTTGCTTGACTTGATGACTATA 300

LOC_Os04g52479-TQ CACCCTGGTCGGTTGCCCAAAGGTCAGCAAGCAAATAGCTTGCTTGACTTGATGACTATA 300

************************************************************

LOC_Os04g52479-LT AGAGCTTTCCATAGCAAGATATTGCGGCGTTTTAGCCTCGGGACAGCAGTGGGATTCCGC 360

LOC_Os04g52479-NIL AGAGCTTTCCATAGCAAGATATTGCGGCGTTTTAGCCTCGGGACAGCAGTGGGATTCCGC 360

LOC_Os04g52479-TQ AGAGCTTTCCATAGCAAGATATTGCGGCGTTTTAGCCTCGGGACAGCAGTGGGATTCCGC 360

************************************************************

LOC_Os04g52479-LT ATCAGGAAAGGGGATCTAACAGATATCCCTGCAATCCTTGTCTTTGTTGCTCGCAAGGTT 420

LOC_Os04g52479-NIL ATCAGGAAAGGGGATCTAACAGATATCCCTGCAATCCTTGTCTTTGTTGCTCGCAAGGTT 420

LOC_Os04g52479-TQ ATCAGGAAAGGGGATCTAACAGATATCCCTGCAATCCTTGTCTTTGTTGCTCGCAAGGTT 420

************************************************************

LOC_Os04g52479-LT CATAAGAAGTGGCTTAATCCAGCACAATGTCTTCCTGCTATTCTTGAGGGTCCAGGAGGT 480

LOC_Os04g52479-NIL CATAAGAAGTGGCTTAATCCAGCACAATGTCTTCCTGCTATTCTTGAGGGTCCAGGAGGT 480

LOC_Os04g52479-TQ CATAAGAAGTGGCTTAATCCAGCACAATGTCTTCCTGCTATTCTTGAGGGTCCAGGAGGT 480

************************************************************

LOC_Os04g52479-LT GTTTGGTGTGATGTTGATGTTGTTGAATTTTCGTACTACGGTGCACCGGCTCAAACACCT 540

LOC_Os04g52479-NIL GTTTGGTGTGATGTTGATGTTGTTGAATTTTCGTACTACGGTGCACCGGCTCAAACACCT 540

LOC_Os04g52479-TQ GTTTGGTGTGATGTTGATGTTGTTGAATTTTCGTACTACGGTGCACCGGCTCAAACACCT 540

************************************************************

LOC_Os04g52479-LT AAAGAGCAAATGTTCAGTGAGCTTGTTGATAAGTTATGTGGCAGTGACGAATGTATTGGT 600

LOC_Os04g52479-NIL AAAGAGCAAATGTTCAGTGAGCTTGTTGATAAGTTATGTGGCAGTGACGAATGTATTGGT 600

LOC_Os04g52479-TQ AAAGAGCAAATGTTCAGTGAGCTTGTTGATAAGTTATGTGGCAGTGACGAATGTATTGGT 600

************************************************************

LOC_Os04g52479-LT TCAGGCTCTCAGGTTGCAAGCCATGAAACTTTTGGTACTTTGGGTGCAATTGTGAAACGG 660

LOC_Os04g52479-NIL TCAGGCTCTCAGGTTGCAAGCCATGAAACTTTTGGTACTTTGGGTGCAATTGTGAAACGG 660

LOC_Os04g52479-TQ TCAGGCTCTCAGGTTGCAAGCCATGAAACTTTTGGTACTTTGGGTGCAATTGTGAAACGG 660

************************************************************

LOC_Os04g52479-LT CGCACTGGCAACAAGCAGGTTGGTTTCCTCACTAACCATCATGTCGCGGTTGACTTGGAC 720

LOC_Os04g52479-NIL CGCACTGGCAACAAGCAGGTTGGTTTCCTCACTAACCATCATGTCGCGGTTGACTTGGAC 720

LOC_Os04g52479-TQ CGCACTGGCAACAAGCAGGTTGGTTTCCTCACTAACCGTCATGTCGCGGTTGACTTGGAC 720

*************************************:**********************

LOC_Os04g52479-LT TACCCTAATCAGAAGATGTTTCATCCATTACCACCCAATCTTGGGCCTGGCGTTTATCTT 780

LOC_Os04g52479-NIL TACCCTAATCAGAAGATGTTTCATCCATTACCACCCAATCTTGGGCCTGGCGTTTATCTT 780

LOC_Os04g52479-TQ TACCCTAATCAGAAGATGTTTCATCCATTACCACCCAATCTTGGGCCTGGCGTTTATCTT 780

************************************************************

LOC_Os04g52479-LT GGAGCTGTTGAAAGAGCAACTTCTTTCATCACAGATGACGTTTGGTATGGAATCTATGCT 840

LOC_Os04g52479-NIL GGAGCTGTTGAAAGAGCAACTTCTTTCATCACAGATGACGTTTGGTATGGAATCTATGCT 840

LOC_Os04g52479-TQ GGAGCTGTTGAAAGAGCAACTTCTTTCATCACAGATGACGTTTGGTATGGAATCTATGCT 840

************************************************************

LOC_Os04g52479-LT GGAACAAACCCAGAGACATTTGTACGAGCTGACGGTGCATTTATCCCATTTGCTGATGAC 900

LOC_Os04g52479-NIL GGAACAAACCCAGAGACATTTGTACGAGCTGACGGTGCATTTATCCCATTTGCTGATGAC 900

LOC_Os04g52479-TQ GGAACAAACCCAGAGACATTTGTACGAGCTGACGGTGCATTTATCCCATTTGCTGATGAC 900

************************************************************

LOC_Os04g52479-LT TTTGACATTTCCACCGTCACGACTGTAGTTAGGGGAGTCGGTGACATTGGGGATGTCAAG 960

LOC_Os04g52479-NIL TTTGACATTTCCACCGTCACGACTGTAGTTAGGGGAGTCGGTGACATTGGGGATGTCAAG 960

LOC_Os04g52479-TQ TTTGACATTTCCACCGTCACGACTGTAGTTAGGGGAGTCGGTGACATTGGGGATGTCAAG 960

************************************************************

LOC_Os04g52479-LT GTTATAGATCTGCAGTGTCCGCTCAATAGCCTCATAGGGAGGCAAGTATGCAAAGTTGGC 1020

LOC_Os04g52479-NIL GTTATAGATCTGCAGTGTCCGCTCAATAGCCTCATAGGGAGGCAAGTATGCAAAGTTGGC 1020

LOC_Os04g52479-TQ GTTATAGATCTGCAGTGTCCGCTCAATAGCCTCATAGGGAGGCAAGTATGCAAAGTTGGC 1020

************************************************************

LOC_Os04g52479-LT AGAAGCTCTGGTCACACAACTGGGACTGTGATGGCCTATGCCCTTGAGTACAATGACGAG 1080

LOC_Os04g52479-NIL AGAAGCTCTGGTCACACAACTGGGACTGTGATGGCCTATGCCCTTGAGTACAATGACGAG 1080

LOC_Os04g52479-TQ AGAAGCTCTGGTCACACAACTGGGACTGTGATGGCCTATGCCCTTGAGTACAATGACGAG 1080

************************************************************

LOC_Os04g52479-LT AAAGGAATATGCTTCTTCACAGACATCCTTGTTGTTGGTGAGAACCGCCAAACATTTGAT 1140

LOC_Os04g52479-NIL AAAGGAATATGCTTCTTCACAGACATCCTTGTTGTTGGTGAGAACCGCCAAACATTTGAT 1140

LOC_Os04g52479-TQ AAAGGAATATGCTTCTTCACAGACATCCTTGTTGTTGGTGAGAACCGCCAAACATTTGAT 1140

************************************************************

LOC_Os04g52479-LT TTGGAAGGTGATAGCGGAAGCCTTATTATCCTGACTAGCCAAGATGGTGAGAAGCCGCGT 1200

LOC_Os04g52479-NIL TTGGAAGGTGATAGCGGAAGCCTTATTATCCTGACTAGCCAAGATGGTGAGAAGCCGCGT 1200

LOC_Os04g52479-TQ TTGGAAGGTGATAGCGGAAGCCTTATTATCCTGACTAGCCAAGATGGTGAGAAGCCGCGT 1200

************************************************************

LOC_Os04g52479-LT CCAATTGGAATTATATGGGGTGGCACAGCAAATCGTGGGAGGTTGAAGCTTACAAGTGAT 1260

LOC_Os04g52479-NIL CCAATTGGAATTATATGGGGTGGCACAGCAAATCGTGGGAGGTTGAAGCTTACAAGTGAT 1260

LOC_Os04g52479-TQ CCAATTGGAATTATATGGGGTGGCACAGCAAATCGTGGGAGGTTGAAGCTTACAAGTGAT 1260

************************************************************

LOC_Os04g52479-LT CATGGCCCTGAAAACTGGACTAGTGGGGTTGATCTTGGCCGTCTACTCGACCGTCTGGAA 1320

LOC_Os04g52479-NIL CATGGCCCTGAAAACTGGACTAGTGGGGTTGATCTTGGCCGTCTACTCGACCGTCTGGAA 1320

LOC_Os04g52479-TQ CATGGCCCTGAAAACTGGACTAGTGGGGTTGATCTTGGCCGTCTACTCGACCGTCTGGAA 1320

************************************************************

LOC_Os04g52479-LT CTTGATATTATCATTACCAATGAATCACTCCAAGATGCCGTGCAGCAGCAAAGATTTGCT 1380

LOC_Os04g52479-NIL CTTGATATTATCATTACCAATGAATCACTCCAAGATGCCGTGCAGCAGCAAAGATTTGCT 1380

LOC_Os04g52479-TQ CTTGATATTATCATTACCAATGAATCACTCCAAGATGCCGTGCAGCAGCAAAGATTTGCT 1380

************************************************************

LOC_Os04g52479-LT TTGGTGGCCGCCGTTACCTCAGCTGTTGGGGAGTCTTCCGGGGTGCCTGTCGCCATCCCG 1440

LOC_Os04g52479-NIL TTGGTGGCCGCCGTTACCTCAGCTGTTGGGGAGTCTTCCGGGGTGCCTGTCGCCATCCCG 1440

LOC_Os04g52479-TQ TTGGTGGCCGCCGTTACCTCAGCTGTTGGGGAGTCTTCCGGGGTGCCTGTCGCCATCCCG 1440

************************************************************

LOC_Os04g52479-LT GAAGAGAAGATCGAAGAGATCTTCGAGCCATTGGGGATCCAAATCCAGCAACTGCCTCGC 1500

LOC_Os04g52479-NIL GAAGAGAAGATCGAAGAGATCTTCGAGCCATTGGGGATCCAAATCCAGCAACTGCCTCGC 1500

LOC_Os04g52479-TQ GAAGAGAAGATCGAAGAGATCTTCGAGCCATTGGGGATCCAAATCCAGCAACTGCCTCGC 1500

************************************************************

LOC_Os04g52479-LT CATGACGTGGCGGCCTCTGGAACTGAAGGGGAGGAGGCATCCAACACGGTGGTCAATGTG 1560

LOC_Os04g52479-NIL CATGACGTGGCGGCCTCTGGAACTGAAGGGGAGGAGGCATCCAACACGGTGGTCAATGTG 1560

LOC_Os04g52479-TQ CATGACGTGGCGGCCTCTGGAACTGAAGGGGAGGAGGCATCCAACACGGTGGTCAATGTG 1560

************************************************************

LOC_Os04g52479-LT GAAGAGCACCAGTTCATCTCAAACTTCGTCGGTATGTCGCCCGTGCGCGACGACCAAGAC 1620

LOC_Os04g52479-NIL GAAGAGCACCAGTTCATCTCAAACTTCGTCGGTATGTCGCCCGTGCGCGACGACCAAGAC 1620

LOC_Os04g52479-TQ GAAGAGCACCAGTTCATCTCAAACTTCGTCGGTATGTCGCCCGTGCGCGACGACCAAGAC 1620

************************************************************

LOC_Os04g52479-LT GCTCCGAGGAGCATCACCAACCTGAACAACCCCTCCGAGGAAGAACTCGCCATGTCGCTC 1680

LOC_Os04g52479-NIL GCTCCGAGGAGCATCACCAACCTGAACAACCCCTCCGAGGAAGAACTCGCCATGTCGCTC 1680

LOC_Os04g52479-TQ GCTCCGAGGAGCATCACCAACCTGAACAACCCCTCCGAGGAAGAACTCGCCATGTCGCTC 1680

************************************************************

LOC_Os04g52479-LT CATCTGGGTGACCGAGAGCCCAAGCGGCTCCGTTCGGACTCCGGATCAAGCCTTGACCTG 1740

LOC_Os04g52479-NIL CATCTGGGTGACCGAGAGCCCAAGCGGCTCCGTTCGGACTCCGGATCAAGCCTTGACCTG 1740

LOC_Os04g52479-TQ CATCTGGGTGACCGAGAGCCCAAGCGGCTCCGTTCGGACTCCGGATCAAGCCTTGACCTG 1740

************************************************************

LOC_Os04g52479-LT GAGAAATGA 1749

LOC_Os04g52479-NIL GAGAAATGA 1749

LOC_Os04g52479-TQ GAGAAATGA 1749

*********
