# Supplementary material for: SS1 (NAL1)- and SS2-Mediated Genetic Networks Underlying Source-Sink and Yield Traits in Rice (Oryza sativa L.)
Source: PLoS One. 2015 Jul 10;10(7):e0132060. doi: 10.1371/journal.pone.0132060 (PMC4498882; doi:10.1371/journal.pone.0132060)
Supplement: S6 Fig — (PPTX) [file pone.0132060.s006.pptx]

## Slide 1
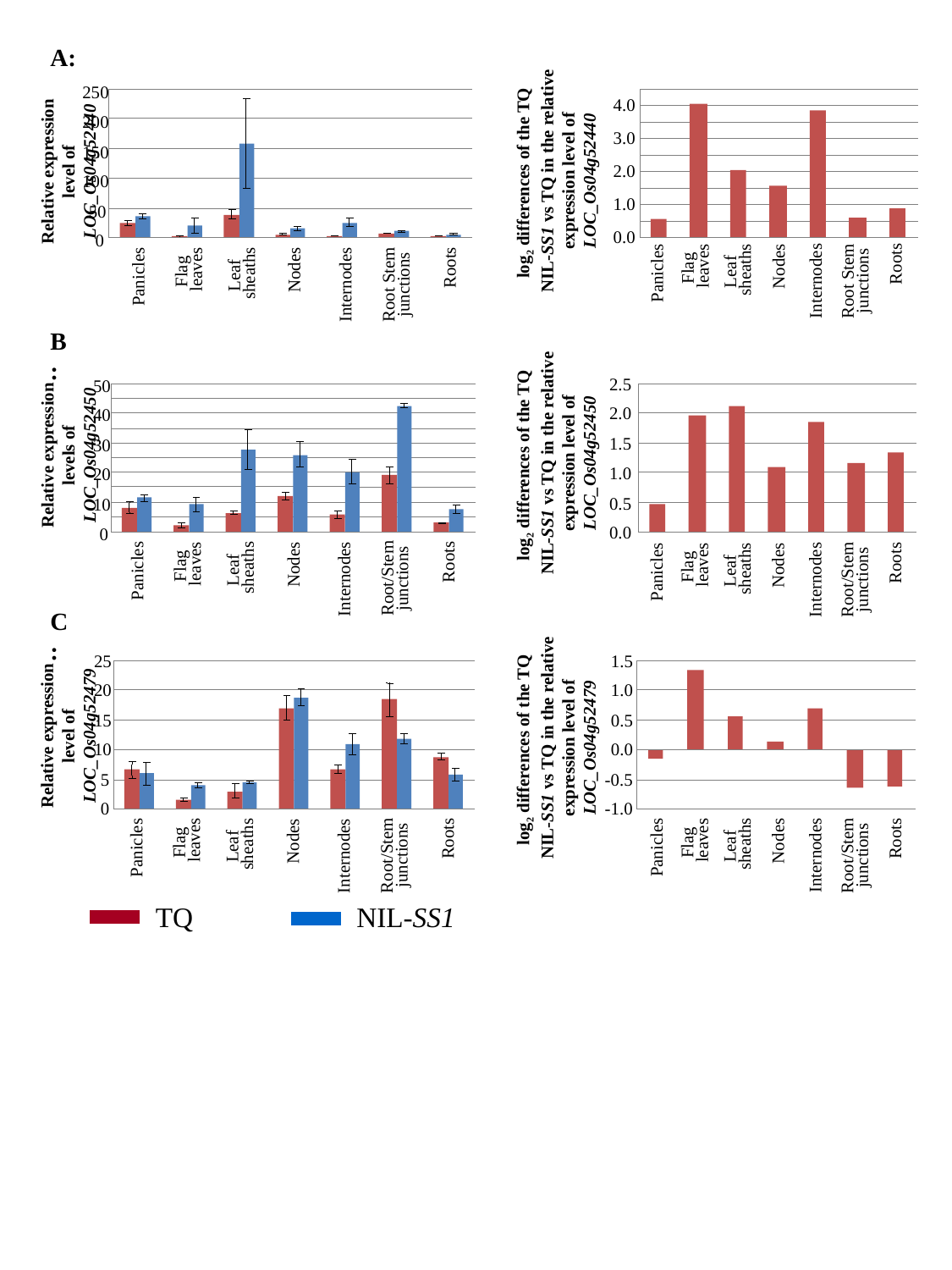

A:
250
200
150
Relative expression level of LOC_Os04g52440
100
50
0
Flag
leaves
Roots
Leaf
sheaths
Nodes
Panicles
Root Stem
junctions
Internodes
B:
50
40
30
20
10
0
Relative expression levels of LOC_Os04g52450
Flag
leaves
Roots
Leaf
sheaths
Nodes
Panicles
Root/Stem
junctions
Internodes
C:
25
20
15
10
5
0
Relative expression level of LOC_Os04g52479
Flag
leaves
Roots
Leaf
sheaths
Nodes
Panicles
Root/Stem
junctions
Internodes
4.0
3.0
log2 differences of the TQ NIL-SS1 vs TQ in the relative expression level of LOC_Os04g52440
2.0
1.0
0.0
Flag
leaves
Roots
Leaf
sheaths
Nodes
Panicles
Root Stem
junctions
Internodes
2.5
2.0
log2 differences of the TQ NIL-SS1 vs TQ in the relative expression level of LOC_Os04g52450
1.5
1.0
0.5
0.0
Flag
leaves
Roots
Leaf
sheaths
Nodes
Panicles
Root/Stem
junctions
Internodes
1.5
1.0
log2 differences of the TQ NIL-SS1 vs TQ in the relative expression level of LOC_Os04g52479
0.5
0.0
-0.5
-1.0
Flag
leaves
Roots
Leaf
sheaths
Nodes
Panicles
Root/Stem
junctions
Internodes
TQ
NIL-SS1
