# Supplementary figures and images for: SS1 (NAL1)- and SS2-Mediated Genetic Networks Underlying Source-Sink and Yield Traits in Rice (Oryza sativa L.)
Source: PLoS One. 2015 Jul 10;10(7):e0132060. doi: 10.1371/journal.pone.0132060 (PMC4498882; doi:10.1371/journal.pone.0132060)

## Slide 1
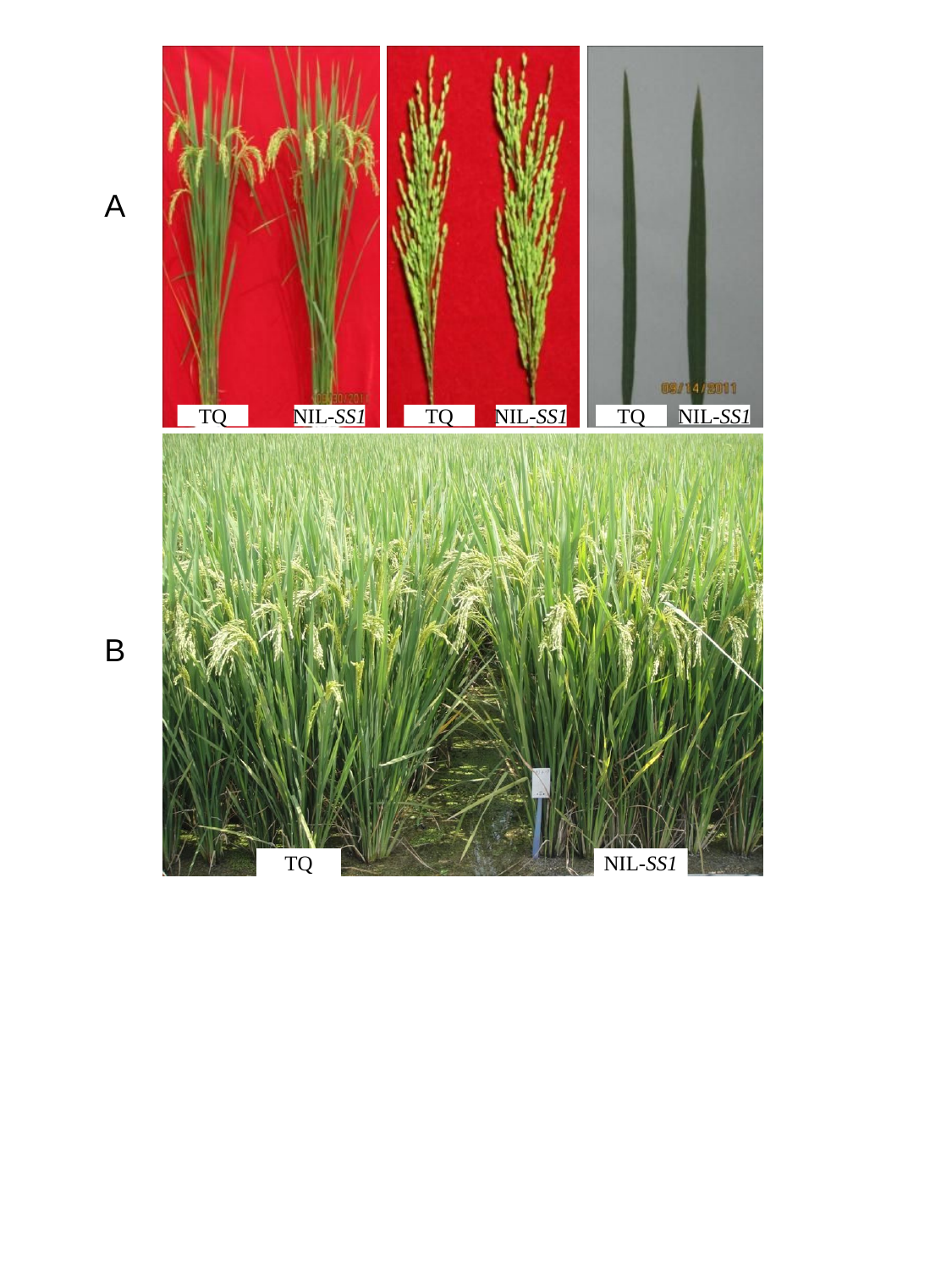

TQ
NIL-SS1
TQ
NIL-SS1
TQ
NIL-SS1
TQ
NIL-SS1
A
B

Supplement: S7 Fig — (A) Plant morphologies (left), panicle type (middle), and flag leaves (right); (B) Population performances of TQ (left) and NIL-SS1 (right) in 2012 summer season in Beijing. (PPTX) [file pone.0132060.s007.pptx]
