# Supplementary material for: SS1 (NAL1)- and SS2-Mediated Genetic Networks Underlying Source-Sink and Yield Traits in Rice (Oryza sativa L.)
Source: PLoS One. 2015 Jul 10;10(7):e0132060. doi: 10.1371/journal.pone.0132060 (PMC4498882; doi:10.1371/journal.pone.0132060)
Supplement: S8 Fig — (PPTX) [file pone.0132060.s008.pptx]

## Slide 1
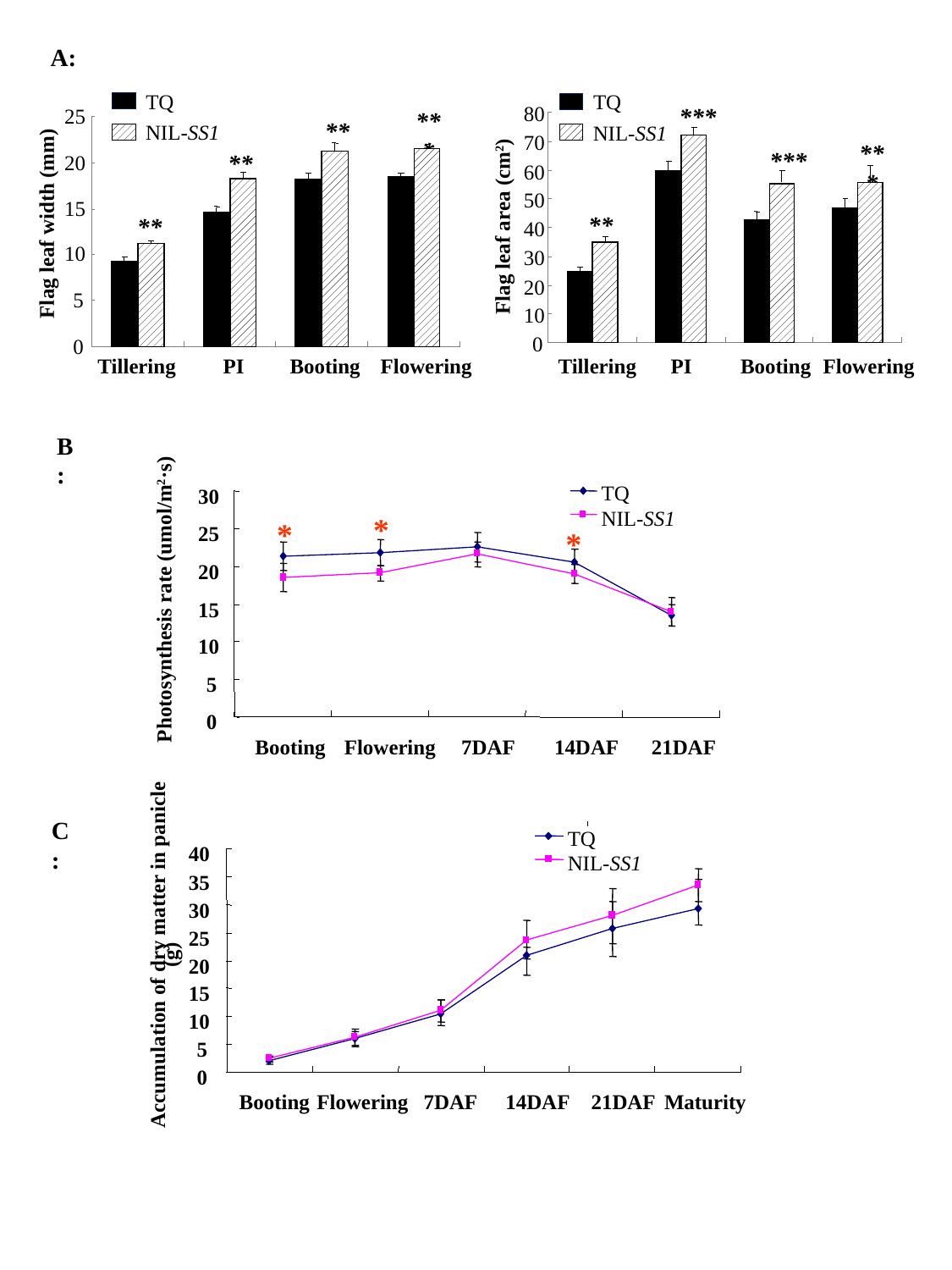

A:
TQ
TQ
***
***
80
25
***
NIL-SS1
NIL-SS1
70
***
***
***
20
60
50
15
***
***
Flag leaf area (cm2)
Flag leaf width (mm)
40
10
30
20
5
10
0
0
Tillering
PI
Booting
Flowering
Tillering
PI
Booting
Flowering
B:
TQ
30
*
NIL-SS1
*
*
25
20
Photosynthesis rate (umol/m2·s)
15
10
5
0
Booting
Flowering
7DAF
14DAF
21DAF
C:
TQ
40
NIL-SS1
35
30
25
Accumulation of dry matter in panicle (g)
20
15
10
5
0
Booting
Flowering
7DAF
14DAF
21DAF
Maturity

## Slide 2
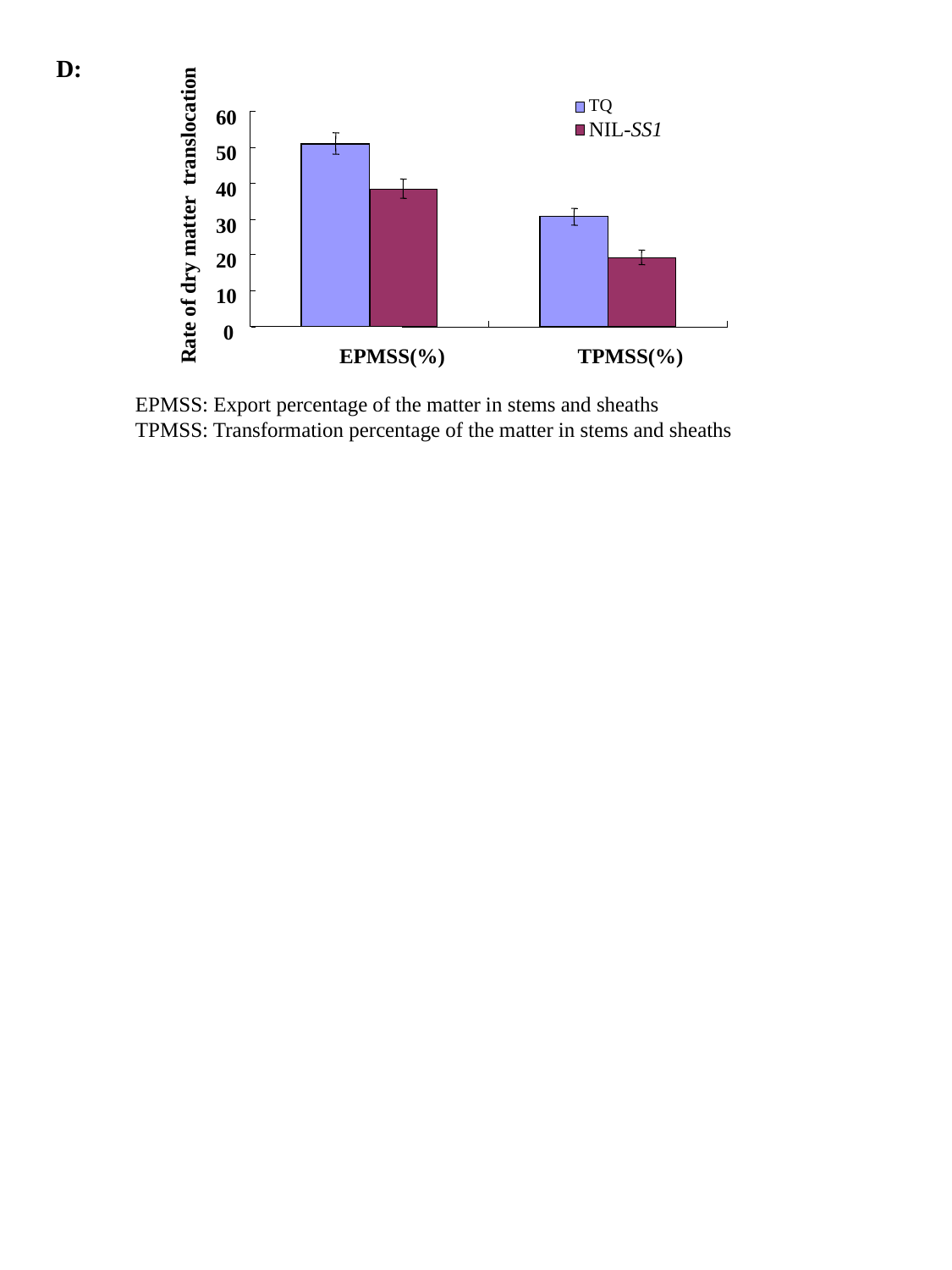

D:
TQ
60
NIL-SS1
50
40
Rate of dry matter translocation
30
20
10
0
EPMSS(%)
TPMSS(%)
EPMSS: Export percentage of the matter in stems and sheaths
TPMSS: Transformation percentage of the matter in stems and sheaths
