# Supplementary material for: SS1 (NAL1)- and SS2-Mediated Genetic Networks Underlying Source-Sink and Yield Traits in Rice (Oryza sativa L.)
Source: PLoS One. 2015 Jul 10;10(7):e0132060. doi: 10.1371/journal.pone.0132060 (PMC4498882; doi:10.1371/journal.pone.0132060)
Supplement: S1 Table — (DOC) [file pone.0132060.s009.doc]

**S1 Table** The SSR (RM) and SNP (Os) marker and bin information used for the linkage map construction and QTL analyses in the reciprocal sets of introgression populations of the LT/TQ cross

| Marker | Chr. | Physical distance (bp) | Bin | Marker | Chr. | Physical distance (bp) | Bin |
| --- | --- | --- | --- | --- | --- | --- | --- |
| Os01-554916 | 1 | 554916 | 1,1 | Os07-536980 | 7 | 536980 | 7,1 |
| Os01-1536267 | 1 | 1536267 | 1,1 | Os07-1536334 | 7 | 1536334 | 7,1 |
| Os01-2569238 | 1 | 2569238 | 1,1 | Os07-2456742 | 7 | 2456742 | 7,1 |
| Os01-3638070 | 1 | 3638070 | 1,2 | RM436 | 7 | 2583063 | 7,1 |
| RM84 | 1 | 3942440 | 1,2 | RM481 | 7 | 2907782 | 7,1 |
| Os01-4582766 | 1 | 4582766 | 1,2 | Os07-4473492 | 7 | 4473492 | 7,2 |
| OSR2 | 1 | 5556358 | 1,2 | Os07-5485802 | 7 | 5485802 | 7,2 |
| Os01-5726977 | 1 | 5726977 | 1,2 | Os07-6775896 | 7 | 6775896 | 7,2 |
| Os01-6642891 | 1 | 6642891 | 1,3 | Os07-7321600 | 7 | 7321600 | 7,2 |
| RM259 | 1 | 7443751 | 1,3 | Os07-8393665 | 7 | 8393665 | 7,3 |
| Os01-7739785 | 1 | 7739785 | 1,3 | Os07-9252371 | 7 | 9252371 | 7,3 |
| RM579 | 1 | 8449360 | 1,3 | Os07-10263906 | 7 | 10263906 | 7,3 |
| Os01-8790450 | 1 | 8790450 | 1,3 | Os07-11226221 | 7 | 11226221 | 7,4 |
| Os01-9765185 | 1 | 9765185 | 1,3 | Os07-12296396 | 7 | 12296396 | 7,4 |
| RM23 | 1 | 10702396 | 1,3 | RM214 | 7 | 12731768 | 7,4 |
| Os01-10822085 | 1 | 10822085 | 1,3 | Os07-13366226 | 7 | 13366226 | 7,4 |
| Os01-11803397 | 1 | 11803397 | 1,3 | Os07-14563822 | 7 | 14563822 | 7,4 |
| Os01-12663812 | 1 | 12663812 | 1,4 | Os07-15589945 | 7 | 15589945 | 7,4 |
| Os01-13717303 | 1 | 13717303 | 1,4 | Os07-16473785 | 7 | 16473785 | 7,5 |
| Os01-14748859 | 1 | 14748859 | 1,4 | RM445 | 7 | 17410787 | 7,5 |
| Os01-15777911 | 1 | 15777911 | 1,4 | Os07-18479683 | 7 | 18479683 | 7,5 |
| Os01-16493161 | 1 | 16493161 | 1,5 | RM11 | 7 | 19205640 | 7,5 |
| Os01-17500259 | 1 | 17500259 | 1,5 | Os07-19378516 | 7 | 19378516 | 7,5 |
| RM129 | 1 | 18786142 | 1,5 | Os07-20352240 | 7 | 20352240 | 7,5 |
| Os01-19516538 | 1 | 19516538 | 1,6 | Os07-21309348 | 7 | 21309348 | 7,5 |
| Os01-20463894 | 1 | 20463894 | 1,6 | RM10 | 7 | 22112377 | 7,6 |
| Os01-21379853 | 1 | 21379853 | 1,6 | Os07-22344509 | 7 | 22344509 | 7,6 |
| Os01-22376228 | 1 | 22376228 | 1,6 | Os07-23234709 | 7 | 23234709 | 7,6 |
| RM9 | 1 | 23102789 | 1,7 | Os07-24210590 | 7 | 24210590 | 7,6 |
| Os01-23348132 | 1 | 23348132 | 1,7 | RM505 | 7 | 24451326 | 7,6 |
| Os01-24292397 | 1 | 24292397 | 1,7 | Os07-25225322 | 7 | 25225322 | 7,7 |
| RM488 | 1 | 24584934 | 1,7 | RM18 | 7 | 25577162 | 7,7 |
| Os01-25290506 | 1 | 25290506 | 1,7 | RM478 | 7 | 25874089 | 7,7 |
| Os01-26430565 | 1 | 26430565 | 1,8 | Os07-26327111 | 7 | 26327111 | 7,7 |
| RM246 | 1 | 27114780 | 1,8 | Os07-27495091 | 7 | 27495091 | 7,7 |
| Os01-27365821 | 1 | 27365821 | 1,8 | Os07-28445737 | 7 | 28445737 | 7,7 |
| RM473A | 1 | 27784230 | 1,8 | RM248 | 7 | 29264643 | 7,8 |
| Os01-28599049 | 1 | 28599049 | 1,8 | Os07-29303506 | 7 | 29303506 | 7,8 |
| Os01-29525464 | 1 | 29525464 | 1,9 | RM408 | 8 | 120063 | 8,1 |
| Os01-30474030 | 1 | 30474030 | 1,9 | Os08-1648452 | 8 | 1648452 | 8,1 |
| RM128 | 1 | 30517246 | 1,9 | RM38 | 8 | 2107037 | 8,1 |
| Os01-31617236 | 1 | 31617236 | 1,10 | Os08-2332138 | 8 | 2332138 | 8,1 |
| RM212 | 1 | 32833134 | 1,10 | RM25 | 8 | 4368840 | 8,2 |
| Os01-33629869 | 1 | 33629869 | 1,10 | Os08-4488034 | 8 | 4488034 | 8,2 |
| OSR3 | 1 | 34004096 | 1,10 | Os08-7745351 | 8 | 7745351 | 8,2 |
| Os01-34637122 | 1 | 34637122 | 1,10 | Os08-9543251 | 8 | 9543251 | 8,2 |
| Os01-35547034 | 1 | 35547034 | 1,10 | Os08-11559753 | 8 | 11559753 | 8,3 |
| Os01-36346421 | 1 | 36346421 | 1,11 | RM483 | 8 | 11860527 | 8,3 |
| Os01-38195426 | 1 | 38195426 | 1,11 | Os08-12381205 | 8 | 12381205 | 8,3 |
| Os01-38493222 | 1 | 38493222 | 1,11 | Os08-13485883 | 8 | 13485883 | 8,3 |
| Os01-39565366 | 1 | 39565366 | 1,12 | Os08-14485928 | 8 | 14485928 | 8,3 |
| RM104 | 1 | 39834779 | 1,12 | RM72 | 8 | 14655326 | 8,3 |
| Os01-41715970 | 1 | 41715970 | 1,12 | Os08-15431068 | 8 | 15431068 | 8,3 |
| Os01-42685494 | 1 | 42685494 | 1,12 | Os08-16538874 | 8 | 16538874 | 8,3 |
| Os01-43242647 | 1 | 43242647 | 1,12 | Os08-17449468 | 8 | 17449468 | 8,4 |
| Os02-546754 | 2 | 546754 | 2,1 | RM339 | 8 | 17789895 | 8,4 |
| OSR17 | 2 | 797624 | 2,1 | Os08-18396373 | 8 | 18396373 | 8,4 |
| RM154 | 2 | 1055553 | 2,1 | Os08-19556500 | 8 | 19556500 | 8,4 |
| Os02-1619986 | 2 | 1619986 | 2,1 | RM223 | 8 | 20497732 | 8,4 |
| RM211 | 2 | 1902309 | 2,2 | Os08-20662759 | 8 | 20662759 | 8,4 |
| Os02-2498313 | 2 | 2498313 | 2,2 | Os08-21537427 | 8 | 21537427 | 8,5 |
| RM279 | 2 | 2763989 | 2,2 | RM210 | 8 | 22323242 | 8,5 |
| Os02-3564921 | 2 | 3564921 | 2,2 | RM556 | 8 | 22323242 | 8,5 |
| RM423 | 2 | 3716161 | 2,2 | Os08-22527854 | 8 | 22527854 | 8,5 |
| Os02-5670969 | 2 | 5670969 | 2,2 | Os08-23573379 | 8 | 23573379 | 8,5 |
| Os02-6688558 | 2 | 6688558 | 2,3 | RM149 | 8 | 24551285 | 8,5 |
| Os02-7621053 | 2 | 7621053 | 2,3 | Os08-24714824 | 8 | 24714824 | 8,5 |
| Os02-8454851 | 2 | 8454851 | 2,3 | RM447 | 8 | 26378759 | 8,6 |
| RM452 | 2 | 9325914 | 2,4 | Os08-26929959 | 8 | 26929959 | 8,6 |
| Os02-9511612 | 2 | 9511612 | 2,4 | RM264 | 8 | 27762624 | 8,6 |
| RM29 | 2 | 10073225 | 2,4 | Os08-27849015 | 8 | 27849015 | 8,6 |
| Os02-10693848 | 2 | 10693848 | 2,4 | Os08-28121217 | 8 | 28121217 | 8,6 |
| RM324 | 2 | 11152005 | 2,4 | Os09-495537 | 9 | 495537 | 9,1 |
| Os02-11540193 | 2 | 11540193 | 2,4 | RM316 | 9 | 594997 | 9,1 |
| Os02-12601312 | 2 | 12601312 | 2,4 | Os09-1703763 | 9 | 1703763 | 9,1 |
| Os02-13541115 | 2 | 13541115 | 2,4 | Os09-2663954 | 9 | 2663954 | 9,1 |
| Os02-14549229 | 2 | 14549229 | 2,4 | Os09-3741520 | 9 | 3741520 | 9,1 |
| Os02-15523977 | 2 | 15523977 | 2,5 | Os09-4454591 | 9 | 4454591 | 9,1 |
| Os02-16672965 | 2 | 16672965 | 2,5 | Os09-5390612 | 9 | 5390612 | 9,1 |
| Os02-17799722 | 2 | 17799722 | 2,5 | Os09-6247346 | 9 | 6247346 | 9,1 |
| Os02-18559893 | 2 | 18559893 | 2,5 | RM219 | 9 | 6732513 | 9,1 |
| RM341 | 2 | 19003256 | 2,5 | Os09-7237057 | 9 | 7237057 | 9,2 |
| Os02-19461406 | 2 | 19461406 | 2,5 | Os09-8118408 | 9 | 8118408 | 9,2 |
| RM475 | 2 | 20065980 | 2,6 | Os09-9263330 | 9 | 9263330 | 9,2 |
| Os02-20579937 | 2 | 20579937 | 2,6 | Os09-10252513 | 9 | 10252513 | 9,3 |
| Os02-21466875 | 2 | 21466875 | 2,6 | RM409 | 9 | 12969484 | 9,3 |
| Os02-22431270 | 2 | 22431270 | 2,6 | Os09-13116310 | 9 | 13116310 | 9,4 |
| Os02-23557743 | 2 | 23557743 | 2,6 | Os09-14143356 | 9 | 14143356 | 9,4 |
| Os02-24626496 | 2 | 24626496 | 2,6 | Os09-15126094 | 9 | 15126094 | 9,5 |
| RM263 | 2 | 25437455 | 2,6 | Os09-15999535 | 9 | 15999535 | 9,5 |
| Os02-26438150 | 2 | 26438150 | 2,7 | RM257 | 9 | 16190038 | 9,6 |
| RM221 | 2 | 27169065 | 2,7 | Os09-17141056 | 9 | 17141056 | 9,6 |
| Os02-27547601 | 2 | 27547601 | 2,7 | RM242 | 9 | 17260816 | 9,7 |
| Os02-28570388 | 2 | 28570388 | 2,7 | RM278 | 9 | 17684185 | 9,7 |
| RM318 | 2 | 29197086 | 2,7 | RM160 | 9 | 17900563 | 9,7 |
| Os02-29550138 | 2 | 29550138 | 2,8 | OSR28 | 9 | 17900669 | 9,7 |
| Os02-30514689 | 2 | 30514689 | 2,8 | RM328 | 9 | 17999076 | 9,7 |
| RM240 | 2 | 31062885 | 2,8 | RM201 | 9 | 18408763 | 9,7 |
| Os02-31573993 | 2 | 31573993 | 2,9 | RM189 | 9 | 18630574 | 9,7 |
| RM112 | 2 | 31579259 | 2,9 | Os09-19102345 | 9 | 19102345 | 9,7 |
| Os02-32483490 | 2 | 32483490 | 2,9 | RM215 | 9 | 19336339 | 9,7 |
| Os02-33407254 | 2 | 33407254 | 2,9 | Os09-20111040 | 9 | 20111040 | 9,8 |
| Os02-34434515 | 2 | 34434515 | 2,9 | RM205 | 9 | 20809538 | 9,8 |
| RM208 | 2 | 34647140 | 2,9 | Os09-21013912 | 9 | 21013912 | 9,8 |
| RM48 | 2 | 34937603 | 2,9 | Os09-22008191 | 9 | 22008191 | 9,8 |
| Os02-35377220 | 2 | 35377220 | 2,9 | Os10-426268 | 10 | 426268 | 10,1 |
| Os03-523788 | 3 | 523788 | 3,1 | Os10-1539800 | 10 | 1539800 | 10,1 |
| Os03-1608489 | 3 | 1608489 | 3,2 | Os10-2517878 | 10 | 2517878 | 10,1 |
| RM81B | 3 | 1927290 | 3,2 | Os10-3529118 | 10 | 3529118 | 10,1 |
| RM231 | 3 | 2436313 | 3,3 | Os10-4605896 | 10 | 4605896 | 10,2 |
| Os03-2592303 | 3 | 2592303 | 3,3 | RM216 | 10 | 4992759 | 10,2 |
| Os03-3502670 | 3 | 3502670 | 3,4 | Os10-5711184 | 10 | 5711184 | 10,2 |
| RM175 | 3 | 3848572 | 3,4 | Os10-6569546 | 10 | 6569546 | 10,2 |
| RM489 | 3 | 4318347 | 3,5 | Os10-7518016 | 10 | 7518016 | 10,2 |
| Os03-4681740 | 3 | 4681740 | 3,5 | Os10-8485346 | 10 | 8485346 | 10,2 |
| Os03-5594346 | 3 | 5594346 | 3,5 | RM311 | 10 | 9352577 | 10,3 |
| RM218 | 3 | 8384478 | 3,6 | Os10-9511974 | 10 | 9511974 | 10,3 |
| Os03-8511901 | 3 | 8511901 | 3,6 | Os10-10592168 | 10 | 10592168 | 10,3 |
| Os03-9537796 | 3 | 9537796 | 3,6 | Os10-11585840 | 10 | 11585840 | 10,3 |
| RM251 | 3 | 9929306 | 3,6 | Os10-12700842 | 10 | 12700842 | 10,3 |
| Os03-10696698 | 3 | 10696698 | 3,6 | RM467 | 10 | 13057268 | 10,3 |
| Os03-11603210 | 3 | 11603210 | 3,7 | Os10-13707801 | 10 | 13707801 | 10,3 |
| RM282 | 3 | 12388424 | 3,7 | RM271 | 10 | 16207691 | 10,4 |
| Os03-12590793 | 3 | 12590793 | 3,7 | Os10-16717537 | 10 | 16717537 | 10,4 |
| Os03-14501282 | 3 | 14501282 | 3,7 | RM258 | 10 | 17585202 | 10,4 |
| Os03-15411439 | 3 | 15411439 | 3,7 | Os10-17678111 | 10 | 17678111 | 10,4 |
| Os03-16699322 | 3 | 16699322 | 3,8 | RM304 | 10 | 18226089 | 10,5 |
| RM156 | 3 | 17493344 | 3,8 | Os10-18588616 | 10 | 18588616 | 10,5 |
| Os03-17511092 | 3 | 17511092 | 3,8 | RM147 | 10 | 20516504 | 10,6 |
| Os03-18569787 | 3 | 18569787 | 3,8 | Os10-21083021 | 10 | 21083021 | 10,6 |
| Os03-19584698 | 3 | 19584698 | 3,8 | RM228 | 10 | 21811827 | 10,6 |
| Os03-20562296 | 3 | 20562296 | 3,8 | Os10-22013168 | 10 | 22013168 | 10,6 |
| Os03-21638313 | 3 | 21638313 | 3,8 | RM590 | 10 | 22611742 | 10,6 |
| Os03-22721653 | 3 | 22721653 | 3,8 | Os10-23008415 | 10 | 23008415 | 10,6 |
| RM16 | 3 | 22784730 | 3,8 | RM286 | 11 | 382385 | 11,1 |
| Os03-23524842 | 3 | 23524842 | 3,9 | Os11-749467 | 11 | 749467 | 11,1 |
| Os03-24694950 | 3 | 24694950 | 3,9 | RM20B | 11 | 967612 | 11,1 |
| Os03-25422606 | 3 | 25422606 | 3,9 | Os11-1767997 | 11 | 1767997 | 11,1 |
| Os03-26517642 | 3 | 26517642 | 3,9 | RM332 | 11 | 2837412 | 11,2 |
| OSR31 | 3 | 27141989 | 3,9 | Os11-2857452 | 11 | 2857452 | 11,2 |
| Os03-27447623 | 3 | 27447623 | 3,9 | Os11-3624911 | 11 | 3624911 | 11,2 |
| RM168 | 3 | 27821947 | 3,9 | RM167 | 11 | 4057316 | 11,2 |
| Os03-28485578 | 3 | 28485578 | 3,9 | Os11-4572851 | 11 | 4572851 | 11,2 |
| Os03-29485150 | 3 | 29485150 | 3,10 | Os11-5558765 | 11 | 5558765 | 11,2 |
| Os03-30731516 | 3 | 30731516 | 3,10 | RM120 | 11 | 5676719 | 11,2 |
| RM293 | 3 | 31369642 | 3,10 | Os11-6494479 | 11 | 6494479 | 11,2 |
| Os03-32586703 | 3 | 32586703 | 3,11 | Os11-7401196 | 11 | 7401196 | 11,2 |
| Os03-33488900 | 3 | 33488900 | 3,11 | Os11-8413857 | 11 | 8413857 | 11,2 |
| Os03-34480985 | 3 | 34480985 | 3,11 | RM202 | 11 | 8788424 | 11,3 |
| RM227 | 3 | 34644785 | 3,12 | Os11-9429954 | 11 | 9429954 | 11,3 |
| RM570 | 3 | 35307882 | 3,12 | Os11-10425720 | 11 | 10425720 | 11,3 |
| Os03-35576704 | 3 | 35576704 | 3,12 | Os11-11356699 | 11 | 11356699 | 11,3 |
| RM148 | 3 | 35673541 | 3,12 | Os11-12596218 | 11 | 12596218 | 11,3 |
| RM85 | 3 | 36060505 | 3,12 | Os11-13616357 | 11 | 13616357 | 11,3 |
| Os03-36341768 | 3 | 36341768 | 3,12 | Os11-14498642 | 11 | 14498642 | 11,3 |
| RM307 | 4 | 0 | 4,1 | Os11-15445681 | 11 | 15445681 | 11,3 |
| RM401 | 4 | 50000 | 4,1 | RM209 | 11 | 17307615 | 11,3 |
| RM551 | 4 | 168620 | 4,1 | Os11-17512870 | 11 | 17512870 | 11,3 |
| Os04-559977 | 4 | 559977 | 4,1 | RM229 | 11 | 17813138 | 11,3 |
| RM335 | 4 | 679761 | 4,1 | Os11-18417540 | 11 | 18417540 | 11,3 |
| Os04-1356902 | 4 | 1356902 | 4,1 | RM21 | 11 | 18594224 | 11,4 |
| Os04-2249986 | 4 | 2249986 | 4,2 | RM260 | 11 | 19329654 | 11,4 |
| Os04-3644948 | 4 | 3644948 | 4,2 | Os11-19520671 | 11 | 19520671 | 11,4 |
| Os04-4560663 | 4 | 4560663 | 4,2 | Os11-20518958 | 11 | 20518958 | 11,5 |
| Os04-5587643 | 4 | 5587643 | 4,2 | Os11-21455000 | 11 | 21455000 | 11,5 |
| RM261 | 4 | 6396838 | 4,3 | RM254 | 11 | 22957018 | 11,6 |
| Os04-6556560 | 4 | 6556560 | 4,3 | Os11-23326755 | 11 | 23326755 | 11,6 |
| Os04-7543099 | 4 | 7543099 | 4,3 | Os11-24347072 | 11 | 24347072 | 11,7 |
| Os04-8510676 | 4 | 8510676 | 4,3 | RM330A | 11 | 24709563 | 11,7 |
| Os04-10727312 | 4 | 10727312 | 4,3 | Os11-25388065 | 11 | 25388065 | 11,7 |
| Os04-11560423 | 4 | 11560423 | 4,3 | RM224 | 11 | 26394760 | 11,7 |
| Os04-12588963 | 4 | 12588963 | 4,3 | Os11-26503578 | 11 | 26503578 | 11,7 |
| Os04-13503095 | 4 | 13503095 | 4,4 | Os11-27568736 | 11 | 27568736 | 11,7 |
| Os04-14172065 | 4 | 14172065 | 4,4 | Os11-28398373 | 11 | 28398373 | 11,7 |
| Os04-14516570 | 4 | 14516570 | 4,4 | Os12-263546 | 12 | 263546 | 12,1 |
| Os04-15817368 | 4 | 15817368 | 4,4 | RM20A | 12 | 737414 | 12,1 |
| Os04-16391705 | 4 | 16391705 | 4,4 | RM4A | 12 | 931998 | 12,1 |
| Os04-17382793 | 4 | 17382793 | 4,4 | Os12-1346402 | 12 | 1346402 | 12,2 |
| Os04-18321788 | 4 | 18321788 | 4,4 | Os12-2374763 | 12 | 2374763 | 12,2 |
| RM417 | 4 | 19123096 | 4,4 | RM19 | 12 | 2400573 | 12,2 |
| Os04-19483066 | 4 | 19483066 | 4,4 | RM247 | 12 | 3150134 | 12,2 |
| Os04-20543400 | 4 | 20543400 | 4,5 | Os12-3356582 | 12 | 3356582 | 12,2 |
| RM119 | 4 | 20942103 | 4,5 | Os12-4033132 | 12 | 4033132 | 12,2 |
| Os04-21139448 | 4 | 21139448 | 4,5 | Os12-5044751 | 12 | 5044751 | 12,2 |
| Os04-22701665 | 4 | 22701665 | 4,5 | RM512 | 12 | 5068591 | 12,2 |
| Os04-23551250 | 4 | 23551250 | 4,5 | Os12-5774979 | 12 | 5774979 | 12,3 |
| Os04-24600881 | 4 | 24600881 | 4,5 | Os12-6908827 | 12 | 6908827 | 12,3 |
| RM252 | 4 | 24861268 | 4,5 | Os12-7802056 | 12 | 7802056 | 12,3 |
| Os04-25526863 | 4 | 25526863 | 4,5 | RM101 | 12 | 8794705 | 12,4 |
| Os04-26638271 | 4 | 26638271 | 4,6 | Os12-8818220 | 12 | 8818220 | 12,4 |
| Os04-27673866 | 4 | 27673866 | 4,6 | Os12-9930013 | 12 | 9930013 | 12,4 |
| RM470 | 4 | 27773031 | 4,6 | Os12-10528353 | 12 | 10528353 | 12,4 |
| RM303 | 4 | 28257505 | 4,6 | Os12-12268471 | 12 | 12268471 | 12,4 |
| Os04-28545803 | 4 | 28545803 | 4,6 | Os12-13264289 | 12 | 13264289 | 12,4 |
| Os04-29499584 | 4 | 29499584 | 4,6 | Os12-14231084 | 12 | 14231084 | 12,4 |
| Os04-30541070 | 4 | 30541070 | 4,7 | Os12-15786253 | 12 | 15786253 | 12,4 |
| RM255 | 4 | 31006857 | 4,7 | Os12-16301470 | 12 | 16301470 | 12,4 |
| Os04-31485158 | 4 | 31485158 | 4,7 | Os12-17348984 | 12 | 17348984 | 12,4 |
| Os04-32513565 | 4 | 32513565 | 4,7 | RM277 | 12 | 18100739 | 12,4 |
| RM348 | 4 | 32824953 | 4,7 | Os12-18360349 | 12 | 18360349 | 12,4 |
| Os04-33447587 | 4 | 33447587 | 4,8 | Os12-19486444 | 12 | 19486444 | 12,5 |
| Os04-34479297 | 4 | 34479297 | 4,8 | Os12-20077695 | 12 | 20077695 | 12,5 |
| RM280 | 4 | 34672357 | 4,8 | RM463 | 12 | 21206492 | 12,5 |
| Os04-35246701 | 4 | 35246701 | 4,8 | Os12-21208144 | 12 | 21208144 | 12,5 |
| RM159 | 5 | 458568 | 5,1 | Os12-22220155 | 12 | 22220155 | 12,5 |
| Os05-497086 | 5 | 497086 | 5,1 | Os12-24328785 | 12 | 24328785 | 12,5 |
| Os05-1502294 | 5 | 1502294 | 5,1 | Os12-25495817 | 12 | 25495817 | 12,6 |
| Os05-2499026 | 5 | 2499026 | 5,2 | RM235 | 12 | 25718612 | 12,6 |
| RM13 | 5 | 2713543 | 5,2 | Os12-26519997 | 12 | 26519997 | 12,6 |
| Os05-3609253 | 5 | 3609253 | 5,2 | RM17 | 12 | 26567985 | 12,6 |
| Os05-4578273 | 5 | 4578273 | 5,2 | Os12-27417700 | 12 | 27417700 | 12,6 |
| Os05-5389716 | 5 | 5389716 | 5,3 |  |  |  |  |
| Os05-6448201 | 5 | 6448201 | 5,3 |  |  |  |  |
| RM289 | 5 | 7304912 | 5,3 |  |  |  |  |
| Os05-8636547 | 5 | 8636547 | 5,3 |  |  |  |  |
| Os05-10338341 | 5 | 10338341 | 5,4 |  |  |  |  |
| RM249 | 5 | 10755867 | 5,4 |  |  |  |  |
| Os05-11060652 | 5 | 11060652 | 5,4 |  |  |  |  |
| Os05-12440254 | 5 | 12440254 | 5,4 |  |  |  |  |
| Os05-13396654 | 5 | 13396654 | 5,4 |  |  |  |  |
| Os05-16372023 | 5 | 16372023 | 5,5 |  |  |  |  |
| Os05-17301701 | 5 | 17301701 | 5,5 |  |  |  |  |
| RM430 | 5 | 17889982 | 5,5 |  |  |  |  |
| Os05-18339192 | 5 | 18339192 | 5,5 |  |  |  |  |
| RM163 | 5 | 18347236 | 5,5 |  |  |  |  |
| Os05-19546328 | 5 | 19546328 | 5,6 |  |  |  |  |
| RM161 | 5 | 19995999 | 5,6 |  |  |  |  |
| Os05-20376558 | 5 | 20376558 | 5,6 |  |  |  |  |
| Os05-22370060 | 5 | 22370060 | 5,6 |  |  |  |  |
| RM421 | 5 | 23115889 | 5,7 |  |  |  |  |
| Os05-23381810 | 5 | 23381810 | 5,7 |  |  |  |  |
| RM87 | 5 | 26082064 | 5,8 |  |  |  |  |
| Os05-29490255 | 5 | 29490255 | 5,9 |  |  |  |  |
| Os06-630955 | 6 | 630955 | 6,1 |  |  |  |  |
| RM190 | 6 | 1741174 | 6,2 |  |  |  |  |
| Os06-2461121 | 6 | 2461121 | 6,3 |  |  |  |  |
| RM204 | 6 | 3124852 | 6,3 |  |  |  |  |
| RM225 | 6 | 3373065 | 6,3 |  |  |  |  |
| Os06-3460330 | 6 | 3460330 | 6,3 |  |  |  |  |
| Os06-4403271 | 6 | 4403271 | 6,3 |  |  |  |  |
| RM253 | 6 | 5468147 | 6,3 |  |  |  |  |
| RM276 | 6 | 6284678 | 6,3 |  |  |  |  |
| Os06-6369670 | 6 | 6369670 | 6,3 |  |  |  |  |
| RM50 | 6 | 6431526 | 6,3 |  |  |  |  |
| Os06-8126604 | 6 | 8126604 | 6,4 |  |  |  |  |
| RM136 | 6 | 8812715 | 6,4 |  |  |  |  |
| Os06-9146196 | 6 | 9146196 | 6,4 |  |  |  |  |
| Os06-9656257 | 6 | 9656257 | 6,4 |  |  |  |  |
| Os06-10551258 | 6 | 10551258 | 6,4 |  |  |  |  |
| Os06-11476061 | 6 | 11476061 | 6,4 |  |  |  |  |
| Os06-12476075 | 6 | 12476075 | 6,4 |  |  |  |  |
| Os06-13540706 | 6 | 13540706 | 6,4 |  |  |  |  |
| Os06-14536739 | 6 | 14536739 | 6,4 |  |  |  |  |
| Os06-16517699 | 6 | 16517699 | 6,4 |  |  |  |  |
| Os06-18613336 | 6 | 18613336 | 6,5 |  |  |  |  |
| RM3 | 6 | 18619035 | 6,5 |  |  |  |  |
| Os06-20500433 | 6 | 20500433 | 6,5 |  |  |  |  |
| Os06-21660050 | 6 | 21660050 | 6,5 |  |  |  |  |
| Os06-22374725 | 6 | 22374725 | 6,5 |  |  |  |  |
| Os06-23225896 | 6 | 23225896 | 6,5 |  |  |  |  |
| RM275 | 6 | 23423365 | 6,5 |  |  |  |  |
| Os06-24249085 | 6 | 24249085 | 6,6 |  |  |  |  |
| Os06-25238549 | 6 | 25238549 | 6,6 |  |  |  |  |
| Os06-26104673 | 6 | 26104673 | 6,6 |  |  |  |  |
| RM30 | 6 | 26308239 | 6,6 |  |  |  |  |
| Os06-27310377 | 6 | 27310377 | 6,7 |  |  |  |  |
| RM340 | 6 | 27655224 | 6,7 |  |  |  |  |
| Os06-28333687 | 6 | 28333687 | 6,7 |  |  |  |  |
| RM439 | 6 | 28556762 | 6,7 |  |  |  |  |
| Os06-29381647 | 6 | 29381647 | 6,7 |  |  |  |  |
| RM141 | 6 | 29801098 | 6,7 |  |  |  |  |
| Os06-30008547 | 6 | 30008547 | 6,7 |  |  |  |  |
| Os06-31084101 | 6 | 31084101 | 6,7 |  |  |  |  |
