# Supplementary material for: SS1 (NAL1)- and SS2-Mediated Genetic Networks Underlying Source-Sink and Yield Traits in Rice (Oryza sativa L.)
Source: PLoS One. 2015 Jul 10;10(7):e0132060. doi: 10.1371/journal.pone.0132060 (PMC4498882; doi:10.1371/journal.pone.0132060)
Supplement: S2 Table — (DOC) [file pone.0132060.s010.doc]

**S2 Table** InDel and CAPS markers designed for fine mapping of the *SS1*

| Marker | Marker type | BAC location | Forward primer sequence (5'-3') | Reverse primer sequence (5'-3') | Predicted  Size (bp)^a^ | Restriction  enzyme |
| --- | --- | --- | --- | --- | --- | --- |
| FL25 | CAPs | AL731610 | ACAGTACCTTCATTCAGCCTCTTC | GATGCGTTCGTCTCACCAGTT | 367 | *Fok*I |
| FL40 | CAPs | AL662950 | TTTTGGATGTATTCGCTGTT | AATGAAACCCTGGCAATA | 176 | *Hph*I |
| JY3 | Indel | AL606648.3 | GCAGTGCCCAACTTGTTT | ACGCTAGTAGGCAGAGGC | 269 |  |
| WY17 | Indel | AL662970 | TTCGTAGTGGGCTTGGAG | GGAGGAAGGAAGTGAGGC | 200 |  |
| FL41 | CAPs | AL662950 | AAACCCTGGCATTACATT | CATAGACATAAGAACCCT | 171 | *Dde*I |
| FL95 | CAPs | AL662950 | GAGCAAGTTTCAGTGAGGGAG | AAATGGAGCACAGCAGAC | 274 | *Alu*I |
| FL98 | CAPs | AL662950 | GCCTTGACCTGGAGAAAT | CAGGACTAGCACCTAACATC | 253 | *Hph*I |
| WY21 | Indel | AL662950 | ACCAAGAAATCAGCGACCAC | TCCATCAGCAGCGAACAC | 224 |  |

^a^ PCR product size was estimated based on Nipponbare genome sequence.
