# Supplementary material for: SS1 (NAL1)- and SS2-Mediated Genetic Networks Underlying Source-Sink and Yield Traits in Rice (Oryza sativa L.)
Source: PLoS One. 2015 Jul 10;10(7):e0132060. doi: 10.1371/journal.pone.0132060 (PMC4498882; doi:10.1371/journal.pone.0132060)
Supplement: S3 Table — (DOC) [file pone.0132060.s011.doc]

**S3 Table** Primers used for sequencing analysis and quantitative real-time PCR (qRT-PCR) of *SS1*

| Primer  application | Targeted gene | Forward primer sequence (5'-3') | Reverse primer sequence (5'-3') | Predicted  Size (bp) ^a^ |
| --- | --- | --- | --- | --- |
| qRT-PCR | *LOC_Os04g52440* | CAACATCAAGCCAGATCTCG | AGTATGTAAAGCCATGAGCAA | 149 |
|  | *LOC_Os04g52450* | TAACATCAAGCCAGATCTAGTCTC | TCCATGAGCAAATGAACCGA | 139 |
|  | *LOC_Os04g52479* | GCCTTATTATCCTGACTAGCCAA | ATCAACCCCACTAGTCCA | 134 |
| Sequencing  analysis | Full-length cDNA of *LOC_Os04g52440* | ATGAATTTGATAAAGCATGCAGCTTTTGCTG | CTATTTTTTCTTGGACCTCAGCTCTGCC | 1494 |
|  | Promoter region of  *LOC_Os04g52440* | GGATTCTGGAATAGATATATATTTGTATTTCTAATTCG | CCTCTGATCTTTCAATAATCAAGGGC | 1443(LT/NIL), 1458(TQ) |
|  | Full-length cDNA of *LOC_Os04g52450* | ATGGTGATTGCGCGCGGC | CTAATTGTTCTTCTTGGATTTCAGCTCAGCCAC | 1551 |
|  | Promoter region of  *LOC_Os04g52450* | ACTGAATTTGCTAATAACAAATCACCAAATGATCC | CCCTTTAGAATCTGCACAGTCCGTG | 1876(LT/NIL), 1914(TQ) |
|  | Full-length cDNA of *LOC_Os04g52479* | ATGAAGCCTTCGGACGATAAGGCG | TCATTTCTCCAGGTCAAGGCTTGATCC | 1749 |
|  | Promoter region of  *LOC_Os04g52479* | AGTGTGAACTGCTCTCTAGCCTAGATC | CTGCACAATGCTGTAGGTTGGATGTC | 1502 |

^a^ PCR product size was estimated based on Nipponbare genome sequence.
