# Supplementary material for: SS1 (NAL1)- and SS2-Mediated Genetic Networks Underlying Source-Sink and Yield Traits in Rice (Oryza sativa L.)
Source: PLoS One. 2015 Jul 10;10(7):e0132060. doi: 10.1371/journal.pone.0132060 (PMC4498882; doi:10.1371/journal.pone.0132060)
Supplement: S4 Table — (DOC) [file pone.0132060.s012.doc]

**S4 Table** Summarized statistics of the reciprocal introgression lines (ILs) and their parents, Lemont (LT) and Teqing (TQ) for five source leaf and yield traits evaluated in Beijing (BJ) and Sanya (SY)

| Env. | Trait^a^ | Parents | | | TQ-ILs | | LT-ILs | |
| --- | --- | --- | --- | --- | --- | --- | --- | --- |
|  |  | TQ | LT | P_1_ – P_2_^b^ | Mean ± SD | Range | Mean ± SD | Range |
| SY | FLL (cm) | 31.3 | 23.4 | 7.9** | 26.0±3.6 | 18.1 – 47.0 | 23.6± 3.3 | 15.1 – 32.8 |
|  | FLW (mm) | 16.7 | 22.0 | -5.3** | 16.7±1.5 | 12.1 – 23.9 | 20.1±1.7 | 15.2 – 25.7 |
|  | GNP | 231.7 | 167.5 | 64.2* | 217.9±36.7 | 100.2 – 384.8 | 181.7±46.7 | 77.5 – 345.5 |
|  | GW (g) | 23.0 | 22.3 | 0.7 | 23.4± 2.0 | 15.8 – 29.9 | 22.3± 1.9 | 17.8 – 28.8 |
|  | GY (g) | 47.9 | 17.2 | 30.7** | 28.2±7.4 | 9.0 – 46.5 | 16.3± 7.2 | 3.4 – 65.9 |
| BJ | FLL (cm) | 33.0 | 24.5 | 8.5** | 29.8±4.3 | 19.5 – 45.6 | 30.4±4.2 | 17.1 – 42.5 |
|  | FLW (mm) | 17.5 | 21.8 | -4.3** | 17.5±1.4 | 14.1 – 24.3 | 21.1±1.6 | 16.4 – 25.5 |
|  | GNP | 217.4 | 153.6 | 63.8* | 198.7±43.3 | 92.8 – 357.5 | 145.5±31.5 | 71.1 – 298.6 |
|  | GW (g) | 24.9 | 24.4 | 0.5 | 22.1±2.0 | 14.9 – 29.1 | 22.2±2.2 | 12.8 – 29.0 |
|  | GY (g) | 53.4 | 18.6 | 34.8** | 29.7±6.9 | 4.6 – 45.6 | 22.6±7.7 | 6.0 – 61.8 |

^a^ FLL: flag leaf length (cm) , FLW: flag leaf width (mm), GNP: grain number per panicle, GW: 1000- grain weight (g), GY: grain yield per plant (g).

^b^ *and ** indicate the significant difference levels at P ≤ 0.05 and 0.01 based on *t* tests, respectively.
