# Supplementary material for: SS1 (NAL1)- and SS2-Mediated Genetic Networks Underlying Source-Sink and Yield Traits in Rice (Oryza sativa L.)
Source: PLoS One. 2015 Jul 10;10(7):e0132060. doi: 10.1371/journal.pone.0132060 (PMC4498882; doi:10.1371/journal.pone.0132060)
Supplement: S5 Table — (DOC) [file pone.0132060.s013.doc]

**S5 Table** ANOVA results of the reciprocal introgression lines (ILs) of Teqing (TQ) and Lemont (LT) for five source leaf and yield traits evaluated in Beijing (BJ) and Sanya (SY)

| Population | Trait^a^ | Source of Variation | df | SS | MS | F | P value | R^2^ (%) |
| --- | --- | --- | --- | --- | --- | --- | --- | --- |
| TQ-ILs | FLW | Genotype (G) | 253 | 16.4 | 0.1 | 7.7 | <.0001 | 58.8 |
|  |  | Environment (E) | 1 | 1.3 | 1.3 | 158.4 | <.0001 | 4.7 |
|  |  | G x E | 253 | 3.6 | 0.0 | 1.7 | <.0001 | 12.9 |
|  | FLL | Genotype (G) | 253 | 10660.8 | 42.5 | 3.8 | <.0001 | 42.9 |
|  |  | Environment (E) | 1 | 3403.0 | 3403.0 | 303.6 | <.0001 | 13.7 |
|  |  | G x E | 253 | 5115.7 | 20.7 | 1.9 | <.0001 | 20.6 |
|  | GNP | Genotype (G) | 253 | 902995.8 | 3597.6 | 3.4 | <.0001 | 47.8 |
|  |  | Environment (E) | 1 | 141618.3 | 141618.3 | 134.3 | <.0001 | 7.5 |
|  |  | G x E | 253 | 307147.4 | 1243.5 | 1.2 | 0.0654 | 16.3 |
|  | GW | Genotype (G) | 253 | 3194.9 | 12.7 | 14.0 | <.0001 | 68.0 |
|  |  | Environment (E) | 1 | 399.6 | 399.6 | 439.0 | <.0001 | 8.5 |
|  |  | G x E | 253 | 643.8 | 2.6 | 2.9 | <.0001 | 13.7 |
|  | GY | Genotype (G) | 253 | 54989.0 | 219.1 | 2.0 | <.0001 | 21.0 |
|  |  | Environment (E) | 1 | 98652.5 | 98652.5 | 889.3 | <.0001 | 37.6 |
|  |  | G x E | 253 | 52983.6 | 219.8 | 2.0 | <.0001 | 20.2 |
| LT-ILs | FLW | Genotype (G) | 253 | 17.8 | 0.1 | 9.8 | <.0001 | 62.9 |
|  |  | Environment (E) | 1 | 2.0 | 2.0 | 217.0 | <.0001 | 7.1 |
|  |  | G x E | 253 | 3.3 | 0.0 | 1.8 | <.0001 | 11.7 |
|  | FLL | Genotype (G) | 253 | 8428.5 | 42.1 | 5.5 | <.0001 | 34.3 |
|  |  | Environment (E) | 1 | 9240.6 | 9240.6 | 1198.4 | <.0001 | 37.6 |
|  |  | G x E | 253 | 3024.6 | 15.2 | 2.0 | <.0001 | 12.3 |
|  | GNP | Genotype (G) | 253 | 703318.5 | 3516.6 | 6.0 | <.0001 | 49.7 |
|  |  | Environment (E) | 1 | 205715.5 | 205715.5 | 351.6 | <.0001 | 14.5 |
|  |  | G x E | 253 | 209730.4 | 1048.7 | 1.8 | <.0001 | 14.8 |
|  | GW | Genotype (G) | 253 | 2475.1 | 12.4 | 9.9 | <.0001 | 67.5 |
|  |  | Environment (E) | 1 | 2.1 | 2.1 | 1.7 | 0.1942 | 0.1 |
|  |  | G x E | 253 | 555.0 | 2.8 | 2.2 | <.0001 | 15.1 |
|  | GY | Genotype (G) | 253 | 64081.3 | 320.4 | 5.2 | <.0001 | 34.2 |
|  |  | Environment (E) | 1 | 63662.2 | 63662.2 | 1035.6 | <.0001 | 34.0 |
|  |  | G x E | 253 | 28465.8 | 144.5 | 2.4 | <.0001 | 15.2 |

^a^ FLW: flag leaf width (mm), FLL: flag leaf length (cm), GNP: grain number per panicle, GW: 1000-grain weight (g), GY: grain yield per plant (g).
