# Supplementary material for: SS1 (NAL1)- and SS2-Mediated Genetic Networks Underlying Source-Sink and Yield Traits in Rice (Oryza sativa L.)
Source: PLoS One. 2015 Jul 10;10(7):e0132060. doi: 10.1371/journal.pone.0132060 (PMC4498882; doi:10.1371/journal.pone.0132060)
Supplement: S6 Table — (DOC) [file pone.0132060.s014.doc]

**S6 Table** Main-effect QTL affecting SS-related traits flag leaf width (FLW, in mm) and length (FLL, in cm), grain number per panicle (GNP), 1000-grain weight (GW, in g) and grain yield per plant (GY, in g) detected in the LT/TQ reciprocal IL populations and two environments (Beijing – BJ and Sanya – SY)

| Upstream QTL ^a^ | | QTL ^b^ | Bin ^c^ | Pop. - Env. | Trait | LOD | *A* ^d^ | R^2^ (%) |
| --- | --- | --- | --- | --- | --- | --- | --- | --- |
| ***qFll3.12*** | | *qFll1.8* | 1.8 | TQ-BJ | FLL | 6.96 | 1.8 | 7.8 |
| ***qGnp4.7/qGnp3.12*** | | *qGnp1.8* |  | TQ-SY | GNP | 4.69 | -13.9 | 5.3 |
|  | | *qGw1.8* |  | TQ-BJ | GW | 6.84 | -0.8 | 7.2 |
|  | |  |  | TQ-SY | GW | 4.19 | -0.7 | 5.2 |
| ***qFlw4.7*** | | *qFlw2.4* | 2.4 | TQ-BJ | FLW | 10.97 | 0.9 | 14.3 |
|  | | *qGw2.4* |  | TQ-BJ | GW | 5.18 | 0.8 | 6.3 |
|  | |  |  | TQ-SY | GW | 3.54 | 1.0 | 8.4 |
| ***qFll3.12*** | | *qFll2.6* | 2.6 | TQ-BJ | FLL | 3.08 | 1.7 | 5.6 |
| ***qFll3.12*** | | *qFll3.5* | 3.5 | TQ-SY | FLL | 2.49 | 4.2 | 14.7 |
| ***qFlw4.7*** | | *qFlw3.5* |  | TQ-BJ | FLW | 4.84 | -0.9 | 3.7 |
|  | |  |  | TQ-SY | FLW | 3.01 | -0.9 | 3.5 |
| ***SS2*** | | *qFll3.12* | 3.12 | TQ-BJ | FLL | 7.04 | -1.6 | 8.0 |
|  | |  |  | TQ-SY | FLL | 3.05 | -1.8 | 5.0 |
| ***SS2*** | | *qGnp3.12* |  | TQ-BJ | GNP | 3.04 | -19.5 | 4.3 |
|  | |  |  | TQ-SY | GNP | 6.54 | -19.8 | 11.3 |
| ***SS2*** | | *qGw3.12a* |  | TQ-BJ | GW | 5.30 | 1.2 | 7.6 |
|  | |  |  | TQ-SY | GW | 2.79 | 1.1 | 8.9 |
|  | | *qFll4.1* | 4.1 | TQ-SY | FLL | 10.13 | 2.5 | 22.3 |
| ***qGnp4.7/qGnp3.12*** | | *qGnp4.1* |  | TQ-BJ | GNP | 3.67 | -12.9 | 5.3 |
|  | |  |  | TQ-SY | GNP | 2.79 | -11.9 | 3.1 |
|  | | *qGw4.1* |  | TQ-SY | GW | 10.09 | -1.2 | 11.4 |
|  | | *qGy4.1* |  | TQ-BJ | GY | 2.22 | -2.2 | 4.9 |
| ***SS1*** | | *qFlw4.7* | 4.7 | TQ-BJ | FLW | 11.20 | 1.3 | 14.0 |
|  | |  |  | TQ-SY | FLW | 6.80 | 0.9 | 14.0 |
| ***SS1*** | | *qGnp4.7* |  | TQ-BJ | GNP | 6.86 | 19.3 | 10.7 |
|  | |  |  | TQ-SY | GNP | 3.39 | 13.7 | 4.0 |
| ***qGnp3.12*** | | *qGnp5.5* | 5.5 | TQ-BJ | GNP | 5.14 | 16.8 | 8.5 |
|  | | *qGw5.5* |  | TQ-BJ | GW | 4.39 | -0.8 | 6.4 |
|  | |  |  | TQ-SY | GW | 11.11 | -1.5 | 21.8 |
| ***qFlw4.7*** | | *qFlw6.3* | 6.3 | TQ-SY | FLW | 2.76 | 0.4 | 2.6 |
|  | | *qGnp6.7a* | 6.7 | TQ-SY | GNP | 2.22 | 28.8 | 5.6 |
|  | | *qFlw7.7* | 7.7 | TQ-BJ | FLW | 2.56 | 0.4 | 1.9 |
|  | |  |  | TQ-SY | FLW | 3.40 | 0.6 | 3.7 |
| ***qFll3.12*** | | *qFll8.4* | 8.4 | TQ-BJ | FLL | 9.89 | 2.7 | 12.4 |
|  | |  |  | TQ-SY | FLL | 5.36 | 3.7 | 25.6 |
|  | | *qFlw8.4* |  | TQ-BJ | FLW | 13.60 | 0.9 | 14.8 |
| ***qGnp3.12*** | | *qGnp8.4a* |  | TQ-BJ | GNP | 4.35 | -16.5 | 6.1 |
|  | |  |  | TQ-SY | GNP | 3.35 | -19.4 | 7.6 |
|  | | *qGw8.4* |  | TQ-BJ | GW | 25.77 | 2.0 | 32.1 |
|  | |  |  | TQ-SY | GW | 5.87 | 0.9 | 7.3 |
|  | | *qFlw9.1* | 9.1 | TQ-BJ | FLW | 2.51 | -0.7 | 1.6 |
|  | | *qFll9.7* | 9.7 | TQ-BJ | FLL | 5.12 | -2.0 | 6.3 |
|  | | *qGw9.7* |  | TQ-BJ | GW | 14.90 | -1.7 | 14.0 |
|  | |  |  | TQ-SY | GW | 15.97 | -1.8 | 27.5 |
|  | | *qGy9.7* |  | TQ-BJ | GY | 8.12 | -4.8 | 12.4 |
|  | |  |  | TQ-SY | GY | 2.59 | -2.5 | 4.2 |
|  | | *qFlw10.3* | 10.3 | TQ-BJ | FLW | 3.45 | 0.4 | 2.6 |
|  | |  |  | TQ-SY | FLW | 5.85 | 0.7 | 7.2 |
|  | | *qFlw10.6* | 10.6 | TQ-SY | FLW | 10.05 | -0.8 | 11.2 |
|  | | *qGnp10.6* |  | TQ-SY | GNP | 10.35 | -22.3 | 12.9 |
| ***qFll3.12*** | | *qFll11.3* | 11.3 | TQ-BJ | FLL | 7.21 | 2.5 | 13.2 |
| ***qFlw4.7*** | | *qFlw11.3* |  | TQ-BJ | FLW | 3.53 | 0.7 | 5.4 |
|  | |  |  | TQ-SY | FLW | 2.19 | 0.8 | 6.5 |
|  | | *qFlw11.7* | 11.7 | TQ-SY | FLW | 4.02 | 0.5 | 4.9 |
| ***qFlw4.7*** | | *qFlw12.2a* | 12.2 | TQ-SY | FLW | 7.66 | 0.8 | 6.5 |
|  | | *qFlw1.8* | 1.8 | LT-BJ | FLW | 5.18 | -0.8 | 12.0 |
| ***qFlw4.7*** | | *qFlw2.2* | 2.2 | LT-BJ | FLW | 2.67 | 0.4 | 3.9 |
|  | |  |  | LT-SY | FLW | 4.03 | 0.6 | 7.3 |
|  | | *qFll2.9* | 2.9 | LT-SY | FLL | 3.34 | -1.4 | 6.5 |
|  | | *qGnp2.9* |  | LT-SY | GNP | 5.43 | -18.5 | 9.8 |
| ***qGnp3.12*** | | *qGnp3.5* | 3.5 | LT-BJ | GNP | 8.87 | -25.3 | 12.7 |
| ***SS2*** | | *qFll3.12* | 3.12 | LT-BJ | FLL | 12.81 | -3.0 | 27.8 |
|  | |  |  | LT-SY | FLL | 8.83 | -2.2 | 23.5 |
| ***SS2*** | | *qGnp3.12* |  | LT-BJ | GNP | 9.59 | -14.6 | 11.6 |
|  | |  |  | LT-SY | GNP | 7.36 | -21.5 | 12.5 |
| ***SS2*** | | *qGw3.12a* |  | LT-BJ | GW | 6.69 | 1.1 | 13.8 |
|  | |  |  | LT-SY | GW | 2.78 | 1.0 | 9.5 |
| ***SS2*** | | *qGy3.12* |  | LT-BJ | GY | 3.17 | -2.7 | 6.5 |
|  | |  |  | LT-SY | GY | 7.18 | -3.5 | 12.9 |
| ***SS1*** | | *qFlw4.7* | 4.7 | LT-BJ | FLW | 18.19 | 1.4 | 37.4 |
|  | |  |  | LT-SY | FLW | 6.12 | 1.0 | 15.6 |
| ***SS1*** | | *qGnp4.7* |  | LT-BJ | GNP | 14.62 | 21.3 | 23.0 |
|  | |  |  | LT-SY | GNP | 6.10 | 26.2 | 12.2 |
| ***SS1*** | | *qGw4.7* |  | LT-BJ | GW | 6.04 | -1.2 | 13.1 |
|  | |  |  | LT-SY | GW | 3.42 | -1.0 | 14.5 |
|  | | *qFlw5.5* | 5.5 | LT-BJ | FLW | 3.64 | -0.7 | 11.3 |
|  | |  |  | LT-BJ | GW | 2.66 | -0.7 | 5.7 |
| ***qGnp4.7/ qGnp3.12*** | | *qGnp6.3* | 6.3 | LT-SY | GNP | 4.84 | -17.2 | 7.2 |
| ***qFll3.12*** | | *qFll6.7* | 6.7 | LT-SY | FLL | 4.27 | 1.4 | 10.8 |
| ***qGnp3.12*** | | *qGnp6.7b* |  | LT-SY | GNP | 5.37 | -17.5 | 8.7 |
|  | | *qFlw7.1* | 7.1 | LT-BJ | FLW | 4.27 | -0.5 | 6.4 |
| ***qGnp4.7*** | | *qGnp8.4b* | 8.4 | LT-BJ | GNP | 4.97 | 14.4 | 6.4 |
|  | |  |  | LT-SY | GNP | 2.62 | 14.6 | 6.5 |
|  | | *qGy8.4* |  | LT-BJ | GY | 5.43 | -4.1 | 8.2 |
|  | |  |  | LT-SY | GY | 4.09 | -3.8 | 9.1 |
|  | | *qFll9.3* | 9.3 | LT-BJ | FLL | 2.52 | 1.4 | 5.4 |
|  | | *qGy9.3* |  | LT-BJ | GY | 2.48 | -2.0 | 3.6 |
|  | | *qGnp9.7* | 9.7 | LT-SY | GNP | 4.11 | 16.4 | 6.4 |
|  | | *qFlw12.2b* | 12.2 | LT-SY | FLW | 2.82 | -0.7 | 6.2 |
|  | | *qGy12.2* |  | LT-BJ | GY | 6.38 | 4.6 | 14.1 |
| GB independent QTLs | | |  |  |  |  |  |  |
|  | *qFlw6.5* | | 6.5 | TQ-SY | FLW | 6.27 | -0.7 | 8.9 |
|  |  | |  | LT-SY | FLW | 4.85 | -0.6 | 8.7 |
|  | *qGw7.5* | | 7.5 | TQ-BJ | GW | 2.76 | 0.5 | 4.2 |
|  |  | |  | LT-BJ | GW | 2.89 | 0.7 | 7.1 |
|  | *qFll12.4* | | 12.4 | TQ-BJ | FLL | 5.82 | -2.1 | 6.5 |
|  |  | |  | LT-BJ | FLL | 5.82 | -2.1 | 10.0 |
|  |  | |  | LT-SY | FLL | 3.09 | -1.4 | 8.4 |
|  | *qFlw12.4* | |  | TQ-BJ | FLW | 3.81 | 0.9 | 7.7 |
|  |  | |  | LT-BJ | FLW | 4.75 | 0.8 | 11.8 |
|  |  | |  | LT-SY | FLW | 7.77 | 1.1 | 19.5 |

^a^ SSR and SNP markers defining bins and their physical locations on each chromosome are shown in Table S1 and Figure S1.

^b^ The underlined QTL were also detected in pairwise epistasis (Tables S7 and Figure S1).

^c^ The SSR and SNP markers defining each of the bins and their physical locations in the rice genome are shown in Table S1 QTL.

^d^ The QTL additive effect results from substitution of a Teqing (TQ) allele by a Lemont (LT) allele.
