# Supplementary material for: SS1 (NAL1)- and SS2-Mediated Genetic Networks Underlying Source-Sink and Yield Traits in Rice (Oryza sativa L.)
Source: PLoS One. 2015 Jul 10;10(7):e0132060. doi: 10.1371/journal.pone.0132060 (PMC4498882; doi:10.1371/journal.pone.0132060)
Supplement: S7 Table — (DOC) [file pone.0132060.s015.doc]

**S7 Table** Eighty-seven pairwise epistatic interactions between 46 QTL and estimated pathway effects “*a*” on flag leaf width (FLW, in mm) and length (FLL, in cm), grain number per panicle (GNP), grain yield per plant (GY, in g), and 1000-grain weight (GW, in g) detected in the Lemont (LT-ILs) and Teqing (TQ-ILs) reciprocal introgression lines in Beijing and Sanya

|  |  |  | |  | Beijing | | | | | | Sanya | | | | | |  |  |
| --- | --- | --- | --- | --- | --- | --- | --- | --- | --- | --- | --- | --- | --- | --- | --- | --- | --- | --- |
| Interacting QTL ^a^ | | | | Pop. | Mean trait values of digenic genotypes | | | | *P* | *a* | Mean trait values of digenic genotype | | | | *P* | *a* | *IFA* | FD |
| ***#*** | QTL*_i_* | | QTL*_j_* |  | LT/LT | LT/TQ | TQ/LT | TQ/TQ |  |  | LT/LT | LT/TQ | TQ/LT | TQ/TQ |  |  |  |  |
| 1 | ***qFlw4.7*** | | *qFlw2.2* | LT-ILs | **21.4** | 20.4 | 19.6 | 19.3 | 0.0129 | 1.0 | **20.4** | 19.0 | 18.8 | 18.0 | 0.0469 | 1.4 | LT/LT | **I** |
| 2 | ***qFlw4.7*** | | *qFlw3.5* | TQ-ILs | **22.4** | 18.8 | 17.6 | 17.4 | 0.0013 | 3.6 | **23.3** | 18.3 | 16.8 | 16.7 | <0.0001 | 5.0 | LT/LT | **I** |
| 3 | ***qFlw4.7*** | | *qFlw11.3* | TQ-ILs | **22.4** | 18.9 | 17.9 | 17.4 | 0.0049 | 3.5 | **23.3** | 18.3 | 17.5 | 16.6 | <0.0001 | 5.0 | LT/LT | **I** |
| 4 | ***qFlw4.7*** | | *qFlw6.3* | TQ-ILs | **22.4** | 19.0 | 18.4 | 17.3 | 0.0171 | 3.4 | **23.3** | 18.3 | 17.6 | 16.6 | <0.0001 | 5.0 | LT/LT | **I** |
| 5 | ***qFlw4.7*** | | *qFlw6.6* | TQ-ILs | **22.4** | 19.0 | 18.1 | 17.4 | 0.0095 | 3.4 | **23.3** | 18.4 | 17.0 | 16.7 | <0.0001 | 4.9 | LT/LT | **I** |
| 6 | ***qFlw4.7*** | | *qFlw2.4* | TQ-ILs | **21.4** | 18.7 | 17.7 | 17.4 | 0.004 | 2.7 | **21.2** | 18.2 | 16.7 | 16.7 | 0.0004 | 3.0 | LT/LT | **I** |
| 7 | *qFlw3.5* | | *qFlw6.6* | TQ-ILs | **22.4** | 18.6 | 18.1 | 17.4 | 0.0041 | 3.8 | **23.3** | 17.8 | 17.0 | 16.8 | <0.0001 | 5.5 | LT/LT | **I** |
| 8 | *qFlw2.2* | | *qFlw3.5* | TQ-ILs | 15.9 | 17.7 | **21.5** | 17.4 | <0.0001 | 5.6 | 15.4 | 17.2 | **20.7** | 16.7 | <0.0001 | 5.2 | LT/TQ | **I** |
| 9 | *qFlw2.4* | | *qFlw3.5* | TQ-ILs | **21.0** | 17.8 | 18.0 | 17.4 | 0.0016 | 3.0 | **20.8** | 16.7 | 17.3 | 16.8 | <0.0001 | 3.5 | LT/LT | **I** |
| 10 | *qFlw3.5* | | *qFlw11.3* | TQ-ILs | **22.4** | 18.6 | 17.9 | 17.4 | 0.0021 | 3.8 | **23.3** | 17.8 | 17.0 | 16.8 | <0.0001 | 5.5 | LT/LT | **I** |
| 11 | *qFlw2.2* | | *qFlw6.3* | TQ-ILs | 16.6 | 17.7 | **19.0** | 17.3 | <0.0001 | 2.4 | 16.2 | 17.1 | **18.0** | 16.6 | <0.0001 | 1.8 | TQ/LT | **I** |
| 12 | *qFlw2.4* | | *qFlw6.3* | TQ-ILs | **22.1** | 17.4 | 17.5 | 17.4 | <0.0001 | 4.7 | **21.8** | 16.7 | 16.9 | 16.7 | <0.0001 | 5.1 | LT/LT | **II** |
| 13 | *qFlw6.3* | | *qFlw11.3* | TQ-ILs | **22.4** | 17.6 | 17.0 | 17.4 | <0.0001 | 5.0 | **20.6** | 17.0 | 16.8 | 16.7 | <0.0001 | 3.8 | LT/LT | **II** |
| 14 | *qFlw2.2* | | *qFlw6.6* | TQ-ILs | 17.0 | 17.5 | **18.1** | 17.4 | 0.0437 | 1.1 | 14.9 | 17.1 | **17.5** | 16.7 | 0.0004 | 2.6 | TQ/LT | **I** |
| 15 | *qFlw2.4* | | *qFlw6.6* | TQ-ILs | **19.9** | 17.5 | 17.3 | 17.4 | <0.0001 | 2.5 | **19.5** | 16.4 | 16.0 | 16.8 | <0.0001 | 3.1 | LT/LT | **II** |
| 16 | *qFlw6.6* | | *qFlw11.3* | TQ-ILs | **21.3** | 17.7 | 17.1 | 17.5 | <0.0001 | 3.9 | **21.4** | 15.9 | 16.9 | 16.8 | <0.0001 | 4.9 | LT/LT | **II** |
| 17 | ***qFlw4.7*** | | *qFlw12.2a* | LT-ILs | 21.3 | **22.6** | 19.5 | 19.3 | 0.0033 | 1.3 | 20.2 | **22.0** | 18.9 | 18.0 | <0.0001 | 1.8 | LT/TQ | **I** |
| 18 | ***qGnp4.7*** | | *qGnp2.2* | LT-ILs | 145 | **174** | 122 | 133 | 0.0394 | 29 | 178 | **195** | 167 | 170 | 0.0417 | 17 | LT/TQ | **I** |
| 19 | *qGy4.7* | | *qGy2.2* | LT-ILs | 21.7 | 21.6 | 22.7 | **27.9** | 0.0204 | 5.9 | 15.8 | 13.4 | 16.7 | **28.2** | <0.0001 | 12.9 | TQ/TQ | **I** |
| 20 | ***qGnp4.7*** | | *qGnp8.4b* | LT-ILs | 143 | **183** | 126 | 140 | 0.0283 | 39 | 157 | **191** | 158 | 158 | 0.0081 | 34 | LT/TQ | **I** |
| 21 | *qGy4.7* | | *qGy8.4* | LT-ILs | 21.5 | 25.5 | 20.1 | **33.6** | 0.0061 | 10.9 | 16.0 | 14.7 | 14.8 | **28.4** | <0.0001 | 13.2 | TQ/TQ | **I** |
| 22 | ***qGnp4.7*** | | *qGnp1.8* | TQ-ILs |  |  |  |  |  |  | 211 | **251** | 216 | 218 | 0.0361 | 40 | LT/TQ | **I** |
| 23 | ***qGnp4.7*** | | *qGnp1.8* | LT-ILs | 144 | **161** | 138 | 137 | 0.0372 | 17 |  |  |  |  |  |  | LT/TQ | **I** |
| 24 | *qGy4.7* | | *qGy1.8* | LT-ILs | 21.7 | 22.3 | 23.8 | **29.6** | 0.0310 | 7.0 | 15.9 | 15.1 | 16.5 | **22.7** | 0.0088 | 6.9 | TQ/TQ | **I** |
| 25 | ***qGnp4.7*** | | *qGnp6.3* | LT-ILs | 144 | **192** | 145 | 114 | <0.0001 | 47.3 | 179 | **240** | 164 | 162 | 0.0015 | 61 | LT/TQ | **I** |
| 26 | *qGy4.7* | | *qGy6.3* | LT-ILs | 22.0 | 21.3 | 20.3 | **36.8** | <0.0001 | 15.6 | 15.9 | 13.6 | 16.0 | **29.7** | <0.0001 | 14.5 | TQ/TQ | **I** |
| 27 | ***qGnp4.7*** | | *qGnp4.1* | LT-ILs | 143.8 | **173** | 129 | 134 | 0.0283 | 29 | 155 | **212** | 160 | 139 | 0.002 | 57 | LT/TQ | **I** |
| 28 | *qGy4.7* | | *qGy4.1* | LT-ILs | 22.1 | 22.4 | 23.1 | **35.8** | 0.0029 | 13.2 | 16.2 | 14.5 | 16.9 | **32.1** | <0.0001 | 16.2 | TQ/TQ | **I** |
| 29 | *qGnp1.8* | | *qGnp6.3* | LT-ILs | 143 | 136 | 150 | **181** | 0.0019 | 38 | 178 | 185 | 176 | **252** | 0.0002 | 73 | TQ/TQ | **II** |
| 30 | *qGnp1.8* | | *qGnp4.1* | LT-ILs | 142 | 155 | 142 | **195** | 0.0020 | 40 | 176 | 189 | 174 | **230** | 0.0214 | 41 | TQ/TQ | **I** |
| 31 | *qGnp4.1* | | *qGnp6.3* | LT-ILs | 143 | 129 | 160 | **197** | 0.0003 | 37 | 177 | 157 | 186 | **226** | 0.0024 | 40 | TQ/TQ | **I** |
| 32 | *qGy4.7* | | *qGy6.6* | LT-ILs | 21.9 | 23.2 | 23.4 | **32.9** | 0.0350 | 10.1 | 16.6 | 11.8 | 18.1 | **26.4** | 0.0005 | 10.9 | TQ/TQ | **I** |
| 33 | *qGy4.7* | | *qGy2.4* | LT-ILs | 21.8 | 23.2 | 19.8 | **35.1** | 0.0002 | 13.5 | 15.8 | 16.7 | 16.4 | **27.1** | 0.0061 | 10.8 | TQ/TQ | **I** |
| 34 | *qGw4.7* | | *qGw2.4* | TQ-ILs | **27.0** | 22.0 | 22.5 | 22.0 | <0.0001 | 2.4 | **28.4** | 23.2 | 23.8 | 23.4 | <0.0001 | 2.5 | LT/LT | **I** |
| 35 | ***qFll3.12*** | | *qFll3.5* | LT-ILs |  |  |  |  |  |  | 23.2 | 24.2 | **28.9** | 24.8 | <0.0001 | 4.1 | TQ/LT | **I** |
| 36 | ***qFll3.12*** | | *qFll11.3* | LT-ILs | 29.8 | 28.4 | **36.2** | 33.2 | 0.0339 | 3.0 | 23.1 | 23.9 | **28.0** | 25.1 | <0.0001 | 2.9 | TQ/LT | **I** |
| 37 | ***qFll3.12*** | | *qFll11.3* | TQ-ILs |  |  |  |  |  |  | 22.7 | 22.5 | **28.5** | 26 | 0.0432 | 2.5 | TQ/LT | **I** |
| 38 | ***qFll3.12*** | | *qFll1.8* | LT-ILs | 29.5 | 29.9 | **36.0** | 32.7 | <0.0001 | 3.3 | 23.1 | 22.8 | **27.4** | 25.2 | 0.0041 | 2.2 | TQ/LT | **I** |
| 39 | ***qFll3.12*** | | *qFll2.6* | LT-ILs | 29.5 | 32.2 | **36.5** | 32.7 | <0.0001 | 3.8 | 23.3 | 24.8 | **27.8** | 25.5 | <0.0001 | 2.3 | TQ/LT | **I** |
| 40 | ***qFll3.12*** | | *qFll8.4* | LT-ILs |  |  |  |  |  |  | 23.2 | 23.7 | **27.5** | 25.9 | 0.0194 | 1.6 | TQ/LT | **I** |
| 41 | ***qFll3.12*** | | *qFll8.4* | TQ-ILs | 23.4 | 24.4 | **33.7** | 29.4 | 0.0003 | 4.3 |  |  |  |  |  |  | TQ/LT | **I** |
| 42 | ***qFll3.12*** | | *qFll6.7* | LT-ILs | 29.5 | 29.9 | **36.5** | 34 | 0.0006 | 2.5 | 23.1 | 24.4 | **28.1** | 25.7 | <0.0001 | 2.4 | TQ/LT | **I** |
| 43 | *qFll3.5* | | *qFll11.3* | TQ-ILs | **34.6** | 29.8 | 27.9 | 29.4 | 0.0029 | 4.8 | **31.7** | 27.9 | 24.6 | 25.9 | 0.0043 | 3.8 | LT/LT | **I** |
| 44 | *qFll1.8* | | *qFll2.6* | TQ-ILs | **38.6** | 31.5 | 30.8 | 28.7 | <0.0001 | 7.1 | **30.6** | 27.8 | 25.8 | 25.6 | 0.0093 | 2.8 | LT/LT | **I** |
| 45 | *qFll1.8* | | *qFll8.4* | TQ-ILs | **36.9** | 32.2 | 30.9 | 28.6 | 0.0394 | 4.7 | **32.8** | 27.2 | 25.8 | 25.6 | <0.0001 | 5.5 | LT/LT | **I** |
| 46 | *qFll2.6* | | *qFll8.4* | TQ-ILs | **43.0** | 31.7 | 31.1 | 28.8 | <0.0001 | 12.5 | **32.0** | 26.1 | 25.9 | 25.8 | <0.0001 | 6.1 | LT/LT | **II** |
| 47 | ***qGnp3.12*** | | *qGnp3.5* | LT-ILs | 138 | 135 | 178 | **218** | 0.0037 | 40 | 175 | 169 | 209 | **271** | 0.0067 | 61 | TQ/TQ | **I** |
| 48 | ***qGy3.12*** | | *qGy3.5* | LT-ILs | 21.2 | 22.3 | 22.4 | **36.7** | <0.0001 | 14.7 | 15.7 | 16.0 | 14.5 | **22.3** | 0.0125 | 6.9 | TQ/TQ | **I** |
| 49 | ***qGnp3.12*** | | *qGnp1.8* | LT-ILs | 138 | 137 | 177 | **193** | 0.0107 | 16 | 178 | 159 | 201 | **259** | <0.0001 | 58 | TQ/TQ | **I** |
| 50 | ***qGy3.12*** | | *qGy1.8* | LT-ILs | 21.2 | 22.9 | 23.4 | **34.8** | 0.0041 | 12.3 |  |  |  |  |  |  | TQ/TQ | **I** |
| 51 | ***qGnp3.12*** | | *qGnp4.1* | LT-ILs | 140 | 141 | 162 | **195** | 0.0062 | 32 |  |  |  |  |  |  | TQ/TQ | **I** |
| 52 | ***qGy3.12*** | | *qGy4.1* | LT-ILs | 21.6 | 20.7 | 25.8 | **34.3** | 0.0208 | 8.5 | 16.2 | 14.4 | 16.3 | **29.7** | 0.0002 | 13.4 | TQ/TQ | **I** |
| 53 | ***qGy3.12*** | | *qGy11.3* | LT-ILs | 21.3 | 21.7 | 24.7 | **32.7** | 0.0269 | 10.1 | 15.5 | 16.9 | 14.6 | **28.9** | <0.0001 | 13.2 | TQ/TQ | **I** |
| 54 | ***qGnp3.12*** | | *qGnp8.4a* | LT-ILs | 139 | 145 | **176** | 145 | <0.0001 | 31 | 174 | 180 | **223** | 198 | 0.0376 | 25 | TQ/LT | **I** |
| 55 | ***qGy3.12*** | | *qGy8.4* | LT-ILs | 21.2 | 23.3 | 23.6 | **34.0** | 0.0339 | 11.3 | 15.7 | 16.2 | 15.2 | **28.3** | 0.0013 | 12.6 | TQ/TQ | **II** |
| 56 | ***qGw3.12b*** | | *qGw8.4* | LT-ILs | 22.5 | 22.7 | 20.7 | **24.3** | <0.0001 | 2.3 | 22.3 | 22.8 | 22.0 | **24.5** | 0.0083 | 2.1 | TQ/TQ | **II** |
| 57 | ***qGnp3.12*** | | *qGnp6.7b* | LT-ILs | 140 | 135 | 157 | **185** | <0.0001 | 27.7 | 176 | 175 | 208 | **225** | 0.0430 | 17 | TQ/TQ | **I** |
| 58 | ***qGy3.12*** | | *qGy6.7* | LT-ILs | 21.4 | 20.2 | 22.4 | **33.6** | <0.0001 | 11.2 | 15.4 | 16.4 | 14.6 | **25.6** | 0.0005 | 11.0 | TQ/TQ | **I** |
| 59 | ***qGnp3.12*** | | *qGnp5.5* | LT-ILs | 139 | 144 | **170** | 139 | 0.0060 | 31 | 175 | 177 | **220** | 180 | 0.0422 | 40 | TQ/LT | **I** |
| 60 | *qGw3.12a* | | *qGw4.1* | TQ-ILs | **23.4** | 22.3 | 21.6 | 22.0 | 0.0169 |  | **24.8** | 22.8 | 23.6 | 23.4 | 0.0053 |  | **LT/LT** | **II** |
| 61 | ***qGw3.12b*** | | *qGw5.5* | LT-ILs | 22.5 | 22.0 | 21.6 | **23.7** | 0.0189 | 1.7 | 22.4 | 21.9 | 22.5 | **24.6** | 0.0179 | 2.3 | TQ/TQ | **II** |
| 62 | ***qGnp3.12*** | | *qGnp6.3* | LT-ILs | 139 | 128 | 174 | **204** | 0.0044 | 30 | 175 | 163 | 203 | **247** | 0.0194 | 44 | TQ/TQ | **I** |
| 63 | ***qGy3.12*** | | *qGy6.3* | LT-ILs | 21.5 | 19.3 | 23.5 | **32.3** | 0.0011 | 8.8 | 15.9 | 14.1 | 15.7 | **24.0** | 0.0035 | 8.3 | TQ/TQ | **I** |
| 64 | *qGw3.12a* | | *qGw1.8* | LT-ILs | **24.1** | 22.2 | 21.4 | 21.9 | 0.0105 | 1.3 |  |  |  |  |  |  | LT/LT | **II** |
| 65 | *qGy1.8* | | *qGy2.2* | LT-ILs | 21.7 | 20.3 | 21.5 | **26.8** | 0.0256 | 5.6 | 15.7 | 15.3 | 16.9 | **23.5** | 0.0435 | 7.5 | TQ/TQ | **II** |
| 66 | *qGy1.8* | | *qGy2.4* | LT-ILs | 21.4 | 26.5 | 21.7 | **39.7** | 0.0015 | 16.5 | 15.9 | 17.2 | 16.1 | **28.7** | 0.0009 | 12.3 | TQ/TQ | **I** |
| 67 | *qGy1.8* | | *qGy4.1* | LT-ILs | 22.5 | 25.0 | 21.6 | **40.0** | 0.0117 | 17.0 | 16.7 | 16.9 | 14.1 | **32.1** | <0.0001 | 16.2 | TQ/TQ | **I** |
| 68 | *qGy1.8* | | *qGy6.3* | LT-ILs | 22.0 | 19.7 | 22.7 | **26.7** | 0.0179 | 5.2 | 16.5 | 15.2 | 12.0 | **28.0** | <0.0001 | 13.4 | TQ/TQ | **I** |
| 69 | *qGy1.8* | | *qGy6.6* | LT-ILs | 22.2 | 24.9 | 21.7 | **34.7** | 0.0027 | 11.8 | 16.9 | 18.3 | 13.6 | **27.8** | 0.0068 | 11.5 | TQ/TQ | **I** |
| 70 | *qGy2.2* | | *qGy4.1* | LT-ILs | 21.8 | 24.6 | 21.7 | **34.4** | 0.0027 | 11.7 | 16.5 | 14.6 | 14.5 | **49.8** | <0.0001 | 34.6 | TQ/TQ | **I** |
| 71 | *qGy2.2* | | *qGy6.3* | LT-ILs | 21.9 | 19.4 | 20.9 | **29.2** | 0.0008 | 8.5 | 16.1 | 13.9 | 14.0 | **31.6** | <0.0001 | 16.9 | TQ/TQ | **II** |
| 72 | *qGy2.2* | | *qGy6.6* | LT-ILs | 21.8 | 23.9 | 20.2 | **29.9** | 0.0047 | 7.9 | 16.6 | 14.5 | 18.6 | **24.9** | 0.0394 | 8.3 | TQ/TQ | **II** |
| 73 | *qGy1.8* | | *qGy8.4* | LT-ILs | 21.5 | 26.1 | 20.3 | **35.0** | 0.0053 | 12.4 | 16.0 | 17.7 | 14.8 | **28.9** | 0.0004 | 12.7 | TQ/TQ | **I** |
| 74 | *qGy2.2* | | *qGy8.4* | LT-ILs | 21.5 | 25.1 | 20.2 | **30.3** | 0.0189 | 8.0 | 15.8 | 16.5 | 14.2 | **32.9** | <0.0001 | 17.4 | TQ/TQ | **I** |
| 75 | *qGy2.4* | | *qGy4.1* | LT-ILs | 21.7 | 21.2 | **27.2** | **43.9** | <0.0001 | 20.5 | 16.4 | 15.4 | 18.8 | **39.4** | <0.0001 | 22.5 | TQ/TQ | **I** |
| 76 | *qGy2.4* | | *qGy6.3* | LT-ILs | 21.8 | 19.6 | 22.6 | **39.1** | <0.0001 | 17.8 | 16.0 | 15.5 | 17.1 | **37.7** | <0.0001 | 21.5 | TQ/TQ | **II** |
| 77 | *qGy2.4* | | *qGy8.4* | LT-ILs | 21.5 | 25.7 | 21.2 | **44.7** | <0.0001 | 21.9 | 15.7 | 18.8 | 17.4 | **32.1** | 0.0041 | 14.8 | TQ/TQ | **I** |
| 78 | *qGy4.1* | | *qGy6.6* | LT-ILs | 22.1 | 21.8 | 22.7 | **39.5** | <0.0001 | 17.3 | 16.7 | 15.8 | 19.8 | **27.4** | 0.0062 | 10.0 | TQ/TQ | **II** |
| 79 | *qGy4.1* | | *qGy8.4* | LT-ILs | 21.2 | 27.6 | 20.5 | **37.1** | 0.0160 | 14.0 | 16.2 | 17.7 | 14.1 | **35.2** | <0.0001 | 19.2 | TQ/TQ | **I** |
| 80 | *qGy6.3* | | *qGy6.6* | LT-ILs | 21.8 | 22.6 | 23.7 | **37.8** | 0.0010 | 15.1 | 16.5 | 13.1 | 18.1 | **28.6** | 0.0007 | 12.7 | TQ/TQ | **I** |
| 81 | *qGy4.1* | | *qGy6.3* | LT-ILs | 21.9 | 26.4 | 21.7 | **34.3** | 0.0139 | 11.0 | 16.4 | 13.5 | 12.1 | **30.0** | <0.0001 | 16.0 | TQ/TQ | **I** |
| 82 | *qGy6.3* | | *qGy8.4* | LT-ILs | 21.6 | 24.0 | 20.9 | **38.7** | 0.0002 | 16.5 | 16.0 | 17.3 | 13.4 | **35.8** | <0.0001 | 20.2 | TQ/TQ | **I** |
| 83 | *qGy6.3* | | *qGy6.7* | LT-ILs | 21.8 | 21.5 | 19.7 | **31.7** | 0.0005 | 10.7 | 16.0 | 17.0 | 14.7 | **29.9** | <0.0001 | 14.0 | TQ/TQ | **II** |
| 84 | *qGy6.6* | | *qGy6.7* | LT-ILs | 21.7 | 23.2 | 19.0 | **32.0** | 0.0034 | 10.7 | 16.3 | 18.6 | 10.1 | **23.2** | 0.0039 | 8.2 | TQ/TQ | **I** |
| 85 | *qGy6.7* | | *qGy11.3* | LT-ILs | 22.1 | 23.3 | 19.6 | **30.0** | 0.0072 | 8.3 | 15.6 | 14.0 | 14.9 | **28.0** | <0.0001 | 13.2 | TQ/TQ | **II** |
| 86 | *qGy6.7* | | *qGy8.4* | LT-ILs | 21.1 | 26.4 | 23.4 | **42.1** | 0.0012 | 18.5 | 15.8 | 18.2 | 15.7 | **31.4** | 0.0002 | 14.8 | TQ/TQ | **I** |
| 87 | *qGy8.4* | | *qGy11.3* | LT-ILs | 21.5 | 20.8 | **25.2** | **33.7** | 0.0103 | 11.2 | 15.6 | 16.2 | 15.2 | **29.2** | 0.0004 | 13.5 | TQ/TQ | **I** |

^a^ *P* is the probability for epistasis in the *F* test of the two-way ANOVA analyses. “*a*” is the estimated effect of the downstream pathway from substitution of the nonfunctional genotypes by the functional one [40]. The inferred functional alleles (*IFA*) at QTL*_i_* (on the left) and QTL*_j_* (on the right) were suggested based on the observed phenotypic patterns of the 4 digenic genotypes against their expected ones based on the type of functional dependency (FD) of the theoretical models [40], in which types I and II represent the one-way FD between a downstream QTL on its upstream one and the mutual FD between 2 QTL in the same pathway acting in a complementary manner. The bold numbers are the mean trait values of the predicted functional genotypes with functional alleles at both interacting loci. The underlined trait value(s) suggested the presence of the one-way (downstream on an upstream) functional dependency between the interacting loci [40].
