# Supplementary material for: SS1 (NAL1)- and SS2-Mediated Genetic Networks Underlying Source-Sink and Yield Traits in Rice (Oryza sativa L.)
Source: PLoS One. 2015 Jul 10;10(7):e0132060. doi: 10.1371/journal.pone.0132060 (PMC4498882; doi:10.1371/journal.pone.0132060)
Supplement: S8 Table — (DOC) [file pone.0132060.s016.doc]

**S8 Table** Comparison of 27 main-effect QTL and 46 epistatic QTL for different source-sink (SS) and yield traits detected in the reciprocal introgression populations of Lemont (LT) and Teqing (TQ) in Bejing and Sanya

| Pop.-E ^a^ | QTL |  | AIT | Pop.-E | QTL |  | AIT | Pop.-E | QTL |  | AIT | Pop.-E | QTL |  | AIT | Pop.-E | QTL |  | AIT |
| --- | --- | --- | --- | --- | --- | --- | --- | --- | --- | --- | --- | --- | --- | --- | --- | --- | --- | --- | --- |
| **Main-effect QTL** | | | |  |  |  |  |  |  |  |  |  |  |  |  |  |  |  |  |
| TQ-B | *qFlw7.7* |  | *LT* | TQ-SY | *qFll4.1* |  | *LT* | TQ-SY | *qGnp6.7a* |  | *LT* | TQ-SY | *qGw4.1* |  | *TQ* | *TQ-both* | *qGy9.7* |  | *TQ* |
| TQ-BJ | *qFlw8.4* |  | *LT* | TQ-BJ | *qFll9.7* |  | *TQ* | TQ-SY | *qGnp10.6* |  | *TQ* | *TQ-both* | *qGw9.7* |  | *TQ* | LT-BJ | *qGy9.3* |  | *TQ* |
| TQ-BJ | *qFlw9.1* |  | *TQ* | LT-SY | *qFll2.9* |  | *TQ* | LT-SY | *qGnp2.9* |  | *TQ* | **B-B** | ***qGw7.5*** |  | *LT* | LT-BJ | *qGy12.2* |  | *LT* |
| TQ-BJ | *qFlw10.3* |  | *LT* | LT-BJ | *qFll9.3* |  | *LT* | LT-SY | *qGnp9.7* |  | *LT* |  |  |  |  |  |  |  |  |
| TQ-SY | *qFlw10.6* |  | *TQ* | **B-B** | ***qFll12.4*** |  | *TQ* |  |  |  |  |  |  |  |  |  |  |  |  |
| TQ-SY | *qFlw11.7* |  | *LT* |  |  |  |  |  |  |  |  |  |  |  |  |  |  |  |  |
| LT-BJ | *qFlw1.8* |  | *LT* | **B-B** | ***qFlw6.5*** |  | *TQ* |  |  |  |  |  |  |  |  |  |  |  |  |
| LT-B | *qFlw5.5* |  | *LT* | **B-B** | ***qFlw12.4*** |  | *TQ* |  |  |  |  |  |  |  |  |  |  |  |  |
| LT-BJ | *qFlw7.1* |  | *LT* |  |  |  |  |  |  |  |  |  |  |  |  |  |  |  |  |
| **Epistatic QTL** | | N | IFA | Pop.-E | QTL | N | IFA | Pop.-E | QTL | N | IFA | Pop.-E | QTL | N | IFA | Pop.-E | QTL | N | IFA |
| **B-B** | ***qFlw4.7*** | **7** | ***LT*** | **B-B** | ***qFll3.12*** | **6** | ***TQ*** | **B-B** | ***qGnp3.12*** | **7** | ***TQ*** | **B-B** | ***qGw3.12a*** | **2** | ***LT*** | LT-B | *qGy8.4* | 9 | *TQ* |
| TQ-B | *qFlw6.6* | 5 | *LT* | B-B | *qFll8.4* | 4 | *LT* | **B-B** | ***qGnp4.7*** | **6** | ***LT*** | B-B | *qGw8.4* | 1 | *TQ* | LT-B | *qGy6.3* | 9 | *TQ* |
| TQ-B | *qFlw3.5* | 5 | *TQ* | B-B | *qFll2.6* | 3 | *LT* | B-B | *qGnp1.8* | 5 | *TQ* | LT-B | *qGw5.5* | 1 | *TQ* | B-B | *qGy4.1* | 8 | *TQ* |
| TQ-SY | *qFlw6.3* | 4 | *LT* | B-B | *qFll1.8* | 3 | *LT* | LT-SY | *qGnp6.3* | 4 | *TQ* | B-B | *qGw4.7* | 1 | *LT* | LT-B | *qGy1.8* | 8 | *TQ* |
| TQ-B | *qFlw2.4* | 4 | *LT* | B-B | *qFll3.5* | 2 | *LT* | B-B | *qGnp4.1* | 4 | *TQ* | TQ-B | *qGw2.4* | 1 | *LT* | LT-B | *qGy4.7* | 7 | *TQ* |
| B-B | *qFlw2.2* | 4 | *LT* | B-B | *qFll11.3* | 2 | *LT* | LT-B | *qGnp8.4b* | 1 | *LT* | B-B | *qGw1.8* | 1 | *TQ* | LT-B | *qGy3.12* | 7 | *TQ* |
| TQ-B | *qFlw11.3* | 4 | *LT* | B-B | *qFll6.7* | 1 | *LT* | B-B | *qGnp8.4a* | 1 | *TQ* | B-B | *qGw3.12b* | 2 | *TQ* | LT-B | *qGy6.6* | 6 | *TQ* |
| B-B | *qFlw12.2a* | 1 | *TQ* |  |  |  |  | LT-SY | *qGnp6.7b* | 1 | *TQ* | TQ-B | *qGw4.1* | 1 | *LT* | LT-B | *qGy2.2* | 6 | *TQ* |
|  |  |  |  |  |  |  |  | B-B | *qGnp5.5* | 1 | *LT* |  |  |  |  | LT-B | *qGy6.7* | 5 | *TQ* |
|  |  |  |  |  |  |  |  | LT-B | *qGnp3.5* | 1 | *TQ* |  |  |  |  | LT-B | *qGy2.4* | 5 | *TQ* |
|  |  |  |  |  |  |  |  | LT-B | *qGnp2.2* | 1 | TQ |  |  |  |  | LT-B | *qGy11.3* | 3 | *TQ* |
|  |  |  |  |  |  |  |  |  |  |  |  |  |  |  |  | LT-B | *qGy3.5* | 1 | *TQ* |

^a^ B-B indicates that the QTL were detected in both TQ-ILs and LT-ILs and in both Beijing (BJ) and Sanya (SY) environments. AIT = allele for increased trait values. N is the number of digenic interactions of each E-QTL involved. Underlined E-QTL were detected as M-QTL (Table S6). IFA = the inferred functional allele at that QTL based on the results of Table S7.
