# Supplementary material for: SS1 (NAL1)- and SS2-Mediated Genetic Networks Underlying Source-Sink and Yield Traits in Rice (Oryza sativa L.)
Source: PLoS One. 2015 Jul 10;10(7):e0132060. doi: 10.1371/journal.pone.0132060 (PMC4498882; doi:10.1371/journal.pone.0132060)
Supplement: S9 Table — (DOC) [file pone.0132060.s017.doc]

**S9 Table** Allelic diversity of 144 rice accessions at the position of 698 bp in the coding region of *LOC_Os04g52479* (*NAL1*) gene

| # | Variety | Original Source | TL/MV^a^ | Indica/Japonica | Mutation | FLW |
| --- | --- | --- | --- | --- | --- | --- |
| 1 | Te Qing | China | MV | I | G | 1.36 |
| 2 | Lemont | America | MV | J | A | 1.77 |
| 3 | 343 | China | MV | I | A | 2.11 |
| 4 | Adny 11 | Nigeria | MV | I | G | 1.27 |
| 5 | Amol 3(Sana) | Iran | MV | I | G | 1.16 |
| 6 | Basmati | India | MV | J | G | 1.22 |
| 7 | Basmati 385 | Pakistan | MV | J | G | 1.27 |
| 8 | Bhavani | India | TL | I | G | 1.28 |
| 9 | Binam | Iran | TL | J | G | 1.13 |
| 10 | 117 | China | BL | J | A | 2.49 |
| 11 | Cao Zhao 2 | China | BL | J | G | 1.30 |
| 12 | CAU1 | China | BL | J | A | 1.97 |
| 13 | Chhomrong | Nepal | MV | J | A | 2.08 |
| 14 | CT9993-5-10-1-M | Columbia | TL | J | G | 1.80 |
| 15 | Lin Yi Tang Dao? | China | TL | J | G | 2.02 |
| 16 | SLG 1 | China | TL | I | G | 1.89 |
| 17 | Dacca6 | Philippines | TL | J | G | 1.01 |
| 18 | Doddabyranellu | India | TL | I | G | 1.23 |
| 19 | Domsiah | Iran | TL | J | G | 1.19 |
| 20 | F88 | China | MV | J | A | 2.72 |
| 21 | Gajale | Nepal | TL | J | G | 1.30 |
| 22 | Ginga | Japan | MV | J | G | 1.15 |
| 23 | IR47686-6-2-1-1 | Philippines | BL | I | A | 1.80 |
| 24 | IR68897B | Philippines | BL | I | G | 1.17 |
| 25 | IR69428-6-1-1-3-3 | Philippines | BL | I | G | 2.00 |
| 26 | IR8192-200-3-3-1-1 | Philippines | BL | I | G | 1.26 |
| 27 | IRAT 352 | -  Columbia | BL | J | A | 2.01 |
| 28 | JWR 221 | China | BL | J | G | 2.15 |
| 29 | Karnal Local | India | TL | J | G | 1.17 |
| 30 | Khao Dawk Mali 105 | Thailand | TL | I | G | 1.28 |
| 31 | Khasar | India | MV | I | G | 1.07 |
| 32 | LX2007 | China | BL | I | G | 1.88 |
| 33 | Milagrosa | Philippines | TL | I | G | 1.47 |
| 34 | Milyang23 | Korea | MV | I | G | 1.06 |
| 35 | MOLOK | Indonesia | TL | J | A | 1.82 |
| 36 | MR167 | Malaysia | MV | I | G | 1.22 |
| 37 | MR39 | Malaysia | MV | I | A | 1.91 |
| 38 | Nionoka | France | MV | I | G | 1.26 |
| 39 | Nipponbare | Japan | MV | J | A | 1.08 |
| 40 | Padi Siam Kuning | Indonesia | TL | I | G | 2.05 |
| 41 | PsBRC28 | Philippines | MV | I | G | 1.26 |
| 42 | PSBRC82 | Philippines | MV | I | G | 1.23 |
| 43 | Pathumthani 60 | Thailand | MV | I | G | 1.83 |
| 44 | Ptb33 | India | TL | I | G | 1.16 |
| 45 | Pusa(Basmati 1) | India | MV | I | G | 1.15 |
| 46 | Qb_604 | China | BL | I | G | 1.29 |
| 47 | R402 | China | MV | J | A | 1.25 |
| 48 | R644 | China | MV | J | A | 1.80 |
| 49 | Sai Bui Bao | Vietnam | TL | J | G | 1.85 |
| 50 | SUPHANBURI 90 | Thailand | MV | I | G | 2.03 |
| 51 | Tarom molaii | Iran | TL | J | G | 1.17 |
| 52 | Tetep | Vietnam | TL | I | G | 1.18 |
| 53 | TKM6 | India | MV | I | G | 1.29 |
| 54 | UP15 | Japan | TL | J | G | 1.19 |
| 55 | UPR 191-66 | India | BL | I | G | 1.23 |
| 56 | W1263 | India | MV | I | G | 1.12 |
| 57 | WH26 | China | MV | I | A | 1.86 |
| 58 | Yuan Jing 7 | China | MV | J | G | 1.29 |
| 59 | Yun Hui72 | China | MV | J | G | 1.89 |
| 60 | Z3 | China | MV | I | G | 2.06 |
| 61 | Ai Jiao Nan Te | China | MV | J | G | 2.01 |
| 62 | Ai Tuo Guo 151 | China | TL | J | G | 2.28 |
| 63 | Ai Zi Zhan | China | MV | I | G | 1.60 |
| 64 | Ba Wang Bian 1 | China | TL | J | G | 2.31 |
| 65 | Bai Ke Hua Lu | China | TL | J | G | 1.95 |
| 66 | Ban Bang Guo | China | TL | J | G | 1.93 |
| 67 | Cheng Duo Ai 3 | China | MV | I | G | 1.96 |
| 68 | Cheng Hui 448 | China | MV | I | G | 1.90 |
| 69 | Du 57 | China | MV | I | G | 1.85 |
| 70 | Fei Dong Tang Dao | China | TL | I | G | 0.85 |
| 71 | Feng Ai Zhan | China | MV | I | G | 1.84 |
| 72 | Fu 838 | China | MV | I | G | 1.80 |
| 73 | Gang 46B | China | MV | I | G | 1.82 |
| 74 | Guan Dong 194 | China | MV | J | A | 1.05 |
| 75 | Gui Hui 2190 | China | MV | I | G | 2.25 |
| 76 | Gui Huan Zhan | China | MV | I | G | 1.29 |
| 77 | Zhao Dao 502 | China | MV | I | A | 1.92 |
| 78 | Hang 1 | China | MV | I | G | 2.12 |
| 79 | He Geng 2 | China | MV | J | A | 1.24 |
| 80 | Hong Wan 1 | China | MV | I | G | 1.27 |
| 81 | Hu Hui 628 | China | MV | I | G | 2.15 |
| 82 | Hua Yu 560 | China | MV | J | A | 1.26 |
| 83 | Hua 564 | China | MV | I | A | 1.90 |
| 84 | Hua Geng 6 | China | MV | J | A | 1.24 |
| 85 | Jing Xi Bai | China | TL | I | G | 1.13 |
| 86 | Jing Chuan 1 | China | MV | J | A | 1.06 |
| 87 | Lun Hui 422 | China | MV | I | A | 1.80 |
| 88 | Geng 7623 | China | MV | J | A | 1.93 |
| 89 | Ken Dao 12 | China | MV | J | A | 0.98 |
| 90 | Lao Hu Zhong | China | TL | I | A | 1.36 |
| 91 | Long Dao 5 | China | MV | J | A | 1.20 |
| 92 | Long Geng 21 | China | MV | J | A | 1.16 |
| 93 | Long Geng 24 | China | MV | J | A | 1.07 |
| 94 | Lu Hui 17 | China | MV | I | G | 1.81 |
| 95 | Tie Gan Wu | China | TL | I | G | 1.05 |
| 96 | Meng Jia Ding 2 | China | TL | I | G | 2.07 |
| 97 | Kiam Hui 725 | China | MV | I | G | 1.89 |
| 98 | Ming Hui 86 | China | MV | I | G | 2.11 |
| 99 | Mo Xi Qiu | China | TL | I | G | 1.07 |
| 100 | Nan Jing 11 | China | MV | I | G | 2.05 |
| 101 | Nan Geng 44 | China | MV | J | A | 1.14 |
| 102 | Nan Geng 45 | China | MV | J | A | 1.27 |
| 103 | Nan Geng 46 | China | MV | J | A | 0.90 |
| 104 | Nan Hui 21 | China | MV | J | A | 1.91 |
| 105 | Ji Mei | China | TL | I | G | 1.11 |
| 106 | Shan Jiang 1 | China | MV | J | A | 1.23 |
| 107 | Sheng Nong 89366 | China | MV | J | A | 1.84 |
| 108 | Shui Hui 498 | China | MV | I | G | 2.04 |
| 109 | Shu Ya Zhan | China | TL | I | G | 1.11 |
| 110 | Si Miao | China | MV | I | G | 1.40 |
| 111 | Song 01-173 | China | MV | J | A | 1.28 |
| 112 | Song Geng 10 | China | MV | J | A | 0.91 |
| 113 | Song Geng 6 | China | MV | J | A | 1.19 |
| 114 | Shui Geng 10 | China | MV | J | A | 1.10 |
| 115 | Shui Geng 7 | China | MV | J | A | 0.98 |
| 116 | Shui Geng 8 | China | MV | J | A | 1.05 |
| 117 | Shui Geng 9 | China | MV | J | A | 1.05 |
| 118 | Tai Shan Nuo | China | MV | J | G | 2.36 |
| 119 | Te Xian Zhan 25 | China | MV | I | A | 2.00 |
| 120 | Tong He 832 | China | MV | I | A | 1.18 |
| 121 | Wan Xian 763 | China | MV | I | A | 1.81 |
| 122 | Wan Xian 77 | China | MV | I | A | 2.16 |
| 123 | Wu Ke Zhan | China | LD | I | G | 1.17 |
| 124 | Wu Da Dao Zhong | China | MV | I | A | 1.13 |
| 125 | Wu Xiang Geng 14 | China | MV | J | A | 1.29 |
| 126 | Wu Yu Geng 14 | China | MV | J | A | 1.29 |
| 127 | Wu Yu Geng 20 | China | MV | J | A | 1.28 |
| 128 | Wu Yu Geng 3 | China | MV | J | A | 0.97 |
| 129 | Xu Dao 3 | China | MV | J | A | 1.22 |
| 130 | Xu Dao 4 | China | MV | J | A | 1.23 |
| 131 | Xu Dao 5 | China | MV | J | A | 1.28 |
| 132 | Yan Dao 8 | China | MV | J | A | 1.22 |
| 133 | Yan Hui 559 | China | MV | J | G | 1.84 |
| 134 | Ye Tuo Zai | China | LD | I | G | 1.20 |
| 135 | Yi Hui 1577 | China | MV | I | G | 1.81 |
| 136 | You Zhan 8 | China | MV | I | G | 1.29 |
| 137 | Koshihikari | China | MV | J | A | 1.03 |
| 138 | Yue Tai B | China | MV | I | G | 1.27 |
| 139 | Yun Li 103 | China | MV | I | A | 1.88 |
| 140 | Chang Bai 9 | China | MV | J | A | 1.13 |
| 141 | Zheng Dao 88 | China | MV | J | A | 1.07 |
| 142 | Zhong Chao 123 | China | MV | J | A | 1.83 |
| 143 | Zhong Hua 11 | China | MV | J | A | 1.13 |
| 144 | Zhong Hua 8 | China | MV | J | A | 1.36 |

^a^ MV: modern variety; TL: traditional landraces; BL: breeding line
