# Supplementary material for: SS1 (NAL1)- and SS2-Mediated Genetic Networks Underlying Source-Sink and Yield Traits in Rice (Oryza sativa L.)
Source: PLoS One. 2015 Jul 10;10(7):e0132060. doi: 10.1371/journal.pone.0132060 (PMC4498882; doi:10.1371/journal.pone.0132060)
Supplement: S10 Table — (DOC) [file pone.0132060.s018.doc]

**S10 Table** Phenotypic differences between Teqing (TQ) and its *SS1* near isogenic line (NIL-*SS1*) and Lemont (donor, LT) for SS and yield traits in Hangzhou, Beijing and Sanya

| Line | Location | PH^a^  (cm) | PP | FLW (cm) | FLL  (cm) | PL  (cm) | GNP | FGP | GW  (g) | GL  (mm) | GWH  (cm) | GY  (g/plant) | GYP^b^  (kg/plot) | Compared with TQ (**±**%) |
| --- | --- | --- | --- | --- | --- | --- | --- | --- | --- | --- | --- | --- | --- | --- |
| TQ | Hangzhou | 109.9 | 10.1 | 1.8 | 30.4 | 20.7 | 216.2 | 171.5 | 26.1 | 7.7 | 3.2 | 31.7 | 11.2 | - |
| NIL-*SS1* | Hangzhou | 110.7 | 8.6 | **2.0*^c^** | 33.2 | 21.2 | **281.3**** | **199.2*** | 26.3 | 7.7 | 3.3 | 33.1 | 11.6 | 3.57 |
| LT (CK) | Hangzhou | 93.9 | 8.6 | 2.0 | 24.1 | 23.5 | 119.7 | 101.3 | 26.0 | 9.4 | 2.6 | 21.1 | 6.9 | -38.1 |
| TQ | Beijing | 96.4 | 8.6 | 1.9 | 34.8 | 21.8 | 306.3 | 236.3 | 23.4 | 7.3 | 3.1 | 32.1 | 13.3 | - |
| NIL-*SS1* | Beijing | 98.1 | 7.4 | **2.1*** | 35.5 | 21.7 | **351.3*** | **293.5**** | 23.5 | 7.3 | 3.1 | 34.4 | 13.8 | 3.81 |
| LT (CK) | Beijing | 86.9 | 10.3 | 2.2 | 27.5 | 24.2 | 155.2 | 136.2 | 23.2 | 9.3 | 2.5 | 21.4 | 8.2 | -38.4 |
| TQ | Sanya | 95.4 | 10.9 | 1.5 | 27.9 | 19.2 | 251.1 | 230.9 | 23.9 | 7.5 | 3.1 | 28.7 | 9.3 | - |
| NIL-*SS1* | Sanya | 95.2 | 9.0 | **1.7*** | 27.4 | 18.4 | **286.8*** | **254.6*** | 23.0 | 7.5 | 3.2 | 29.6 | 9.6 | 3.23 |
| LT (CK) | Sanya | 89.9 | 10.1 | 1.8 | 20.3 | 23.1 | 144.4 | 128.5 | 23.5 | 9.3 | 2.6 | 19.5 | 6.5 | -30.1 |

^a^ PH: plant height, PP: productive panicles per plant, FLW: flag leaf width, FLL: flag leaf length, PL: panicle length, GNP: grain number per panicle, FGP: filled grains per panicle, GW: 1000-grain weight, GL: grain length, GWH: grain width, GY: grain yield per plant, GYP: grain yield per plot

^b^ Plot size is 13.3 m^2^.

^c^ *,** represent significant differences at P≤0.05, 0.01, respectively.
