# Supplementary material for: SS1 (NAL1)- and SS2-Mediated Genetic Networks Underlying Source-Sink and Yield Traits in Rice (Oryza sativa L.)
Source: PLoS One. 2015 Jul 10;10(7):e0132060. doi: 10.1371/journal.pone.0132060 (PMC4498882; doi:10.1371/journal.pone.0132060)
Supplement: S11 Table — (DOC) [file pone.0132060.s019.doc]

**S11 Table** Flag leaf net photosynthetic rate *(P_n_*, µmol CO_2_ m^-2^s^-1^), stomatal conductance (*g_s_*, mol H_2_Om^-2^s^-1^), intercellular CO_2_ concentration (*C_i_*, µmol CO^-2^mol^-1^), transpiration rate (*T_r_*, mmol H_2_Om^-2^s^-1^), and specific leaf weight (SLW, mg cm^-2^) of leaf area of the two parents (Teqing and Lemont) and NIL*-SS1* evaluated under the irrigated condition in Beijing

| Year | Trait | Stage^a^ | Teqing^b^ | NIL*-SS1* | Lemont |
| --- | --- | --- | --- | --- | --- |
|  |  |  | Mean±SD | Mean±SD | Mean±SD |
| 2010 | *P_n_* | TS | **26.6±0.7a** | **23.3±2.2b** | 25.7±0.9a |
|  |  | BS | **15.7±0.5a** | **13.8±0.1b** | **14.3±1.1ab** |
|  |  | FS | 22.1±3.4 | 19.7±3.1 | 18.8±0.3 |
|  |  | GFS | **10.2±0.7b** | **13.1±1.5a** | **14.9±1.5a** |
|  | *g_s_* | TS | 0.72±0.12 | 0.54±0.15 | 0.81±0.23 |
|  |  | **BS** | **0.32±0.02a** | **0.23±0.01b** | **0.34±0.07a** |
|  |  | FS | 0.57±0.08 | 0.50±0.04 | 0.48±0.06 |
|  |  | **GFS** | **0.16±0.03b** | **0.23±0.02a** | **0.25±0.05a** |
|  | *C_i_* | TS | 258.8±5.24 | 248.6±4.4 | 258.0±6.6 |
|  |  | BS | 215.0±2.19 | 214.2±5.4 | 228.6±12.2 |
|  |  | FS | 281.9±3.69 | 269.0±8.2 | 270.7±8.6 |
|  |  | GFS | 241.8±2.06 | 234.8±8.1 | 224.9±12.1 |
|  | *T_r_* | TS | 12.2±2.3 | 9.3±1.5 | 10.8±1.10 |
|  |  | BS | 10.5±1.5 | 9.3±0.2 | 10.6±0.62 |
|  |  | FH | 8.1±1.1 | 7.3±1.0 | 7.0±1.10 |
|  |  | GFS | 7.0±1.16 | 8.4±1.6 | 8.9±1.95 |
| 2011 | *P_n_* | BS | **21.4±1.9a** | **18.5±1.9b** | **17.4±2.0c** |
|  |  | FS | **21.8±1.7a** | **19.1±1.0b** | **17.1±3.5c** |
|  |  | 7 DAF | 22.6±2.0a | 21.6±1.6a | 13.6±3.1b |
|  |  | **14 DAF** | **20.6±1.8a** | **18.5±1.2b** | **13.8±1.9c** |
|  |  | 21 DAF | 13.5±1.5 | 14.0±1.9 | 13.8±1.9 |
|  | *g_s_* | BS | **0.80±0.18a** | **0.66±0.13b** | **0.36±0.07c** |
|  |  | FS | 0.59±0.13a | 0.62±0.07a | 0.39±0.08b |
|  |  | 7 DAF | 0.50±0.06 | 0.49±0.05 | 0.54±0.11 |
|  |  | 14 DAF | 0.73±0.18a | 0.65±0.12a | 0.16±0.03b |
|  |  | 21 DAF | 0.64±0.14a | 0.61±0.10a | 0.41±0.10b |
|  | *C_i_* | BS | 281.9±18.4a | 280.6±11.4a | 258.1±15.0b |
|  |  | FS | **283.4±6.3b** | **296.1±7.9a** | 293.3±15.3a |
|  |  | 7 DAF | 281.3±12.2a | 281.8±12.9a | 332.3±17.0b |
|  |  | 14 DAF | 312.7±8.6a | 310.8±8.3a | 203.1±17.3b |
|  |  | 21 DAF | 342.5±11.9 | 338.0±10.5 | 338.7±10.7 |
|  | *T_r_* | BS | **9.7±1.1a** | **8.6±0.7b** | **7.0±0.9c** |
|  |  | FS | 9.1±0.9a | 8.9±0.5a | 6.6±0.9b |
|  |  | 7 DAF | 10.6±0.8a | 10.3±0.9a | 6.1±0.6b |
|  |  | 14 DAF | 9.9±1.4a | 9.1±0.8a | 3.9±0.6b |
|  |  | 21 DAF | 6.8±0.71a | 6.5±0.5a | 5.2±0.6b |
|  | SLW | BS | 3.8±0.7a | 3.4±0.5b | 3.2±0.4b |
|  |  | FS | 4.6±0.9a | 3.6±0.7b | 3.7±0.6b |
|  |  | 7 DAF | 5.2±1.0a | 4.2±0.9b | 4.1±0.8b |
|  |  | 14 DAF | 5.4±0.8a | 4.2±0.8b | 4.1±0.7b |
|  |  | 21 DAF | 5.2±0.7 | 4.6±0.4 | 4.5±0.9 |
| 2012 | *P_n_* | BS | 20.8±2.6 | 20.7±2.9 | 22.1±2.3 |
|  |  | FS | 23.3±2.2b | 23.8±2.9b | 31.6±2.0a |
|  |  | 7 DAF | 30.6±2.2 | 30.4±2.7 | 29.2±1.9 |
|  |  | 14 DAF | 28.6±1.2a | 28.1±1.8a | 24.2±2.6b |
|  |  | 21 DAF | 23.1±3.4 | 23.9±1.7 | 21.2±3.0 |
|  | *g_s_* | BS | 0.34±0.08b | 0.36±0.11b | 0.45±0.10a |
|  |  | FS | 0.50±0.11 | 0.49±0.11 | 0.46±0.17 |
|  |  | 7 DAF | 0.47±0.10 | 0.46±0.12 | 0.42±0.11 |
|  |  | 14 DAF | 0.49±0.04a | 0.47±0.07a | 0.25±0.08b |
|  |  | 21 DAF | 0.29±0.08a | 0.29±0.04a | 0.43±0.10b |
|  | *C_i_* | BS | 220.8±12.0b | 222.1±20.0b | 254.6±11.7a |
|  |  | FS | 259.4±13.5a | 252.4±15.5a | 236.4±32.3b |
|  |  | 7 DAF | 254.1±16.3a | 247.4±30.4a | 221.3±28.0b |
|  |  | 14 DAF | 251.3±4.1a | 243.1±22.1a | 171.7±42.8b |
|  |  | 21 DAF | 209.1±36.1a | 207.2±17.2a | 284.5±23.4b |
|  | *T_r_* | BS | 8.0±1.2 | 8.1±1.6 | 7.2±0.8 |
|  |  | FS | 8.3±1.4b | 7.9±1.2b | 11.1±2.2a |
|  |  | 7 DAF | 11.5±1.3a | 11.3±1.8a | 8.8±1.2b |
|  |  | 14 DAF | 10.0±0.5a | 9.0±0.8a | 4.7±1.1b |
|  |  | 21 DAF | 5.0±1.1a | 5.1±0.6a | 4.0±0.6b |

^a^ TS: the tillering stage; BS: the booting stage; FS: the flowering stage; GFS: the grain filling stage; 7 DAF, 14 DAF and 21 DAF: 7, 14 and 21 days after flowering, respectively.

^b^ Different letters after the numbers indicate significant differences between or among the three genotypes at P ≤ 0.05, based on the Duncan multiple tests.
